# A whole blood gene expression-based signature for smoking status

**DOI:** 10.1186/1755-8794-5-58

**Published:** 2012-12-03

**Authors:** Philip Beineke, Karen Fitch, Heng Tao, Michael R Elashoff, Steven Rosenberg, William E Kraus, James A Wingrove

**Affiliations:** 1 CardioDx, Inc., 2500 Faber Place, Palo Alto, CA, 94303, USA; 2 Department of Cardiology and Center for Genomic Medicine, Duke University School of Medicine, Durham, NC, 27710, USA

**Keywords:** Smoking, Gene expression, Coronary artery disease, Whole blood

## Abstract

**Background:**

Smoking is the leading cause of preventable death worldwide and has been shown to increase the risk of multiple diseases including coronary artery disease (CAD). We sought to identify genes whose levels of expression in whole blood correlate with self-reported smoking status.

**Methods:**

Microarrays were used to identify gene expression changes in whole blood which correlated with self-reported smoking status; a set of significant genes from the microarray analysis were validated by qRT-PCR in an independent set of subjects. Stepwise forward logistic regression was performed using the qRT-PCR data to create a predictive model whose performance was validated in an independent set of subjects and compared to cotinine, a nicotine metabolite.

**Results:**

Microarray analysis of whole blood RNA from 209 PREDICT subjects (41 current smokers, 4 quit ≤ 2 months, 64 quit > 2 months, 100 never smoked; NCT00500617) identified 4214 genes significantly correlated with self-reported smoking status. qRT-PCR was performed on 1,071 PREDICT subjects across 256 microarray genes significantly correlated with smoking or CAD. A five gene (CLDND1, LRRN3, MUC1, GOPC, LEF1) predictive model, derived from the qRT-PCR data using stepwise forward logistic regression, had a cross-validated mean AUC of 0.93 (sensitivity=0.78; specificity=0.95), and was validated using 180 independent PREDICT subjects (AUC=0.82, CI 0.69-0.94; sensitivity=0.63; specificity=0.94). Plasma from the 180 validation subjects was used to assess levels of cotinine; a model using a threshold of 10 ng/ml cotinine resulted in an AUC of 0.89 (CI 0.81-0.97; sensitivity=0.81; specificity=0.97; kappa with expression model = 0.53).

**Conclusion:**

We have constructed and validated a whole blood gene expression score for the evaluation of smoking status, demonstrating that clinical and environmental factors contributing to cardiovascular disease risk can be assessed by gene expression.

## Background

Tobacco use results in over 5 million deaths on an annual basis and is the leading cause of preventable death worldwide [1]. Exposure to tobacco smoke, by both active and passive means, contributes to the increased risk and development of numerous diseases, including asthma, chronic obstructive pulmonary disease (COPD), and several types of cancer [2]. A strong association also exists between smoking and cardiovascular disease; up to an 80% increased risk is observed for active smokers and 30% for passive smokers [3]. Acute coronary syndromes (ACS), stable angina, stroke, carotid and peripheral atherosclerosis are all increased in smokers [3]. Driving increased coronary disease risk are physiological changes that occur in response to smoking, including lipid oxidation/modification, vasomotor dysfunction, and inflammation [3].

Changes in gene expression in peripheral blood cells correlate with a number of systemic inflammatory and immune-related disorders, including cardiovascular disease [4-8]. We have recently described the development and validation of a peripheral blood gene expression score (GES) for the assessment of the likelihood of obstructive CAD in non-diabetic patients [7,9]. The GES is derived from the expression levels of 23 genes as well as age and sex; the genes are grouped into highly correlated meta-genes which reflect both biological processes and cell type prevalence [7,9]. The GES is related to the current likelihood of obstructive CAD [7,9].

To better understand the physiological alterations induced by smoking and their relation to the development of CAD, we sought to identify changes in whole blood gene expression that correlate with self-reported smoking status. Herein we describe a set of genes expressed in whole blood that are strongly affected by smoking, and the development of a gene expression signature that is predictive of self-reported smoking status.

## Methods

### Patient selection and clinical methods

All patients were clinically referred for invasive angiography; angiograms were performed based on local, institutional protocols. The microarray cohort of 210 subjects (110 case:control pairs, matched for age and sex) and the qRT-PCR algorithm development and validation cohorts (1,071, 180 respectively) were part of PREDICT, a multi-center US study of patients referred for coronary angiography ( http://www.clinicaltrials.gov, NCT00500617). The institutional review boards at all centers approved the study, and all patients gave written informed consent. Quantitative coronary angiography (QCA) was performed on all subjects as previously described [9].

### Blood collection and RNA purification

Whole blood samples were collected in PAXgene® and EDTA tubes prior to coronary angiography. PAXgene® tubes were processed according to the manufacturer’s instructions, then frozen at −20°C. RNA was purified as previously described, using the Agencourt RNAdvance system [9] Plasma was isolated from EDTA tubes by centrifugation at 1800 *g* for 10 min, followed by the removal of the upper plasma layer and subsequent storage at −80°C.

### Microarray methods

Microarray samples were labeled and hybridized to 41K Human Whole Genome Arrays (Agilent, PN #G4112A) using the manufacturer’s protocol. Microarray data sets have been deposited in GEO (GSE 20686). Agilent processed signal values for array normalization were scaled to a trimmed mean of 100 and then log_2_ transformed. Standard array QC metrics (percent present, pair-wise correlation, and signal intensity) were used for quality assessment. Quantile normalization was subsequently used to further normalize the data [10].

### Microarray analysis

To identify genes associated with smoking status, logistic regression was performed, adjusting for age and sex. Gene Set Enrichment Analysis (GSEA) was performed with 4 different gene sets (curated gene sets = 3272 sets; motif gene sets = 836 sets; computational gene sets = 881 sets; GO gene sets = 1454 sets) using 1000 permutations^13^; BINGO was used to assess enrichment of gene ontology terms in the set of 4214 significant array genes; a hypergeometric test was used to identify overrepresented terms and results were corrected for multiple testing using Benjamini & Hochberg False Discovery Rate (FDR) [11]. Hierarchical clustering was performed using Gene Cluster 3.0 using mean-centered expression data in a complete linkage, correlation-based approach [12]; clusters were visualized using Java Treeview [13]. The cell-type specificity of gene expression was evaluated using whole-blood normalized expression values derived from BioGPS [14].

### Gene selection

Genes for qRT-PCR were selected from the microarray data based on statistical significance, gene ontology pathway analysis, and literature support.

### qRT-PCR

Amplicon design and cDNA synthesis were performed as previously described [7,8] qRT-PCR was performed on the Biomark microfluidic platform (Fluidigm, South San Francisco, CA). Prior to PCR, 2.5ul of cDNA was pre-amplified for 18 cycles using TaqMan® PreAmp Master Mix (Life Technologies, Carlsbad, CA) in a 10 ul reaction volume. PCR reactions were run in duplicate on Fluidigm 96X96 microfluidic gene expression chips, and median Cp values used for analysis.

### Statistical methods

Clinical/demographic factors were assessed for self-reported smoking status association using univariate logistic regression. Gene expression association with smoking status was assessed by logistic regression (sex/age adjusted). All statistical methods were performed using either the R software package, v. 2.09 or Minitab, v. 15.1.3.

### Algorithm development and validation

Expression values for the 256 qRT-PCR genes were normalized to the mean of ACLY and TFCP2, two low-variability genes whose expression levels had previously been observed to correlate with laboratory processing effects. In a given sample, expression values for genes were truncated if values exceeded the 0.01 and 0.99 quantile. A predictive model was fit and cross-validated (10 fold, 1000 iterations) via forward stepwise logistic regression. Candidate predictors included all genes and also patient age and sex. The binary response variable (current/recent smokers vs. former and non-smokers) and 0.5 probability cut-point were prospectively defined for the analysis of the validation set. The formula for the GES algorithm is: (pr(Smoker)/(1-Pr(Smoker)) = 15.78306 + 0.3876 * SEX – 3.3368 * CLDND1-3.4034*LRRN3-1.4847 * MUC1 + 5.9209 * GOPC + 2.27166 * LEF1 where SEX =1 if male, 0 if female.

### Cotinine assay

Plasma cotinine levels were measured in 180 PREDICT subjects using a commercially available ELISA assay (Calbiotech, Spring Valley, CA), following the manufacturer's recommended procedure.

## Results

### Microarray identification of genes responsive to smoking

Whole genome microarray analysis was performed on 210 subjects of which self-reported smoking status was available on 209. Forty-one of the subjects were current smokers, 4 had recently quit (within 2 months), 64 were former smokers (quit longer than 2 months) and 100 reported that they had never smoked; full demographics are given in Table 1. Maximum coronary artery stenosis (as defined by quantitative coronary angiography), age, and neutrophil count were all significantly associated with smoking status (Table 1). 5096 probes mapping to 4214 unique genes were significantly associated with smoking status in a sex- and age-adjusted logistic regression model (p < 0.05, Additional file 1: Table S1); of the 4214 genes, 39% (1649) were down-regulated in response to smoking status whereas 61% (2565) were up-regulated. The most significant associations with smoking status were observed in two up-regulated genes (LRRN3, CLDND1) both of which remained significant after adjusting for multiple testing (p < 1.22 x 10^-6^).

**Table 1:** Clinical demographics of microarray subjects

	**Never**	**Former**	**Recent**	**Current**	**p value**^†^
Variable^*^	(N = 100)	(N = 64)	(N = 4)	(N = 41)	
Max QCA^‡^	42.54±37	37.71±36	47.3±44	57.13±37	**0**.**029**
Sex (%Male)	67 (0.67)	54 (0.84)	3 (0.75)	34 (0.83)	0.063
Age (yrs)	59±13	63±11	53±14	54±11	**0**.**006**
Caucasian (%)	89 (0.89)	61 (0.95)	3 (0.75)	35 (0.85)	0.374
BMI	30±6	30±5	33±8	30±8	0.452
Systolic BP	136±18	133±18	130±20	135±15	0.541
Diastolic BP	83±12	79±12	78±10	80±11	0.242
Hypertension (%)	62 (0.62)	37 (0.578125)	2 (0.5)	20 (0.49)	0.180
Dyslipidemia (%)	63 (0.63)	36 (0.5625)	2 (0.5)	23 (0.56)	0.308
Neutrophil Count	3.9±1.2	3.8±1.3	4.8±1	4.8±1.7	<**0**.**001**
Lymphocyte Count	1.9±0.5	1.9±0.7	2±0.2	2.1±0.7	0.063

^*^Mean values are given, ± SD or % in parenthesis.

^†^ Variables with p values in **bold** are significantly different between the four categories (< 0.05).

^‡^Maximum coronary artery stenosis in all major coronary vessels of a subject, as determined by quantitative coronary angiography (QCA).

**Figure 1:**
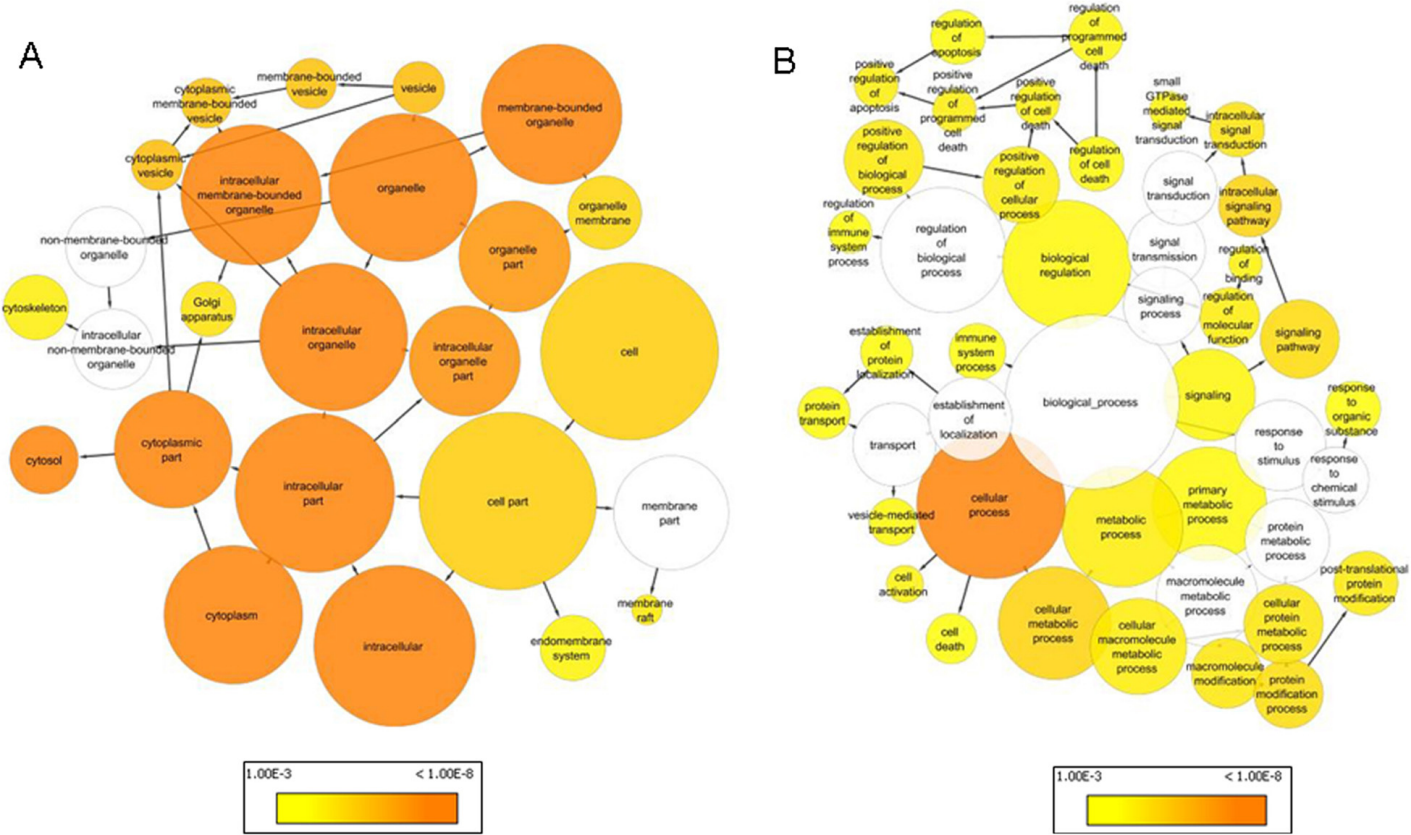
**Gene ontology analysis of 4214 array genes associated with smoking.** The 4214 smoking-associated genes were analyzed using BINGO to identify significant biological processes. Significant processes (p < 0.001 after FDR correction) are colored with the gradient of p values reflected in the colors as indicated, and the biological process annotated. (**A**) Cellular component ontological terms (**B**) Biological Process ontological terms.

To investigate associations of the 4214 genes with biological pathways and networks, the log-odds values from the logistic regression model were used in a Gene Set Enrichment Analysis (GSEA) [15]. This analysis however did not yield any gene sets with a FDR of less than 0.25 (Additional file 1: Table S2). To further evaluate pathways and networks associated with the array genes, enrichment of gene ontology terms was evaluated [11]. Two molecular function categories, 189 biological process categories, and 60 cellular component categories showed significant enrichment in a Bonferroni-corrected analysis (corrected p value < 0.05, Additional file 1: Table S3). The most significant categories included regulation of apoptosis, cell death, regulation of immune system process, and response to organic substance (Figure 1A, B; p < 0.001).

**Figure 2:**
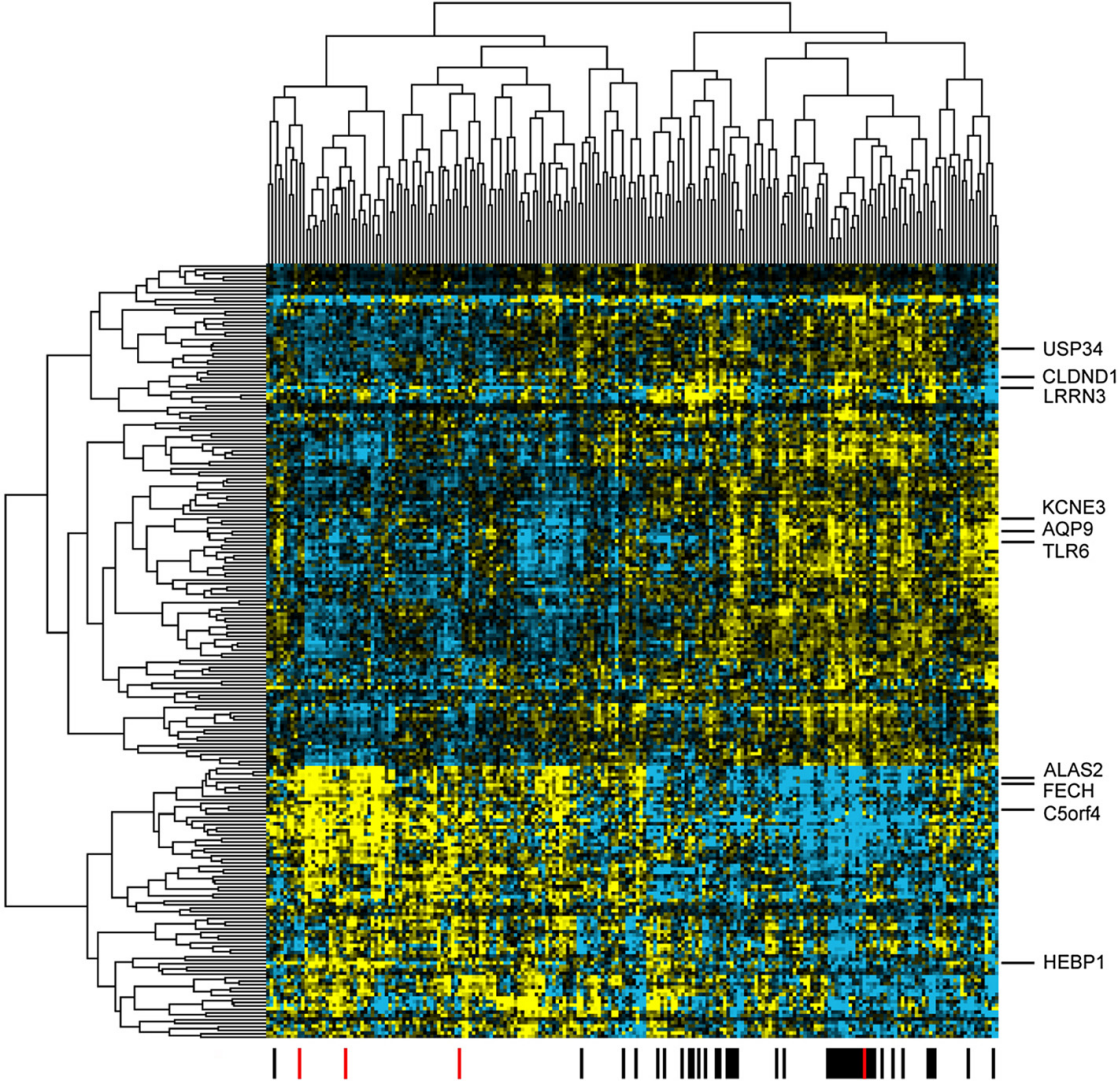
**Hierarchical clustering of 209 subjects and 227 array genes associated with smoking (p < 0.001).** The dendogram on top shows correlations between subjects; black bars at bottom denote current smokers; red bars denote recently quit smokers. Dendogram on the left shows correlations between genes; positions of representative cell-specific genes are shown on the right.

To identify groups of correlated genes and subjects in the microarray dataset, hierarchical clustering was performed on the subjects and the subset of 227 genes showing the most significant association with smoking (p < 0.001, Figure 2). The subjects fell into two main clusters, with 37 (90%) of the current smokers partitioning into one cluster (Figure 2). The genes also partitioned into two main clusters; the larger cluster consisting of genes up-regulated in response to smoking, the smaller cluster containing down-regulated genes (Figure 2, top and bottom cluster respectively). Cell type-specific gene expression analysis of the clusters was investigated using data from BioGPS, which was available for 172 of the genes (Additional file 1: Table S4) [14]. The up-regulated cluster contained genes expressed in myeloid (e.g. KCNE3, AQP9, TLR6) and lymphoid (e.g. CLDND1, LRRN3, USP34) cells, whereas the down-regulated cluster was enriched in genes expressed strongly in hematopoietic-stem cells and early erythroid cells (e.g. ALAS2, FECH, C5orf4, HEPB1).

### Validation of array genes responsive to smoking by qRT-PCR

**Figure 3:**
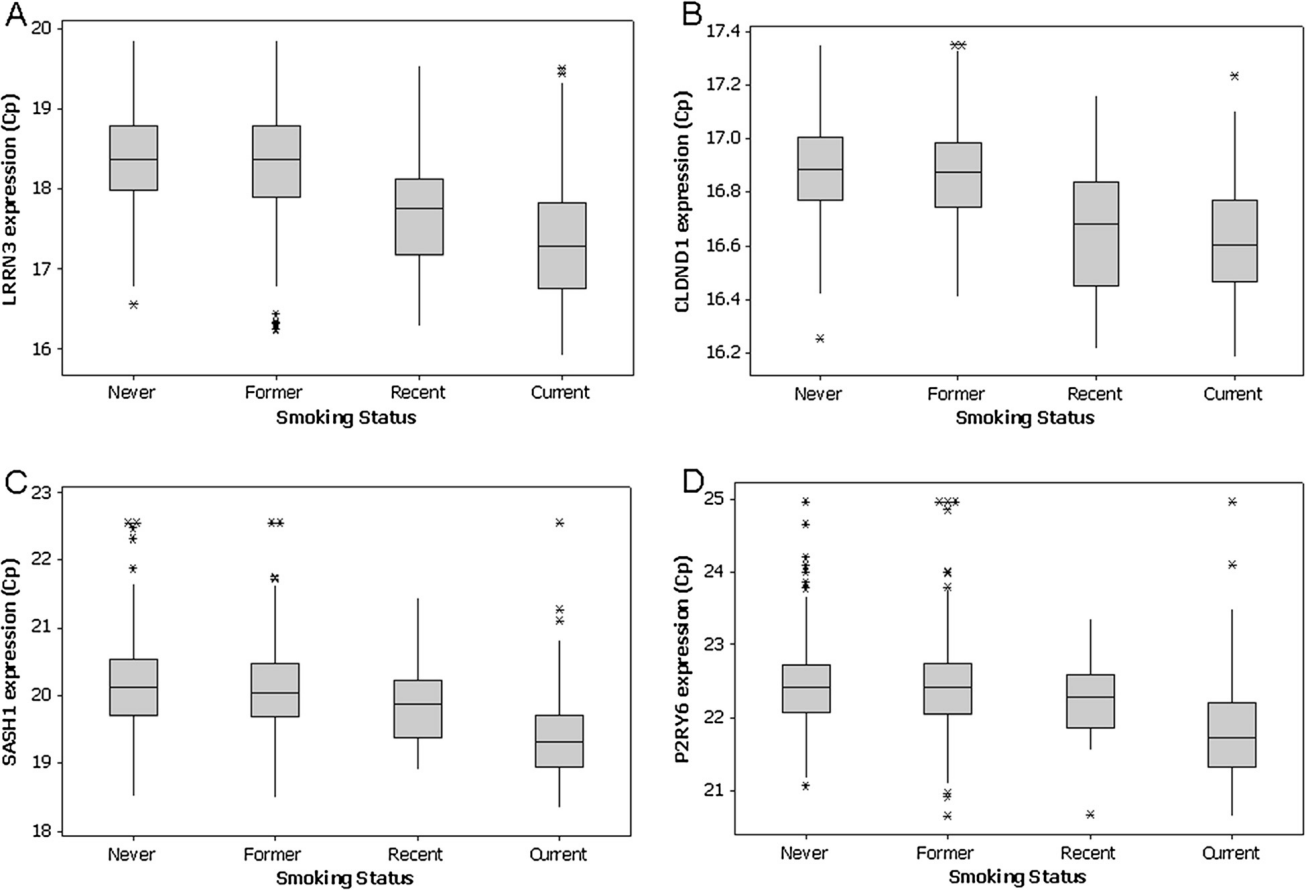
**Expression levels of four most significant genes as assessed by qRT-PCR across 1074 PREDICT subjects grouped by self-reported smoking status.** Expression levels are shown in Cp units on the Y axis, self-reported smoking status is shown on the X axis. (**A**) LRRN3; (**B**) CLDND1; (**C**) SASH1; (**D**) P2RY6.

256 genes were selected from the microarray data based on association with smoking or coronary artery disease (CAD). The expression levels of the 256 genes were evaluated across 1071 PREDICT subjects, 201 (19%) of which were self-reported smokers, 352 former smokers, 30 recently quit, and 488 subjects who had never smoked. Of the 256 genes, 53% (135) showed a significant association with smoking status in an age- and sex-adjusted logistic regression model; 74% (59) of the 80 significant array genes remained significant by qRT-PCR (Additional file 1: Table S5). Of this set, all but 3 (HIST1H2AC, NONO, PAPD4) agreed with the array data in directionality of gene expression. LRRN3 remained the gene most significantly associated with smoking status, followed by CLDND1, SASH1, and P2RY6 (p < 0.001, Figure 3).

### Gene expression model development and validation

Step-wise forward logistic regression was utilized to construct a gene-expression model predictive of self-reported smoking status; smoking status was used as the dependent variable, with age, sex and the expression levels of the 256 genes used as independent variables. The model selected five genes (LRRN3, CLDND1, MUC1, GOPC, LEF1); in a cross-validated assessment of model performance in which current and recently quit smokers were combined into one group, and former and never-have-smoked subjects into a second, the model had an AUC of 0.93 (Table 2, Additional file 2: Figure S1A), with a sensitivity of 0.79 and a specificity of 0.95 (cutoff = 50% probability of smoking, Table 2). Model performance was validated using 180 independent PREDICT subjects, with an AUC of 0.82 (95% CI 0.65-0.94), a sensitivity of 0.63 and a specificity of 0.94 (Table 2, Additional file 2: Figure S1B).

**Table 2:** Performance of GES and cotinine models

	AUC^*^	**Sensitivity**	**Specificity**
GES – Development Set	0.93	0.79	0.95
GES – Validation Set	0.82 (0.65-0.94)	0.63	0.94
Cotinine – Validation Set	0.89 (0.81-0.97)	0.81	0.97

^*^95% confidence interval is shown in parentheses. As the AUC for the development set was derived via cross-validation, a confidence interval could not be assigned. However, the standard deviation of the cross-validation runs equaled 0.03; the standard error equaled 0.001.

### Comparison of gene expression model performance to cotinine

**Figure 4:**
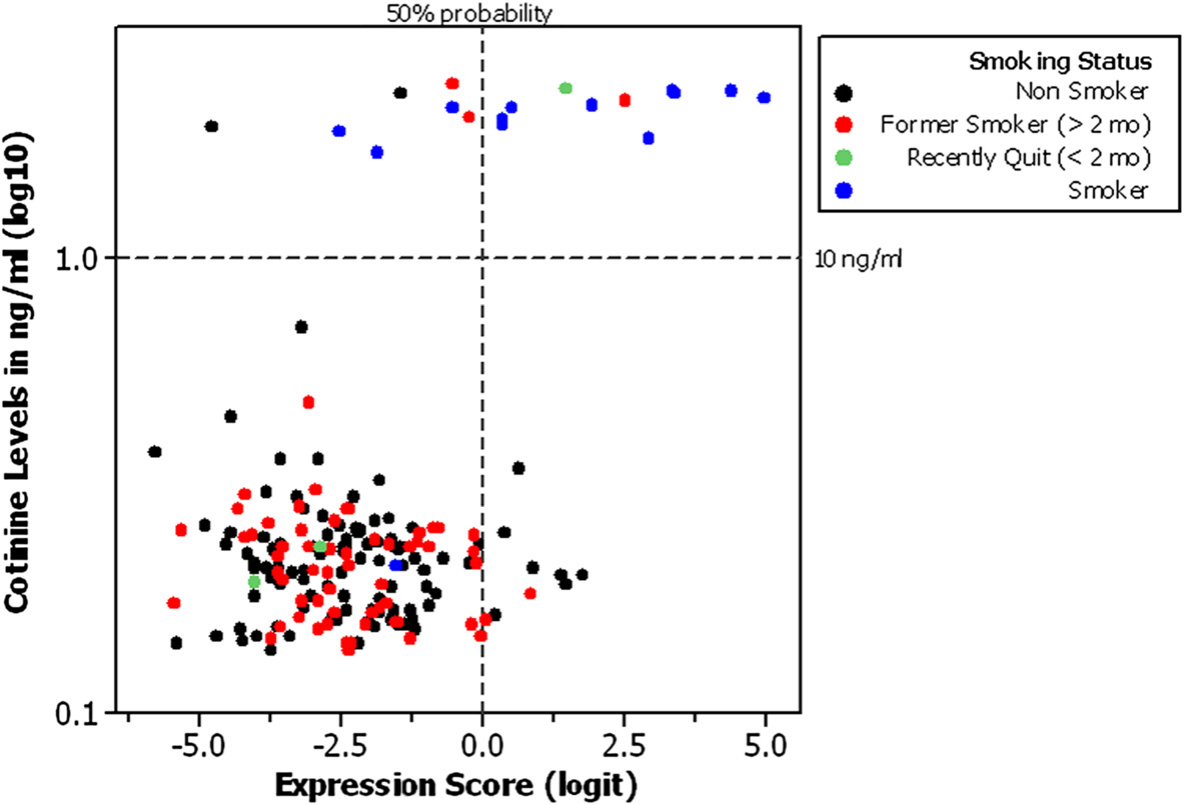
**Comparison of gene expression score to cotinine levels in validation set.** The y-axis shows the log10 value of cotinine levels in the 180 subject validation set; the horizontal dashed line (−−-) denotes the 10ng/ml threshold used in the AUC analysis. The x-axis shows the GES in the 180 subject validation set; the vertical dashed line denotes the 50% probability threshold used in the AUC analysis. Black circles = non-smokers; red circles = former smokers (> 2 months quit); green circles = recently quit smokers (< 2 months quit); blue circles = current smokers. All smoking categories are self-reported.

The level of cotinine, a nicotine metabolite commonly used to determine smoking status, was assessed by ELISA assay across the 180 validation subjects [16]. Using a pre-specified threshold of 10 ng/ml, cotinine levels provided an AUC of 0.89 (95% CI 0.81-0.97), a sensitivity of 0.81 and a specificity of 0.97 (Table 2, Additional file 2: Figure S1C). Moderate concordance was observed between the gene expression model and cotinine (91% agreement, 95% CI 85.97-94.83, kappa = 0.53; Figure 4); where both methods reported positive smoking status, 85% (11) subjects were self-reported smokers, 1 had recently quit, and 1 was a former smoker (Figure 4, upper right quadrant).

## Discussion

This study presents gene discovery from microarrays and the development and validation from a large qRT-PCR data set of a whole blood-derived, qRT-PCR based gene expression score for the assessment of smoking status. The initial microarray analysis identified 4214 genes associated with self-reported smoking status. A number of biological pathways known to be affected by smoking showed GO enrichment within this set of genes, including apoptosis and cellular death, immune system development, leukocyte activation, hemopoiesis, stress response, and alterations in platelet activity (Additional file 1: Table S3) [11]. When clustered, the most significant array genes partitioned into two main groups, which appeared to be partially driven by cell-type expression (Figure 3); notably most of the down-regulated genes appeared to be predominantly expressed in CD71+ and CD105+ cell types (Additional file 1: Table S4).

The majority of the genes selected to be analyzed by qRT-PCR (53%) showed a significant association with smoking. Expression levels of the most significant genes (e.g. LRRN3, CLDND1) were roughly equivalent in former smokers and subjects that had never smoked; likewise recently quit smokers appeared more like current smokers (Figure 3). In former smokers gene expression decreased with time elapsed since smoking cessation, however it did not reach non-smoker levels, suggesting that although the impact of smoking on gene expression diminishes over time, it may never be completed resolved (Figure 3). Alternatively, there may be a genetic effect on gene expression levels for genes that are associated with the ability to stop smoking. Prospective studies would be required to specifically dissociate these two possibilities.

The performance of the gene expression model remained fairly consistent across both the development set and validation sets, with a lower AUC seen in the validation set (Table 2). In both sets of subjects the expression model showed higher specificity and negative predictive value (NPV) versus sensitivity and positive predictive value (PPV). The use of cotinine levels to classify subjects provided a better AUC (Table 2), showing moderate concordance with the gene expression model (91% agreement, 95% CI 85.97-94.83; kappa = 0.53, p < 0.001, Figure 4). Interestingly, both methods produced independent sets of false positives (4 subjects by cotinine, 9 by GES; top left and bottom right quadrants, Figure 4). Levels of cotinine are elevated in passive smokers, and it is likely that gene expression may also be sensitive to second-hand smoke or other environmental factors [16,17].

This study had a number of limitations. Self-reported smoking status is an imperfect gold-standard as subjects may not report their status correctly. The number of subjects in certain groups (e.g. recently quit) was limiting; larger numbers might have allowed for identification of better classifiers. A strong CD105+/CD71+ signature was seen in the microarray data, and although genes associated with this array signature were assessed by qRT-PCR (e.g. C5orf4), they were not chosen during model development; it is possible that other candidates from this group could add to algorithm performance. Clinical data relating to some aspects of smoking status was limited; lack of details regarding packs per day or date of smoking cessation prevented identification of subtler changes in gene expression in response to smoking, and lack of data for second-hand smoke exposure prevented assessment of this contribution to changes in gene expression. Finally, this study was not designed to assess whether the observed changes in gene expression were a result of direct exposure of circulating cells to toxins, or due to interactions with damaged lung tissue.

A GES for the determination of smoking status has limited clinical value per se, as self-reported smoking status is fairly reliable. One of the main goals of this study was to identify gene expression changes that correlate with smoking in the hope of understanding the underlying biology of smoking-related diseases. This has been previously done by examining changes in the expression levels of individual genes; the development of a GES however allows for easier comparison to other methods (e.g. cotinine), providing an assessment of the accuracy of gene expression as a marker for smoking [18,19]. In addition, a GES also provides an avenue to assess expression changes in other pulmonary disease cohorts in relation to what is observed with smoking, and may also be useful in examining populations exposed to airborne pollutants.

The biology associated with the genes in the final expression model is intriguing. LRRN3 which encodes a leucine-rich repeat protein, and CLDN1, a claudin-domain containing gene, are both highly expressed in lymphocytes and were previously identified by Charlesworth et al. who used microarrays to examine changes in lymphocyte gene expression in response to smoking [18]. Interestingly, CLDND1 is also up-regulated in lung squamous cell carcinomas [20]. MUC1 encodes a membrane-bound protein that is a member of the mucin family; increases in MUC1 protein levels are associated with poor prognosis of non–small cell lung cancer [21]. GOPC, a coiled-coil motif and PDZ containing protein, negatively regulates CFTR, mutations in which result in cystic fibrosis [22]. Finally, LEF1 is a transcriptional enhancer also highly expressed in lymphocyte cells and is involved in the Wnt signaling pathway [23].

It is interesting to speculate on the relation between the observed changes in gene expression and the development of smoking-associated diseases. Expression levels of CLDND1 remain significantly associated with the presence of CAD in a multivariable model adjusting for smoking status as well as age and sex (unpublished observation); it remains to be determined whether these changes are causal or merely reflective. Likewise, changes in the expression levels of both CLDND1 and MUC1 are associated with the development of lung cancer; it would be interesting to assess the performance of the gene expression model in subjects with other smoking-related diseases such as lung cancer, asthma, and COPD. The validation set contained a number of subjects with false positive and false negative results assigned by both the gene expression model and cotinine; it would be interesting to study whether disease risk was altered in such patients.

## Conclusion

Using microarray and qRT-PCR data sets, comprising over 1000 patients, we have investigated the relationship between peripheral blood cell gene expression and smoking status and derived a gene-expression based algorithm consisting of 5 genes which can accurately assign smoking status to patients. While others have reported the effect of smoking on gene expression in lymphocytes and monocyte-derived macrophages, to our knowledge the current study is the first to look at such changes in RNA isolated from whole blood and to derive a predictive GES [18,19]. Further investigation into the biology behind the genes identified in this study may shed additional light on the relationship between smoking and increased cardiovascular disease risk, and assessment of the performance of the expression model in patients with other smoking-related disorders such as asthma, COPD, and lung cancer might lead to new diagnostic methods for these conditions.

## Abbreviations

CAD: Coronary artery disease; COPD: Chronic obstructive pulmonary disease; ACS: Acute coronary syndrome; GES: Gene expression score; AUC: Area under the curve.

## Competing interests

PB, KF, HT, MRE, SR, and JAW are employees of CardioDx, Inc and have equity and/or stock options in CardioDx. PB, MRE, SR and JAW have filed patent applications on behalf of CardioDx. WEK has received research support from CardioDx, Inc.

## Authors’ contributions

PB, KF, HT, MRE, SR and JAW contributed to the conception, design, and data analysis for this work as well as drafting and approving the final manuscript. WEK helped critically revise the manuscript and all authors approved the final version.

## Pre-publication history

The pre-publication history for this paper can be accessed here:

http://www.biomedcentral.com/1755-8794/5/58/prepub

## Supplementary Material

Additional file 1 **Table S1.** The 5096 microarray features significantly associated with smoking status (sex- and age-adjusted logistic regression model, p < 0.05). **Table S2.** Biological pathways and networks identified through Gene Set Enrichment Analysis associated with significant microarray genes. **Table S3.** Gene ontology terms associated with significant microarray genes (Bonferroni-corrected, p < 0.05). **Table S4.** Cell type-specific gene expression of most significant microarray genes (p < 0.001). **Table S5.** The 256 genes evaluated by qRT-PCR; p values and coefficients are shown for association with smoking status. Click here for file

Additional file 2 **Figure S1.** ROC analysis of gene expression score (GES) and cotinine. Click here for file
