# Supplementary material for: A whole blood gene expression-based signature for smoking status
Source: BMC Med Genomics. 2012 Dec 3;5:58. doi: 10.1186/1755-8794-5-58 (PMC3538056; doi:10.1186/1755-8794-5-58)
Supplement: Additional file 1 — Table S1. The 5096 microarray features significantly associated with smoking status (sex- and age-adjusted logistic regression model, p < 0.05). Table S2. Biological pathways and networks identified through Gene Set Enrichment Analysis associated with significant microarray genes. Table S3. Gene ontology terms associated with significant microarray genes (Bonferroni-corrected, p < 0.05). Table S4. Cell type-specific gene expression of most significant microarray genes (p < 0.001). Table S5. The 256 genes evaluated by qRT-PCR; p values and coefficients are shown for association with smoking status. [file 1755-8794-5-58-S1.doc]

**Supplemental Table 1 - Significant Microarray Genes**

Gene Name Smoking Log Odds Smoking p value

LRRN3 1.78026705 1.87E-07

CLDND1 5.093407199 9.26E-07

SASH1 2.562891905 2.74E-06

OSBPL9 3.892293952 9.86E-06

C5orf4 -1.574913041 1.17E-05

ITLN1 -1.069351883 1.99E-05

MLC1 -2.311309918 2.16E-05

AHRR 7.196156417 3.10E-05

KIT 4.485809571 3.15E-05

AK026750 -1.971008902 3.22E-05

THC2617292 -1.940470039 4.02E-05

LOC100130604 2.957818809 4.08E-05

ADAM23 3.250866657 4.16E-05

HARS2 4.569504576 4.23E-05

TMEM111 -3.025630088 4.66E-05

FANK1 3.531386656 5.49E-05

RNF145 2.686964222 5.70E-05

SLC14A1 -1.189299687 5.75E-05

CNOT3 3.59210265 6.56E-05

JHDM1D 2.819286364 6.68E-05

NCOA6 4.729983996 7.44E-05

PARP8 2.836859617 7.44E-05

PF4 -1.824741949 7.62E-05

RNF34 6.433146281 8.07E-05

LAMP2 2.609814449 8.21E-05

IGF2 -1.365296768 8.23E-05

PPP2R2A 4.356594517 8.23E-05

TLR6 2.060093248 8.34E-05

MSL3L2 3.125129417 8.42E-05

TRAK2 -2.305361943 8.53E-05

KCNQ1OT1 -2.147124695 8.69E-05

RP11-345P4.4 3.483292169 8.69E-05

OPTN -1.735152317 8.93E-05

CDKN1C -1.186510032 9.13E-05

LOC283174 1.877544494 9.67E-05

P2RY5 2.346188169 9.99E-05

LOC390705 2.743514429 0.000116

BACH1 3.078127956 0.000117

ENST00000331447 3.699575637 0.000122

FBXL5 2.397297237 0.000123

P2RY6 3.786808891 0.000126

LOC157627 8.683930362 0.000129

SPHK2 -4.459193549 0.000133

KIAA0515 3.038895724 0.000139

MSL3L1 2.512229452 0.000139

HEBP1 -1.076541661 0.00014

C16orf48 3.092140033 0.000143

SAP130 3.030225293 0.000152

SP3 3.498425091 0.000153

RDH5 3.585996349 0.000155

SSH1 4.292975616 0.000156

RNF111 3.211208858 0.000156

NT5M -1.576271412 0.00016

JARID1B 3.892914723 0.000161

VIL1 -2.247110694 0.000161

CSGALNACT1 1.377344227 0.000162

CCNL1 2.648061582 0.000162

AK098629 -1.64953744 0.000165

SLC6A4 -2.931375632 0.000171

RHOC -2.23929274 0.000177

TMEM71 2.421113187 0.000178

BG461173 7.776168596 0.000183

LARP5 3.702111253 0.000184

GPR20 -1.814211013 0.000191

RBM5 2.95141344 0.000194

HCP5 2.845753775 0.000196

ZFYVE1 4.0417997 0.000196

TESK2 4.373236337 0.000198

PLXNC1 1.852764589 0.000199

TSPAN5 -1.470561382 0.000204

SLC1A7 -1.337951239 0.000206

NR4A1 -1.358116009 0.000208

RGS10 -2.35749868 0.000225

GOLGB1 3.299102345 0.000225

NIN 2.934305356 0.000229

ENST00000390539 0.691880541 0.000231

ZNF281 2.178818306 0.000231

GZMB -1.597573032 0.000232

ZC3H11A 3.830346242 0.000236

FAM104B -1.950986269 0.000253

FZD5 -1.675211402 0.000255

FSTL1 -1.619593738 0.000256

NUP153 3.784330112 0.000262

SH2D1B -1.493534468 0.000266

A_24_P211351 2.977648272 0.000267

ALAS2 -1.091341251 0.000276

ANKHD1-EIF4EBP3 3.010604275 0.000278

GOLGA7 3.054168925 0.00028

THC2532155 -1.749867383 0.000282

PHF20L1 3.016153933 0.000283

FLJ14712 5.205557867 0.000295

BAT2 2.671412665 0.000299

PID1 2.029948176 0.000299

PURB 4.590913201 0.000302

FLCN 6.10722579 0.000302

RRAS -1.78660823 0.000302

C12orf35 1.944666039 0.000307

LGR6 -2.454258994 0.000319

AQP9 1.648073049 0.000322

C15orf29 2.386579479 0.000325

ENST00000400399 5.521446651 0.000332

HSPA1L 3.614835533 0.000332

EGLN1 2.056363404 0.000349

BC020376 5.627539169 0.000353

MAEA 2.554683373 0.000354

PPBP -1.176720333 0.00036

AF086052 1.693849824 0.000361

SYF2 4.255457948 0.000361

CHD2 3.186555159 0.000364

TBRG1 3.738872499 0.000366

THC2691941 2.978113823 0.000368

ENDOD1 -1.93169242 0.000369

ALDH5A1 -3.549094654 0.000385

LOC647121 2.087486042 0.000385

DCUN1D1 -2.23275163 0.000388

PUM2 2.829232352 0.000398

AGTPBP1 9.100692704 0.000402

AF343666 0.977868121 0.000409

SPARC -1.712515511 0.00041

TUBA8 -1.632722997 0.000416

GMIP 2.004295079 0.000419

MNDA 2.233815206 0.000423

C3orf60 -2.983924658 0.00043

ADIPOR1 -1.435300742 0.000432

UBQLN2 3.146131683 0.000442

NOTCH1 2.239737922 0.000445

LOC90379 3.957326309 0.000448

AIM1L 3.08353611 0.00045

ATP5S -1.948422833 0.00045

PSTPIP1 2.236156452 0.000456

THC2662279 3.125812808 0.000457

C18orf17 4.203287212 0.000461

C2orf12 3.242596155 0.000465

AL526449 4.815653209 0.000465

LOC100131993 2.404964703 0.000472

PI16 2.278945264 0.00048

UBE2F -2.098211402 0.000483

LCP2 3.291826958 0.000488

RAB6A -4.045802815 0.000498

THC2697511 -4.866595856 0.000507

GPR171 1.803688833 0.000511

AATK 1.83189967 0.000518

NEURL -1.534012022 0.000523

ACTR3 2.935645069 0.000527

HSPA12A 39.84257419 0.000536

AGER 2.925804067 0.000541

SPON2 -1.085524544 0.000541

UBR2 3.149129948 0.000543

AP3M1 2.718589902 0.000548

AK000925 2.184130787 0.00055

PPFIA1 3.060210553 0.000566

RHOA 3.378828096 0.000567

MGLL -1.614735279 0.000568

USP34 2.398257984 0.000574

A_32_P96124 5.777443575 0.000576

ABCC3 -1.519667327 0.000578

THC2640099 2.362030844 0.00059

PARP16 2.576047915 0.000593

FECH -1.034065204 0.000593

TTC25 -1.249066533 0.000594

PRSS23 -1.494144235 0.000597

RAB27B -2.581914942 0.000602

TAC3 -3.865739699 0.000607

C1orf119 2.826073852 0.000607

EIF4G2 2.954023467 0.000618

ZBTB34 2.55723653 0.000622

THC2499809 -1.545902113 0.00063

MICB 3.584239543 0.000631

BCORL1 2.673549502 0.000639

XR_016510 -4.65616227 0.000639

TESC -1.580813912 0.000649

CREG1 -1.459824141 0.000654

VAMP3 2.138366263 0.000663

PHF17 3.598162791 0.000665

N4BP2L2 2.117888165 0.00067

FAM8A1 2.29906446 0.000671

POP7 -2.615554473 0.000675

MORC3 2.738688073 0.000679

TKT 1.962798395 0.000684

C20orf108 -1.266068254 0.00069

TGFBR2 2.725043452 0.000699

CYB5R3 -2.452533864 0.000713

TMEM45B 1.395281132 0.000726

FBXO11 3.536940486 0.000727

KCNE3 1.842645964 0.000727

IQGAP1 2.119463402 0.000739

SNX13 3.173420923 0.000746

QPRT -2.511507561 0.000748

PRDX6 -1.180737549 0.000763

SPN -2.192343097 0.000764

A_24_P7330 -1.21309642 0.000771

RBX1 -2.27944009 0.000782

LSP1 2.267006826 0.000784

ZCCHC3 3.697070589 0.0008

EVI2A 1.903469467 0.0008

BRCA1 2.442937188 0.000804

PLCG2 2.438967141 0.000804

SESN3 -1.277528732 0.000805

A_32_P219011 2.68593309 0.000813

MBD6 1.571551201 0.000822

NPFF 3.205799441 0.000826

AR -10.70100962 0.000841

NFIX -0.907636708 0.000844

MBP 4.682954537 0.000847

IFITM4P 1.197695297 0.000851

LOC441016 4.060872011 0.000852

CFHR3 3.077929908 0.000863

STX1A -2.72159194 0.000874

CFH 2.375842744 0.000877

TLR1 2.038182567 0.00089

BC000986 -1.444972204 0.000899

BE184907 -1.928908465 0.000908

SETBP1 -1.869444686 0.00091

POLR2C 1.978256063 0.000931

ZNF398 4.107935625 0.000933

FUCA1 1.475471858 0.000941

LIPC -2.354734675 0.000943

A_32_P117908 -2.981056464 0.000945

ST3GAL6 2.333913336 0.000964

KIAA1370 2.074920552 0.000986

PDZK1IP1 -1.102991346 0.000993

TMEM116 2.032606536 0.001

GSK3A -2.939564481 0.001004

SHC1 3.23518153 0.001011

C3orf32 10.01294871 0.001012

AL546498 -1.416891436 0.001019

THC2663297 -1.123114151 0.001027

C14orf159 1.901764596 0.001029

PLCG1 2.996420549 0.00103

MOBKL2A 2.083668871 0.001033

AF119871 5.181428363 0.001033

FLJ35934 -3.850937374 0.001035

ESAM -0.990457205 0.00104

KLC4 4.337891428 0.001041

LIN7A 1.680305636 0.001049

SCN4B 6.662952709 0.001077

FCHO2 1.58457532 0.001082

TMEM55A 2.202382858 0.001083

PJA2 2.074004814 0.001094

YBX1 -1.617465099 0.001098

UBE2G2 -1.560450136 0.0011

MED11 3.126711213 0.0011

RRN3 1.25884931 0.001102

PTCRA -1.166316508 0.001104

GCC1 2.975256341 0.001108

DDIT3 2.40567749 0.001111

VAV1 1.7807376 0.001116

B3GNTL1 2.101938145 0.00112

CSNK1G2 -0.833974138 0.001127

TMEM33 2.541690268 0.001127

ATP6V1A 2.384729558 0.001128

COASY 2.237439764 0.001137

CPEB2 4.535100429 0.001141

X57802 1.121157319 0.001152

LOC100133781 1.940309062 0.001158

SLC22A15 1.918750989 0.001164

THC2665061 -2.576427684 0.001179

CENTB2 2.36438678 0.001181

AK001998 -4.645533389 0.001192

COG8 3.794870283 0.001193

STOM -2.205088184 0.001201

IFITM2 1.24924281 0.001205

ERBB2 -2.17670472 0.001209

FEZ1 -2.19474195 0.00121

GAS5 1.816541433 0.001211

SRCAP 3.158214943 0.001215

GP1BA -1.307136221 0.001216

BC037740 2.903130711 0.001225

OBSCN -1.142383929 0.001231

SEC16B -0.670246746 0.001234

PPM1A -1.620956978 0.001238

TBC1D8 -1.248212509 0.001253

RHOB 2.750315282 0.001269

TMC5 -1.932938845 0.001271

PMF1 2.541748038 0.001284

ARF1 2.122071381 0.001287

IGLL1 1.966344711 0.001298

NMUR1 -1.050380476 0.001304

MRLC2 2.816083951 0.001309

PAGE5 -1.156743839 0.00131

METRNL -2.755459194 0.001319

ORC2L 2.97573762 0.001343

RNF5 -2.311268934 0.001352

NBPF3 1.942554268 0.001358

GRB14 -1.553907297 0.001365

THC2586764 2.561237286 0.00137

AQP10 -1.170320177 0.00137

YPEL4 -1.385158933 0.00138

NLK -2.50465345 0.001392

NARF 1.939521719 0.001397

RICTOR 1.981233181 0.0014

SMG6 3.783470683 0.0014

PACSIN1 -1.49418361 0.001409

CXCR4 2.052810241 0.001434

TGM3 2.274063865 0.001436

TBXA2R -1.42074201 0.001436

PPARG 12.12989029 0.001441

FAM13A1OS 1.925261479 0.001451

KLF14 -0.861598694 0.001454

MTMR1 2.756074931 0.00146

FAM40A 2.585454478 0.001463

AK021458 -3.432119162 0.001478

CX3CR1 -1.135189432 0.00148

TBC1D20 -1.652158065 0.001482

ACAA1 2.229250398 0.001482

KIAA2013 2.086197516 0.001483

TPM1 -1.666261698 0.001485

CTAGE6 3.436547695 0.00149

RNF19A 2.618394016 0.001492

C1orf91 2.649232913 0.00152

ITGB3 -1.511203194 0.001529

SRI -2.551461525 0.00153

SRRD -1.418149115 0.001546

MPP1 -1.312869104 0.001549

CUL5 -2.411128563 0.001577

THC2691419 1.410248207 0.00158

CREB5 1.464786908 0.001614

XR_015343 1.196260819 0.001615

C13orf16 4.781034788 0.001616

UNQ2550 -4.366615613 0.001625

DNAJA4 -1.570431192 0.001643

RBMS1 2.439921814 0.001646

C4orf38 2.102569222 0.001655

ANKHD1 3.423330703 0.001665

C1orf103 2.37049011 0.001671

STX3 1.75254669 0.001679

HIST1H3D 1.701691085 0.001686

PDE1C 12.56686032 0.00169

TMEM121 1.472722842 0.001691

HMGCR 2.381536547 0.001701

GFOD1 -1.354992281 0.001704

RBM38 -1.124862245 0.001705

ZNF394 2.954461394 0.001705

ORMDL3 -0.73931622 0.001705

GPR56 -1.02108379 0.00171

PIP4K2A -1.3415849 0.00171

ASXL2 2.674164504 0.001711

A_24_P632160 2.949774237 0.001711

C15orf52 -1.626148614 0.001719

AK095151 1.370451479 0.001734

LOC100130600 1.573262473 0.001739

LDHA 2.240528266 0.00174

WNK1 -1.111878152 0.001749

CENTD2 1.725586132 0.00176

THC2663970 1.186535127 0.001763

PRKCA 12.46406103 0.001768

CTNNAL1 -1.096982572 0.001791

SLC25A14 3.059152527 0.001793

BM932296 -2.833040805 0.0018

C3orf39 -1.895622244 0.0018

NEU2 59.58029645 0.001805

LYK5 2.384628461 0.001828

PF4V1 -0.594792939 0.001836

GBAP 1.850300249 0.001837

KLC1 2.456914666 0.001842

TFEB 1.992381737 0.001854

LOC286144 2.570125561 0.001858

HSDL2 1.569122261 0.00187

AK022044 -3.953366377 0.001882

DCP2 3.026677362 0.001883

S1PR5 -1.344967544 0.001891

PTGDS -0.997147298 0.001893

ZNF564 2.856002389 0.001911

RHPN1 1.961988562 0.001916

MIA 2.917595978 0.001921

UBE2D2 3.787847165 0.001924

MAN2A2 1.800874427 0.001927

ABCA1 1.386341045 0.00193

CA13 -1.727963818 0.001934

SPTLC1 2.947897549 0.001934

DAPK3 2.053337622 0.001941

YOD1 -1.408669556 0.001944

MED17 3.342010246 0.001948

KIAA0922 3.144348892 0.001957

COL13A1 -2.747932725 0.001968

DPEP2 1.684429332 0.001971

SPTB -1.512878237 0.001992

CNN2 1.139395937 0.001997

ANKRD32 2.402046261 0.002009

RPS6KA2 -1.511926612 0.002012

MEX3D -0.626267754 0.002019

FAM46C -0.975923366 0.002022

RNF24 1.305853053 0.002037

ZC3H7A 2.528898834 0.002039

FAM53C 1.689291071 0.002039

ROD1 2.079053224 0.002041

CCDC90A -2.197293157 0.002059

VPS8 2.472340854 0.002073

FOXH1 -0.851492393 0.002075

TAL1 -1.580588467 0.002076

S100P 0.892950433 0.002083

TRIM39 2.708979564 0.002086

TMEM185A -1.700641082 0.002096

PCNX 1.591034737 0.002109

TMEM11 2.888304043 0.00211

BTG2 2.406885037 0.00211

SIGLECP3 -1.07873873 0.00211

NFYC 2.877427326 0.002111

ATP6V0C -1.896707448 0.002121

THC2753543 -2.183401956 0.002136

CTNNBIP1 -2.163276644 0.002163

FBXO38 2.523878055 0.002173

IGF2BP3 -1.479979598 0.002207

UBN1 1.479076636 0.002215

PTPN4 -4.211901662 0.002216

TTC7B -1.300819269 0.002218

DHX8 2.257346024 0.002224

PIK3CD 1.55050036 0.002225

STK38L 2.388039628 0.002235

ZFP106 2.467003117 0.002241

USP48 2.630622597 0.002244

NUSAP1 -1.612271312 0.002253

TTF1 2.334763121 0.002259

HUWE1 2.592341182 0.002267

FOXJ3 3.037896107 0.002286

FNDC3A 1.969296253 0.002302

PHB2 -0.760199143 0.002332

THC2585049 -0.602890728 0.002341

ABCG2 -1.19536154 0.002343

A_24_P919063 -4.838827954 0.002348

SIRPG 2.180307784 0.002349

RPGR 1.802252924 0.002352

FAM152A 2.728376573 0.002353

NFE2L2 -6.611535761 0.002377

VCPIP1 2.54600628 0.002391

PDE2A -1.631414114 0.002391

BE542385 -2.849392278 0.002399

X57818 2.230957679 0.002416

CHMP1B 1.992882603 0.002418

C6orf21 -2.413769258 0.002426

CR617018 2.199882701 0.00244

GOLGA1 3.660658072 0.002445

HOMER2 -2.670655375 0.002447

GOLPH3 3.632205265 0.00245

RAB31 1.906480582 0.00245

LOC441131 1.471752911 0.002465

C14orf100 2.526602312 0.002467

UBXD1 -1.4444397 0.002467

C6orf25 -0.992648711 0.002484

LOC100129138 4.00807084 0.00254

GUCY1A3 -1.712404648 0.00254

KIAA0913 2.445244011 0.002545

TBC1D2B 2.520433347 0.002547

C9orf53 2.696046826 0.002569

TREML1 -1.25007384 0.002575

DCTN4 2.246366805 0.002581

H3F3B 1.866977454 0.002586

CLEC2B 1.676553336 0.00259

PLSCR3 -1.154726711 0.002592

LTBP1 -1.484876699 0.002593

JMJD1C 1.88463587 0.002607

TAOK2 -0.628573356 0.002608

FBXL3 1.998300964 0.002614

SP1 2.769794271 0.002624

BG185346 5.391178854 0.002634

A_32_P1434 1.986961756 0.002643

FBXL4 -2.296315606 0.00266

MRPL53 -1.642464386 0.002662

AF052115 7.52741417 0.002673

FADS2 3.623839505 0.002683

B2M 2.046589147 0.002699

EXOC1 2.310541611 0.002707

SLC7A6 4.547858314 0.002709

F13A1 -1.217251557 0.002713

AKR1C3 -1.261174645 0.002719

HIST1H2BM 1.586750729 0.002719

RAD21 2.398123936 0.002732

PYGL 1.289494227 0.002745

KRT18P49 3.563771159 0.002757

USP3 2.430778651 0.002758

BAZ2B 1.550975959 0.002777

IGFBP7 -1.212833106 0.002785

RFWD2 2.026639192 0.002788

HAVCR2 -1.477171067 0.002791

GNG11 -1.140152462 0.002791

CR609948 2.493806355 0.002793

TNFAIP8L2 2.047702874 0.002799

FER1L3 -1.010725434 0.002821

SH3BGRL2 -1.392561223 0.002822

LOC644538 -4.929023 0.002828

ARHGAP26 1.40811961 0.002834

A_24_P673209 1.143616397 0.002836

GSTM4 2.563997476 0.002837

VEZF1 2.658633507 0.00284

BASP1 1.154574009 0.002846

GUCY1B3 -2.000815215 0.002852

TRADD 2.895143678 0.002862

CSNK1A1 3.339154343 0.002877

ISYNA1 3.244946073 0.002878

GDNF 22.74559739 0.002879

PTGS2 1.497271212 0.002884

C19orf25 2.886143778 0.002884

LOC388564 2.01923064 0.002901

KIAA0753 2.751241439 0.002915

UNQ9438 -1.031730872 0.002916

C1orf128 -0.970128638 0.002923

MAP4K5 -2.234467982 0.002924

S100A12 0.816524355 0.002927

ADCK4 2.972519479 0.002937

C2orf25 2.135317563 0.002938

VCX3A 1.501222608 0.002942

PARD6A 3.142846904 0.002962

PCAF -1.311168769 0.002966

ENST00000249480 1.863091917 0.002971

VTI1B -1.377764979 0.002989

CR611712 2.234319981 0.002991

A_24_P204374 2.104818881 0.003004

NAT10 -0.772305536 0.003027

AK023533 2.788100736 0.003032

FERMT1 25.83119164 0.003033

RB1CC1 1.955844816 0.003036

WNT10A 2.134000554 0.003054

VPS37B 1.889882244 0.003055

SPOPL 1.990723962 0.003073

ZBTB44 -2.691480078 0.003073

PTEN 1.650804245 0.003094

LOC644950 1.749514601 0.003096

C18orf10 -0.996793279 0.003097

TMUB2 1.62097226 0.003097

LYNX1 -1.19920154 0.003106

ZNF775 2.598918062 0.003108

LOC728003 1.706819595 0.003124

ZNF767 2.494283364 0.003129

THC2628099 -0.581055092 0.003142

IYD -2.188276961 0.003147

EPB42 -0.832608924 0.003154

SLPI 0.883568516 0.003158

FUT11 -1.494976842 0.003165

FGFBP2 -0.870463235 0.003166

RIOK3 -1.144007364 0.003185

LOC728763 -2.258543025 0.003198

CD104030 1.395148769 0.003205

PSMD4 -1.712518542 0.003209

PSMC1 3.918327211 0.003221

SF3B1 2.42517042 0.003226

GDPD3 1.53893726 0.00323

ZC3H3 2.993560384 0.003237

SYAP1 -1.584759784 0.003241

E2F4 -1.524968015 0.003256

EIF1 -1.7893023 0.003259

CCDC66 -5.812445344 0.003267

CD38 -2.997161537 0.003288

GPR39 2.947068317 0.003291

CR596746 2.529343297 0.0033

C14orf119 2.7087286 0.003339

ADAMDEC1 7.528647146 0.003348

RPLP0 1.858997974 0.003353

ARPC1A 2.807061241 0.003355

BRMS1 1.995517826 0.003356

DarkCorner 13.23997079 0.003376

ZP1 4.835864485 0.00338

A_24_P641406 1.246099656 0.003382

THC2661968 1.578734576 0.003384

GOT2 2.393646101 0.00339

TFDP2 -3.425670838 0.003392

SPAG9 1.748802378 0.003404

PALLD -1.476503332 0.003411

A_24_P736617 1.879799765 0.003414

NYX -0.605457114 0.003419

AK054879 2.95071586 0.003427

ARID1A 2.510862874 0.003429

FPGT 2.380521588 0.00344

IRF2BP2 2.2182833 0.003486

CTTN -2.337687097 0.003488

CR617556 -1.758627217 0.003489

ZNF333 -0.819148811 0.003499

KCNQ3 -0.64281288 0.003507

TBX21 -1.029145391 0.003511

HIST1H2BI 1.615490593 0.003513

ZDHHC17 1.922369152 0.003522

C8orf55 -1.811839765 0.003538

UBE2H -1.377466529 0.00355

LTB 1.497991198 0.00355

DSC2 2.073032905 0.003563

CLIC4 -1.550303478 0.003566

S100A8 1.293368337 0.003581

A_32_P95502 -1.817974956 0.003582

ANKRD13D 1.327796139 0.003586

MUC1 3.500834159 0.003591

SLC39A4 -2.066909965 0.003607

MGC16384 1.68347894 0.003614

STAM2 3.005831045 0.003618

TRPM2 1.640249195 0.003624

ENTPD1 1.614482965 0.003625

CTAGE4 2.947052541 0.003628

AK093617 2.01152893 0.003631

ETG10_236652 7.125513441 0.003635

ETF1 1.948452404 0.003641

XR_018907 0.746262313 0.003646

TET2 1.817346612 0.003671

RP5-1022P6.2 1.86400167 0.003675

A_24_P323974 3.09946182 0.003679

PGBD3 2.887841768 0.003682

TMCC3 1.386619774 0.003685

ZNF518A 2.501363917 0.003707

LRMP 2.04285203 0.003709

POGZ 2.520081783 0.003717

CSDA -1.065688292 0.003731

EEPD1 1.868467394 0.003733

F2R -1.864200091 0.003739

GPR109B 1.181144912 0.003747

ENST00000250524 1.877440692 0.003752

MXD1 1.431068786 0.003755

HMBS -1.216652534 0.003775

LOC100128731 -2.424198 0.003796

APBB3 1.634016028 0.003807

FEM1A -1.362312821 0.003808

FAM127B -0.883311586 0.003838

ARHGDIA 2.568871549 0.003839

C14orf129 2.196933956 0.003897

CLTC 2.936800207 0.003905

SLC25A37 -0.880851305 0.003906

PABPC1L 2.280543021 0.003908

FNTB 1.791817908 0.003913

TMEM16F -1.336101844 0.003928

WIPF2 2.114532913 0.003947

USP8 2.545017556 0.003968

15-Sep 2.029610021 0.003978

GMFG 1.498702725 0.003986

ITPK1 1.545700187 0.003991

APOBEC3B 0.960686761 0.004002

CHPT1 -1.116551846 0.004018

MKRN1 -0.9982766 0.004025

APOBEC3G -1.36579855 0.004027

CCDC28A 2.173784514 0.004034

A_24_P367397 -1.823830482 0.004038

C14orf45 -1.069911661 0.00405

SH3TC2 -3.338794208 0.004054

XYLT1 2.878780702 0.004055

CD302 1.56257893 0.004063

SP110 1.514556619 0.004075

LOC391160 1.682950879 0.004089

MPL -1.116705258 0.00409

THC2682672 -2.702183802 0.004099

NRN1 -0.465424283 0.00411

DPM2 -0.986804604 0.00411

NFKB1 2.873154236 0.004118

GSTM1 3.702681838 0.00412

TINAGL1 2.63656428 0.004133

PRR5 -0.854533101 0.004158

TRIM27 1.441347289 0.004167

RPL13A 1.98125672 0.004173

A_24_P607107 1.97596324 0.004175

SSPO 2.720466771 0.004202

IL11RA 1.651854636 0.00421

ERGIC2 2.188700448 0.004215

LCP1 1.81462737 0.004225

DLX2 -0.596480585 0.004237

BTN3A2 2.025173801 0.004241

RCOR1 2.37473385 0.004242

GCLC -1.132663408 0.004245

W95720 12.70399999 0.004265

RCOR3 -2.286455172 0.00427

CCDC15 -3.1813473 0.004277

ALG6 2.05834139 0.00428

FUNDC2 -1.310968406 0.004285

TRIP6 1.990220625 0.004302

THC2752167 -0.559521803 0.004304

USP25 2.788550741 0.004316

PTP4A1 2.006285144 0.004318

ZMAT5 3.263959369 0.004319

FAM122A -1.459700953 0.004329

EXOC8 2.240237392 0.00433

UBC 1.998129009 0.004335

RCBTB2 2.047270041 0.004336

THC2676436 -1.700040495 0.004351

BC044624 -1.267173956 0.004355

THC2654231 -0.913383861 0.004358

TNFSF10 1.174961711 0.004371

RAC1 1.731364611 0.004385

CCDC17 1.629654431 0.004389

W60781 -2.792753107 0.004399

BX101927 3.214042138 0.004403

LENG8 -0.66789531 0.004429

TREM1 1.01821773 0.004431

AL050131 3.089880116 0.004431

PSAPL1 4.546365498 0.004434

THC2634909 3.35310059 0.004442

C1QTNF3 14.85546891 0.004446

RHOG 1.31387311 0.004449

LOXL2 -2.394295353 0.004452

IKBKB 3.081230949 0.004462

MEGF9 1.230327392 0.00448

PTGFRN 2.690372113 0.00448

SSBP3 -2.139596968 0.004482

RABL2A 1.822793703 0.004483

THC2670504 -1.241549141 0.004488

NLRC5 1.406964159 0.004489

NAAA -1.802028962 0.004498

COL10A1 -2.472247058 0.004517

MARCH8 -1.126802964 0.004517

LAPTM5 1.531032208 0.004522

GLRX5 -0.955842567 0.004529

A_24_P186354 -0.97437645 0.004539

GNAS -1.928630821 0.00455

SPTBN4 -0.96301781 0.004554

FAM152B -2.362396907 0.004565

CENTB1 2.840873021 0.004567

FBXO9 -1.072331884 0.004569

KLHL18 2.505499293 0.004584

CCPG1 1.841745989 0.00459

FAM44A 1.626990845 0.004598

FAM36A -2.013707149 0.004614

CREBL2 -1.53036462 0.004618

CHID1 -1.799374198 0.004621

VSIG4 1.020071601 0.004656

GNAO1 3.544532372 0.004657

ZSCAN16 2.775921672 0.004668

AB362555 -1.539225688 0.004674

A_24_P818378 13.90275588 0.004679

THC2754464 -0.500583691 0.004683

YIPF6 -1.25377247 0.004693

NCLN 2.310802223 0.004695

SERPINB1 1.371876214 0.004698

C17orf68 1.998430395 0.0047

CHRAC1 -3.430313925 0.004701

TCF3 -1.227373944 0.004711

HNRNPH3 1.983575501 0.00473

PACSIN2 1.375322486 0.00474

RESP18 8.640877413 0.004747

CDKN1A -1.434706808 0.004771

PAK2 1.629378512 0.004791

CA4 0.897124254 0.004792

RGS14 1.218423413 0.004795

SEC24D 2.540357115 0.004803

SLC25A23 3.425842685 0.004813

ARHGAP25 1.68164378 0.004819

MAP1S 2.149702351 0.004831

MTIF3 2.327879149 0.004832

TP53I3 1.724125012 0.004833

RELL2 2.309288961 0.004842

SERP2 -1.75939003 0.004868

HKDC1 2.473613639 0.004882

RRM2B 1.574020972 0.004916

RPS19BP1 1.886969516 0.004918

SECISBP2 -1.547259047 0.00492

A_23_P156609 1.466574042 0.004921

NUP98 1.758439207 0.004924

SRC -1.691522191 0.004941

ITGA2B -0.827274252 0.004956

NPC1L1 -0.654936431 0.004959

ZG16 12.16297375 0.004974

DNASE1L2 4.191322995 0.00499

PPP2R3B 1.498429726 0.004994

IFITM3 1.082895871 0.005003

VPS28 -3.088976972 0.005004

A_24_P307395 1.979075229 0.005014

RBMS2 -1.168170111 0.005028

THC2659392 8.873111122 0.005052

TUBA1C -1.085336507 0.005062

IGF2BP2 -2.206323687 0.005067

LRRC6 1.19245425 0.005084

FLOT2 1.004020451 0.00509

NBR1 2.477422116 0.00509

DPH3 1.87671834 0.005093

THC2557053 1.655469871 0.005097

HOXC9 20.63494346 0.005097

LOC100130339 1.345890933 0.005115

MED31 2.786877423 0.005122

THC2553569 -0.705736986 0.005126

FAM48A 2.397300479 0.005132

CMTM2 1.109406017 0.005146

PHLPP 2.545347917 0.005156

LOC144438 2.246745423 0.005157

SMR3A -0.595771619 0.005162

PPAN -0.778105413 0.005164

SGOL1 -4.612251261 0.005165

COL12A1 -2.198682815 0.005171

ELOVL7 -4.229774044 0.005179

KIAA0317 1.963325257 0.005182

C14orf139 -0.729063066 0.005188

RGS2 1.125925193 0.005213

HSP90B1 -1.39481098 0.005223

PLEKHA3 -0.648992408 0.005225

ZFYVE16 2.120540987 0.00526

ENST00000270031 1.139199577 0.005286

A_24_P534290 2.506050327 0.0053

PTMS -0.601743233 0.005308

HPS1 -1.58911787 0.00531

MMP25 0.928794585 0.005317

VWA3B -5.447661805 0.005322

XR_018818 1.93752333 0.005331

HCG18 -1.135811809 0.005335

NAT14 -0.773536956 0.005351

H2AFZ 2.047933368 0.005361

CDC14C -4.238461656 0.005361

CASP8AP2 2.402362989 0.005362

UBE2O -0.87560445 0.005369

SGK3 2.025729323 0.005384

CYB5R4 1.842370251 0.005392

FKBP1B -1.647276595 0.005421

USP49 6.311440844 0.005427

FBXO33 1.795728425 0.005462

ENST00000340536 0.925430685 0.005466

DYSF 1.042073275 0.005468

ANP32A 2.098635407 0.00547

SCFD2 -2.152156938 0.005484

HSD17B11 1.508743752 0.005491

ANK1 -0.792448528 0.005502

MARCH2 -0.861957677 0.005503

MKLN1 2.090257756 0.00551

SLC9A3 4.83576394 0.005519

DST -1.36912555 0.005547

MAP2K3 -1.061947472 0.00556

FGFR1OP2 -0.960848915 0.005567

DDX60L 0.970403206 0.00557

NPEPPS 1.859959179 0.00557

PYGM 2.81533192 0.0056

RBPJ 1.53323511 0.005606

SEZ6 -1.326992408 0.005625

LOC440295 2.327919074 0.00563

USP31 -3.474823458 0.005632

FLJ11151 1.610461314 0.005635

BP290435 -1.18767267 0.00564

RAPH1 -1.52076795 0.005645

SF1 2.287625552 0.005646

PLAU 1.848703947 0.005648

XR_019306 -1.125815148 0.005655

PAPOLG 2.022797305 0.005668

DYNLT1 1.844457692 0.005673

PCSK6 -2.406402574 0.005679

PSCDBP 2.086628096 0.005687

THC2733814 -1.024360057 0.005688

ZNF622 3.555428858 0.005704

ENTPD5 -3.222251493 0.005731

REN 5.242989371 0.005735

LSG1 -1.637540119 0.005757

BCL2L1 -0.927886352 0.005765

SPATA13 1.754458666 0.00577

GYPC -0.961275788 0.005787

THEM4 2.41820886 0.00579

A_32_P58464 2.573613795 0.005804

AB046850 -1.512004203 0.005823

XR_018299 7.452236703 0.005824

UCKL1 -0.606143278 0.005824

MS4A7 -0.872184583 0.005827

LOC204010 1.482019008 0.005828

ENST00000324414 2.716957726 0.005865

SELENBP1 -0.717748159 0.005891

ALAS1 1.99443641 0.005894

ARHGAP19 1.892224092 0.005908

CCDC117 -3.207124024 0.005911

A_24_P401150 -0.887454296 0.005915

MSL-1 1.51609343 0.005918

ENST00000397683 -1.378138015 0.005968

PBX1 -4.056068249 0.006009

AI064892 -0.563752509 0.006011

AL555100 -0.694924599 0.006015

BC030115 2.558239669 0.006022

EIF1AD 3.67767964 0.006058

NDFIP1 -2.61683948 0.006058

CCDC71 2.268081077 0.006067

MED15 1.506467719 0.006069

C3orf34 1.075517716 0.006109

KIRREL3 -0.637252204 0.006126

C1orf198 -1.250467713 0.006141

C11orf79 2.073393293 0.00615

SCRN1 -1.322033953 0.006153

CHADL -1.64749829 0.006156

PGF -0.642399056 0.006158

MLXIP 1.32483247 0.006162

KIAA1012 2.684339585 0.006181

PHF20 2.656447889 0.006196

ACSL4 1.366155974 0.006203

ADAR 1.556517391 0.00621

HHEX 1.807927018 0.006212

ANXA11 1.375015978 0.006213

IL8RA 0.972917674 0.006225

AW015165 3.928405355 0.006243

PHIP 2.379627519 0.006247

BANP 1.886642123 0.006247

AK096483 -1.69263284 0.006258

SEPX1 1.173122787 0.006258

SEC11A 2.401483557 0.006289

AF086125 -3.493173858 0.006312

C16orf70 3.110922921 0.006318

BC062780 1.22507709 0.006328

CR592318 3.501191578 0.006329

DGCR14 2.347011834 0.006345

HIST1H3F 1.596077423 0.006351

PGRMC1 -1.047745324 0.006378

OR10H2 -0.660288841 0.00639

DYRK3 -1.236685153 0.006395

SPEN 1.833972314 0.006402

NFXL1 -1.992188878 0.006412

MKKS 1.960503986 0.006413

BC070327 1.32391327 0.006418

IL10RB 1.520933649 0.006425

RPL17 1.408107911 0.006426

AK057787 4.045895226 0.006434

MAGEA2B 10.62802288 0.00644

BLVRB -0.96774873 0.006461

A_24_P409681 1.587209095 0.00648

RAF1 1.558337268 0.006532

TLE4 2.065143218 0.006534

MAP1LC3B 1.929315153 0.006557

MGC15705 11.74726833 0.006567

STAG3L1 -0.538779901 0.006603

U69195 1.611916712 0.006605

SNX9 -1.390387353 0.006606

BC039414 -4.992146496 0.006606

C6orf1 2.361636798 0.006624

APOB48R 1.602431923 0.006625

PPP1R9A -3.326195444 0.006644

A_24_P15083 1.685036143 0.006648

PDCL -2.121466462 0.006676

U09197 2.406567879 0.006677

S100A9 1.00142855 0.006677

RASL10A -3.102476213 0.00668

MYH11 3.676009953 0.006685

ECHDC2 1.676749 0.006696

PELO 1.793940479 0.006703

LYSMD3 1.881038422 0.006708

NFAM1 2.227373437 0.006714

A_24_P910169 1.576387304 0.006727

BC038512 1.274453534 0.006739

RRAD 1.948537198 0.006749

AQP3 1.039979576 0.006754

LOC652005 2.214000053 0.006755

KLHDC8A -1.46606243 0.006755

A_24_P915883 12.3292539 0.006756

AK131023 -2.718733547 0.006757

A_32_P75141 -1.966715336 0.006765

MLL4 1.311307265 0.006766

A_32_P26721 -2.518399028 0.006767

C20orf30 -0.742482102 0.00677

ZNF418 -0.607730533 0.006777

A_32_P5514 -1.109514625 0.006781

SCN3B -0.789439735 0.006781

BU674437 -0.627435221 0.006791

EVPL -0.726881961 0.0068

AL359650 -0.582044653 0.006804

AY007156 1.546132342 0.006822

SLC13A4 2.406704467 0.006842

DPPA5 -1.444832741 0.006845

CCDC128 2.450410494 0.006852

C22orf25 -1.380489493 0.006874

THC2679544 -2.355878965 0.006881

EGFL8 -0.681890084 0.006885

RAC2 2.28938975 0.006898

PIAS1 1.683118498 0.006898

FGD3 1.324456538 0.006899

MPZ 3.49518269 0.006904

DGAT2 0.790711618 0.006912

C19orf23 -0.779679578 0.006912

THC2504035 1.635492914 0.006925

S100A4 1.630671326 0.006938

HSAJ2425 1.635795346 0.00694

TMED5 1.469333263 0.006949

VAPB 2.729874751 0.006962

SLITRK2 1.157006412 0.006962

OSBPL5 -1.308248282 0.006969

CR600369 1.895089668 0.006978

HERC4 2.180470243 0.006982

RBM10 2.006494238 0.006993

THAP7 2.849673973 0.006994

ZFAND6 1.783126138 0.006997

LIMD2 2.174521568 0.006998

ECE1 1.204466161 0.007001

CYP26A1 -3.146712204 0.007008

TMEM115 2.307152598 0.007008

REXO2 -1.474617748 0.007018

ZBED1 1.407724656 0.007029

FIS1 -1.231495694 0.007031

C1orf86 1.980561985 0.007042

AL516445 -0.751835277 0.007043

HLA-DPA1 -1.073173679 0.007048

SIRPB1 1.626612001 0.007056

A_24_P24230 2.323063377 0.007056

BC045174 1.119257456 0.007073

ENST00000249062 1.966489734 0.007077

PRDM2 1.777378939 0.007111

THC2579654 -1.33984432 0.007111

THC2667995 -2.894956227 0.007118

KLHDC8B 1.619504898 0.007132

AW946823 -1.250744518 0.007136

SDPR -1.131765736 0.007144

ENST00000399573 0.942161647 0.007155

EPC1 1.089196397 0.007159

ZCCHC4 10.86299368 0.007168

IFT172 -2.23065912 0.00717

RILPL1 1.77844893 0.007174

A_32_P56726 -1.787408587 0.007196

TMCC1 1.474293897 0.007208

SLC11A1 0.658809099 0.007232

ZNF629 2.853474152 0.007249

BGN 2.214499871 0.007257

SETD1B 2.21054041 0.007257

RNF31 1.703594364 0.007266

PAPOLA 2.355384216 0.007267

CHIT1 2.004441739 0.007268

PLEKHO2 1.487027251 0.007286

ACAD10 2.537992528 0.007291

THC2567891 1.328457756 0.007313

SLC25A44 1.586608814 0.007333

RAD23A -0.930323201 0.00734

TUBA3D 1.188514215 0.007347

THC2688821 -0.512389855 0.007349

C16orf13 -0.724713475 0.007369

BQ364306 -1.168690326 0.007375

A_24_P323682 -0.784764516 0.007379

C5orf13 -1.130753269 0.007384

BE564275 -1.710726594 0.007392

BRD8 2.671797636 0.007401

ENST00000332666 8.733457204 0.007407

MET -4.331604233 0.007413

SLU7 1.966961609 0.007422

HDAC4 1.885108498 0.007436

A_32_P141849 14.97710605 0.007438

MGEA5 1.655335887 0.00744

GABARAPL3 1.700269597 0.007448

THC2548256 -0.610835588 0.007455

PIR -4.63360473 0.00746

SUMF1 1.73057846 0.00746

BI026064 0.73127322 0.007483

C19orf62 -1.673563927 0.007493

THC2603089 5.405566683 0.007504

UBFD1 -1.556283155 0.007513

FREQ -2.462718976 0.007528

THC2551912 -2.686260639 0.00753

C11orf54 2.140552776 0.007544

FLJ14213 -3.461408418 0.007547

REPS2 1.09130481 0.007551

EFHA1 2.294887489 0.007562

SP100 1.816540742 0.007594

AF070595 2.891170193 0.007599

IKZF5 1.71528338 0.007638

LRRC36 -3.173440063 0.007641

BC082970 0.917560702 0.007644

BAG5 2.616355032 0.007648

BI521983 0.940435827 0.007688

PYCARD 1.814943244 0.00771

KIF3B 2.317723817 0.007715

TXNL1 -1.785206975 0.007717

ENST00000285206 -1.469035083 0.007728

LOC340286 17.65657809 0.007741

ATAD2 -3.634446955 0.007742

COX5B 2.01974645 0.007747

A_24_P717824 7.827383236 0.007752

HNRNPK 0.991138972 0.007767

JMJD1B 2.255725747 0.007785

TMEM106C -1.335851387 0.007786

AURKAIP1 1.729910245 0.007787

SH3BP5L 0.849229237 0.00779

MYCT1 -3.43998663 0.007804

PCLO -0.630051424 0.007809

MSRB2 1.308193843 0.007811

CD6 -0.625779971 0.00782

SHOC2 1.796984806 0.007823

BIN3 -1.348132836 0.007825

RBBP6 0.615512991 0.007825

CA2 -1.090163195 0.007837

GPR142 -0.808272364 0.007845

GCA 1.009393567 0.007851

MICALCL -0.85510075 0.007867

PSPC1 2.235557435 0.007888

XR_018526 1.431455442 0.007892

QKI 1.211930868 0.007916

hCG_1984468 1.331109044 0.007917

STRAP -1.641558278 0.007925

BARHL2 1.803404118 0.007937

CR613944 1.287428465 0.007938

OR5AP2 -1.679398565 0.007945

GSPT1 -0.890222205 0.007955

A_23_P207049 -0.568932287 0.007958

CMAS -1.584465675 0.007958

TSPO 1.495715533 0.007964

HDAC5 -1.089649976 0.007985

CPA4 -0.968437027 0.007993

HIST3H2BB 1.710647345 0.007994

TBC1D15 1.888409721 0.007996

GRAMD1A 1.17442156 0.008011

BCL2L13 -1.680632611 0.00803

A_23_P213468 -1.081410772 0.008035

TUBB6 -0.843146176 0.008036

LMO4 1.484198099 0.008046

PPCDC 1.507283665 0.008048

MAPK13 1.238986662 0.00805

SPECC1 -1.962057691 0.008053

COPS3 -1.475369645 0.008062

THC2762463 -2.406989702 0.008063

SDCCAG8 2.48894714 0.008064

FHL5 -2.472430549 0.008071

TAL2 4.341521546 0.008086

KRT1 -0.461883494 0.008094

BC057844 -2.700952027 0.008096

MAP1B -91.67905956 0.008102

DYNLRB1 -2.570119642 0.008106

PLEK2 -0.855877088 0.00811

KLHL2 0.971574518 0.008123

MPZL2 2.449346736 0.008123

DQX1 6.56951793 0.00813

BGLAP 2.887075957 0.008143

KLRD1 -1.200558944 0.008145

THC2526432 -1.181623186 0.00816

UNKL 1.470692285 0.008176

MGC13379 2.376687245 0.008183

MMRN1 -2.194751641 0.008183

PTGDR -1.437491084 0.008197

MLSTD2 2.032275699 0.008206

AKAP4 10.40478017 0.008212

WDR32 -1.09648401 0.008223

CD242823 -0.774953179 0.008233

THC2625851 -0.516342307 0.008244

UBL4B -0.941129365 0.008247

SHD -0.696640279 0.008251

ATHL1 0.851066491 0.008258

IL6 -2.149306803 0.008273

C20orf43 1.803413841 0.008285

BF805854 12.10617714 0.00829

CR606969 -0.736587154 0.00829

TLR10 2.600844936 0.0083

ZNF655 1.881986205 0.00832

FAM131C -0.739569511 0.008327

WNT9A 3.212214495 0.008328

CDC2L1 1.530076489 0.008339

PCMTD1 -1.602528394 0.008357

CLK4 1.389112479 0.008364

AK022962 -1.359063387 0.008371

THC2636523 -1.428546952 0.008377

SNF1LK -1.378161108 0.008378

COX4I1 2.015586411 0.008403

A_24_P367063 1.024350413 0.008406

MCL1 0.992079414 0.008406

SIPA1L1 -0.603765938 0.008413

AW014022 -2.297624439 0.008414

PHF15 1.786934925 0.008419

AA420998 -0.542391117 0.008421

SLAMF1 1.959059495 0.008425

KIF13A 1.410789642 0.008467

AA203154 -2.531366782 0.008484

LOC283412 1.37057706 0.008488

LPAR2 0.933889191 0.00849

GSTO2 -9.177243366 0.008496

A_32_P93584 -1.152172019 0.008499

A_32_P86494 1.684820816 0.008501

ABHD1 -0.655086561 0.008517

SLC15A4 1.884037885 0.00855

AMMECR1 2.755291971 0.008554

THC2655417 1.821721301 0.008591

YAF2 2.561631605 0.008591

PDE8B -2.126964467 0.008593

SPRYD3 1.690879872 0.008594

DIP2B 1.465015686 0.008603

ABCC11 15.43356231 0.008614

AQP1 -0.906903223 0.008617

SPRR2D 3.71909782 0.008627

UBE2W 1.649312878 0.008629

APH1B 1.532795352 0.008632

RNASET2 1.274369521 0.008667

TRIM10 -3.082970706 0.008671

FLJ20160 -3.357150718 0.008677

GRIN2A -1.789404348 0.008683

FAM109B -0.596818003 0.008684

PAPD4 1.608731364 0.008697

ASCL2 -1.572451203 0.008698

XR_018311 1.721718938 0.008703

CSDC2 -1.044436559 0.008706

LIMS1 -1.186492755 0.008706

PRMT5 1.309178379 0.008728

MXI1 -0.908543685 0.008737

LPIN1 1.292121802 0.008758

CHST2 -1.056357714 0.008762

BE551634 -2.153878392 0.008765

PNO1 -0.85354101 0.008769

IRAK1 -0.812813785 0.008779

UQCC 2.972836887 0.008797

SDCBP 1.130018455 0.008798

TNF 2.993385717 0.008799

AIRE 2.561821018 0.008804

FLJ39743 -1.938952068 0.008808

THC2671299 -1.303877112 0.00881

THAP6 2.131836496 0.008821

XR_019446 -0.815322905 0.008822

POLR2K 1.449407673 0.008829

FAM81B -1.584248998 0.00883

MANSC1 1.097075292 0.008837

BC040412 -1.109201538 0.008843

FAM130A1 2.903290119 0.008849

LOC402573 2.472891803 0.008877

A_32_P213946 1.325899454 0.008879

BIK 2.349396097 0.008881

LIPA -1.00430867 0.008894

CD239565 -0.856836247 0.008909

BC065754 -0.752533612 0.00891

CISD2 -1.270910924 0.008914

CDK5RAP3 1.695095122 0.00892

C1GALT1C1 2.183728633 0.008921

CIB4 14.27258365 0.008926

RUNX1 -0.50431264 0.008928

RILP -1.031048117 0.008944

ASRGL1 -1.452738547 0.008964

PRF1 -0.825414556 0.008965

NONO -2.223200132 0.008965

FLJ22795 2.170870755 0.008973

IDS 2.226081845 0.008984

SLC2A8 -1.885007757 0.008988

KIAA1539 -1.244906596 0.00901

PSMF1 -1.151423058 0.009013

BCAP31 1.862616684 0.009023

COG1 2.514955341 0.009031

TTC35 2.536166521 0.00905

TCIRG1 1.476913253 0.009053

AP3B2 3.885821337 0.009062

A_32_P119949 2.229928331 0.009064

NP -1.649086992 0.009107

FBXO30 1.674006268 0.009109

AGPAT2 2.139309276 0.009115

OR1F2P -1.671032321 0.009118

PHKA2 2.0379576 0.009141

HAGH -0.95467583 0.009146

KIAA0319 1.68213612 0.009154

THC2777277 15.20762691 0.009157

ARMC7 1.829164492 0.009163

CBX7 1.724879743 0.009165

CD511705 -1.57527829 0.009198

HTATIP2 1.377276064 0.009202

SLC34A1 -0.791225278 0.009203

DMTF1 2.141069912 0.009225

THC2560357 -1.772971108 0.009227

CTF8 1.423911297 0.009231

LOC730394 1.119013991 0.009243

DDX3X 1.405055208 0.009245

C20orf141 -0.539602804 0.009251

C1orf66 2.777649765 0.00926

U92025 -4.198568346 0.009274

GPR124 2.319950767 0.009277

PAGE2 -0.944772607 0.009282

MEGF6 1.328499091 0.009289

TMPRSS4 -3.865595378 0.009293

A_24_P942954 4.447544902 0.009293

TWF1 1.909866241 0.009301

ZMYM6 1.997051581 0.009321

LOC401127 -0.91517919 0.009323

KIAA0196 2.411673972 0.009334

C21orf91 2.32352682 0.009357

MGC4473 -0.569380586 0.009363

AF010236 5.223236005 0.00937

A_32_P6062 -0.610021529 0.009382

C10orf109 -1.087407033 0.009384

IFNGR2 1.652824208 0.00939

WDR55 2.184152351 0.009391

LGALS3 -0.920194485 0.009392

MRAS -2.88978146 0.009392

OTUB1 1.604585768 0.009394

LINS1 2.175357865 0.009448

BAG1 -1.536601001 0.00947

ENST00000391373 -0.84466087 0.009472

PRKDC 1.369683669 0.009491

EPB41 -1.226027267 0.009493

SNORA70 1.699152433 0.009503

TNXB -0.986012709 0.009516

REEP1 -0.558376791 0.009517

NSUN4 -0.87004905 0.009519

MGC13057 -1.117663482 0.009524

LOC645195 -1.029691362 0.009532

TUBD1 2.919241065 0.009533

CD359823 1.210763342 0.009535

A_32_P222060 -1.32911992 0.009545

BC037919 -1.56868281 0.009565

HEXIM1 -1.340134681 0.009579

C9orf114 2.16974927 0.009587

AA465699 -0.541069759 0.009591

CPEB4 1.148574391 0.009594

UBE2D3 -1.265127085 0.009615

MAPK8IP3 -1.460025021 0.009623

SYDE2 -56.3367494 0.009653

TUBA4A 1.043911555 0.009659

IFRD2 -1.590510789 0.009691

AK055915 1.308041865 0.0097

A_24_P593724 2.289039526 0.009702

LRWD1 1.164528804 0.009707

BX089493 -0.692940473 0.009726

LOXL3 -1.693000011 0.009739

PNMA6A 2.435276968 0.00974

TBK1 2.042263034 0.009744

A_23_P151376 -2.080821341 0.009751

RAB24 1.364399146 0.009751

ARHGEF7 2.234758535 0.009758

SUCLG1 1.99205788 0.009761

PDLIM7 1.117275784 0.009772

IER5 -0.9856479 0.009804

BC002470 -1.202806872 0.009828

C10orf38 3.40599226 0.009833

THC2659236 0.877309237 0.009843

SLC7A14 14.12299808 0.009863

CAV2 -1.457092681 0.009882

PPP3CA 1.521740576 0.00991

ITFG3 -2.101758744 0.009912

ST7OT1 -1.857052052 0.009915

LRIT2 4.554652764 0.009932

RTN4RL1 -0.819906622 0.009936

UBAP1 -1.079794504 0.009941

TMEM59 2.1316812 0.009953

A_24_P307466 1.951086449 0.00996

RABGAP1L -1.192967117 0.009961

LOC442590 1.370833555 0.009974

ARL6IP6 1.949535563 0.009982

MAST4 2.822669179 0.009992

C7orf16 -1.025047929 0.010046

HRG -3.300062581 0.010058

RNF103 2.167005823 0.01007

NCR1 -1.191972503 0.010083

PGK1 2.291663097 0.010088

PLDN -1.254260065 0.010111

ARHGEF16 -0.71606589 0.010185

WBP11 2.10119986 0.010191

TMEM99 1.755446977 0.010203

DNAJB6 2.29148749 0.010218

MYLK -1.193481505 0.010225

A_24_P919256 -0.706910745 0.01024

KLF11 -3.230804493 0.010265

THC2539923 -0.616395564 0.010277

TMEM158 -0.770277347 0.010285

CYP1B1 0.886976341 0.01029

FOXO3 -0.900209944 0.01031

C10orf76 -0.647676444 0.010316

LOC730144 -1.770052993 0.010346

IL8RB 0.858300199 0.010353

PTGS1 -1.264756679 0.010355

APC2 -0.826020109 0.010377

GATA1 -0.936184517 0.010383

NFIA -1.162630878 0.010397

8-Sep 3.160715398 0.010414

C9orf25 -0.542465028 0.010429

ZFPL1 2.170555584 0.010431

RIMBP2 8.601166672 0.010439

A_24_P349756 1.340502843 0.010443

INTS10 2.32025452 0.010455

CD243286 -0.540872772 0.010465

SURF1 1.684369586 0.010475

VPS35 1.930775303 0.010492

SH3TC1 -1.575519701 0.010509

LOC388610 -3.699888722 0.010513

XR_018574 1.480444977 0.01052

CEACAM4 0.858575852 0.010527

EPS8 -1.788472076 0.010535

CECR7 1.34943705 0.010548

F5 0.993024737 0.010571

AZI1 1.616256909 0.010578

AV749851 3.180188975 0.010597

FUT7 1.06684808 0.010602

MED8 2.109725767 0.010603

KCTD19 -3.951790075 0.010606

YWHAB 1.705395529 0.010607

HBXIP 1.92801698 0.010611

THC2515402 3.887679565 0.010626

CDH8 11.67113209 0.010634

C19orf55 1.851473117 0.010656

DNAJB2 -1.208460223 0.010661

AF035790 4.834134834 0.010666

G6PD 1.181647417 0.010684

PRDM11 -0.828415471 0.010687

CASP5 0.845875552 0.010718

GGA1 1.875368794 0.010753

AHCTF1 1.302559651 0.010778

THC2753719 -0.582506797 0.010821

DEK 1.981702252 0.010855

AT_ssH_GD_5 5.95078024 0.010862

LEMD3 1.816756358 0.01087

B4GALT5 0.791060569 0.010877

MTMR11 -0.754006841 0.010878

BC028243 2.10582665 0.010908

LOC440731 1.269450496 0.010908

IL2RA 3.108814638 0.010922

CD3E 2.182267608 0.010929

KIAA0427 -0.849569003 0.010931

FAM117A -0.866686567 0.010941

PHLDB3 1.341661615 0.010958

LOC100131707 -1.167637311 0.010967

SLC25A38 -1.625872109 0.010988

GPR107 1.734501621 0.010991

AARSD1 -3.929843229 0.010993

ZFP36 1.602871061 0.010997

SLC24A3 -0.766866834 0.011007

DHRS1 -0.75407429 0.01101

PIP5K1A -7.33337155 0.011014

TM7SF2 -1.579706856 0.011026

CRIP2 1.81438811 0.011027

AI337612 -1.113610508 0.011028

TMEM40 -0.934924821 0.011039

LOC728450 -0.837143824 0.011048

TAF9B 2.662716609 0.011054

E2F1 -2.457729792 0.011057

GPR146 -0.916633713 0.011061

NHS 1.45043391 0.011062

THC2625585 -0.964048318 0.011069

BC039246 2.190849546 0.011099

IL2RB -0.976696629 0.011118

CHURC1 2.284498871 0.011132

A_32_P331700 -1.092654042 0.011134

THC2647213 2.455233278 0.011138

ANKS3 -0.787345724 0.011138

DPY30 2.253632869 0.011139

ZRANB2 1.516617107 0.011143

HBP1 1.530415793 0.011151

RHOBTB1 -1.316423611 0.011157

MGC24103 -1.238784778 0.011157

TMEM8 1.153942432 0.01116

CCM2 1.03103332 0.011164

TFPI -1.600143922 0.011168

GIPR -0.569003941 0.011168

SLC25A28 -1.289349073 0.011187

IGLL3 0.60858272 0.011201

AP4M1 2.277186347 0.01122

HGS 1.766195514 0.011225

CORT -0.508784834 0.011227

EREG -6.913811517 0.011241

PPAPDC3 -1.504012829 0.01125

KRT31 -0.576104332 0.011252

AF147727 1.974522778 0.011262

LOC441455 -0.842112729 0.011265

RCN3 1.119710395 0.011269

CR602022 1.936867093 0.011293

A2ML1 -1.132660062 0.011295

FAM134A -1.167979227 0.0113

HIST1H2BO 1.667430592 0.011306

IFI16 0.947679065 0.011321

CEP164 2.82611457 0.011322

AK023400 2.150152816 0.011351

ENST00000371081 -1.225293579 0.011356

THC2545097 -1.318680381 0.011399

A_24_P565503 1.405674372 0.011407

GYG1 1.017272589 0.011421

ZDHHC12 1.740555099 0.011425

NXPH3 2.238076139 0.011427

FTH1 -0.893575871 0.011433

WDFY3 1.230916766 0.011441

ZNF791 2.195415935 0.011453

FHL1 -2.142019676 0.011483

BM978707 -6.984495566 0.011484

PLCD1 2.085021773 0.011496

CAV1 -0.907662119 0.011502

PHF13 2.291007466 0.011524

LOC170082 -1.252145465 0.011539

SCHIP1 -2.633669583 0.011542

THC2588392 -0.504104806 0.01156

RABEP1 -0.702566372 0.011564

IFFO 1.958432526 0.01158

IGF2R 0.957103966 0.011587

NPDC1 1.253370038 0.011621

CTDSP2 1.246279196 0.011624

GTF2H1 1.856321743 0.01164

PPP2R5A 1.638941538 0.011641

ILDR1 0.833919967 0.011665

VPS4B 1.49608927 0.011677

ENST00000292543 1.170277622 0.011682

DNAL4 -0.524112578 0.011699

BX115782 3.315601199 0.011705

WDR40A -0.702321029 0.01171

HLA-DQA2 -0.418994974 0.011713

FAM104A -1.211245351 0.011729

IMPDH1 1.582231382 0.011731

PTAFR 1.11354686 0.011753

C19orf47 3.683495968 0.011774

PITPNM2 -1.173335834 0.011776

GIGYF1 -0.551174262 0.011808

GRAMD1C 1.101896236 0.011815

BX099483 16.80971331 0.011838

LPIN2 1.6821986 0.011841

SLC38A10 -0.849520721 0.011862

GATA4 5.711345946 0.01187

C20orf3 0.844172343 0.011886

SLC26A6 1.610156239 0.011899

LOC730286 0.930051458 0.011907

THC2537721 -1.210004317 0.011918

THC2569387 3.452716585 0.011921

DNM3 -1.259461725 0.011934

VPS24 -0.617670154 0.011939

BET3L -1.610462174 0.011958

PROS1 -1.166207611 0.011961

VSIG1 3.023794741 0.011974

TEGT 1.910665711 0.011979

UBE4A 1.919468891 0.011982

CNPY3 1.678486624 0.011986

ARHGEF4 1.309835052 0.012011

SMCHD1 1.266469215 0.012011

RANBP10 -1.226618145 0.012015

BX105952 -0.5537079 0.012019

FGF1 5.964603707 0.012021

SIRPA 1.382949346 0.012038

A_24_P281264 -1.232506313 0.012052

PEX5 3.553615145 0.012062

UBTD1 1.407284698 0.012064

A_32_P72887 -0.67041757 0.012067

LOC642076 1.606378414 0.012101

LIN9 -0.459637985 0.012105

WBSCR19 1.453804243 0.012109

ENST00000400767 -4.066222196 0.012109

PLAT 28.7137722 0.012125

TTLL5 2.599057408 0.012129

TRIM3 9.306942146 0.012142

CA420688 -2.646737615 0.012148

XR_018215 4.770825368 0.012151

KLF5 1.535754664 0.012166

BX537551 -0.486109465 0.012193

CHD8 1.613097163 0.012194

AK096031 2.24118485 0.012215

SPO11 18.12535862 0.012236

ALS2CR13 -1.244970052 0.012258

FAM45A 1.619241322 0.012268

EQC 4.767436346 0.012274

GGNBP2 2.01022647 0.012279

GPC4 -0.521822324 0.012279

XR_019095 2.209123566 0.012325

ISCA1 -0.850675566 0.012341

JAK3 -0.5277399 0.012341

A_23_P435390 0.641455177 0.012363

ABL2 2.843238372 0.012367

IL16 1.526183768 0.012369

FBXO7 -1.137918916 0.012369

MPHOSPH6 6.033727414 0.012377

ALOX12 -1.375888175 0.012405

A_32_P93807 -0.659705929 0.012421

AK091904 2.855797401 0.012428

SFRS18 1.571734281 0.012429

HIST1H3B 1.414111478 0.012442

CDH26 2.060222114 0.012444

STK4 1.28515538 0.01245

A_24_P924125 -1.045796854 0.012454

AW076051 2.001618811 0.01246

AK021432 -1.241798562 0.012461

PDZRN3 2.228327366 0.012467

C9orf156 1.851418863 0.012468

LOC388381 4.341417621 0.012487

ETG10_195139 3.572596552 0.012487

EIF5 -1.729009913 0.012488

AK314822 3.621498801 0.012489

A_24_P755505 1.313417971 0.012494

PCDHB13 -0.713210858 0.012502

A_24_P213396 -1.816000285 0.01251

CTSE -1.279361168 0.012523

C16orf35 -0.826019112 0.012569

VILL 1.872679406 0.012581

TNPO1 1.944708597 0.012586

YIPF4 1.84121422 0.012627

FBXO31 -1.327669439 0.012641

PTK6 2.724531739 0.012642

HLA-A 1.016399767 0.012643

APOM -2.587685841 0.012648

RPL3L -0.519902798 0.012655

CLEC4D 0.59416604 0.01269

PIP4K2C -1.983761374 0.012708

BF149382 -0.542801917 0.012711

AT_nD_3 3.591188034 0.012718

LOC339344 2.252866679 0.012725

CCR2 1.103789081 0.012728

PICK1 2.883474844 0.01275

LILRB1 -1.181196254 0.012753

BZRAP1 -1.20259785 0.01276

USP51 -1.957668383 0.01277

A_32_P10643 -2.933335499 0.012814

C9orf66 2.155159947 0.012832

A_23_P21862 -0.494688841 0.012842

T15787 -1.130319559 0.01285

KIAA0319L 0.924345436 0.01285

NEK7 1.579583958 0.012892

VCX2 1.45552028 0.012894

NUDT4 -0.574406322 0.012915

C1orf83 3.395470934 0.01292

ZNF75 2.180422164 0.012957

LPGAT1 1.7510023 0.012957

OBFC2A 1.379880005 0.012961

ELOVL6 -3.739979927 0.012985

PANK3 -2.47160262 0.012988

THC2651958 -4.303300065 0.01301

MATN4 1.907797852 0.013018

THBS1 -2.654440299 0.013035

SSTR2 -0.813324955 0.013041

ASPM -2.850458379 0.013048

ELF4 3.012942313 0.013082

X01147 1.329909753 0.013094

BG259864 -1.193640582 0.013102

FASLG -1.298251659 0.013105

HOPX -0.951531962 0.013145

CTSB -1.097891853 0.013159

RAB5A -1.18284876 0.013162

PUS7 -0.501157088 0.013165

IQCC -3.137830232 0.013206

LRRC47 2.025662352 0.013215

E1A_r60_a104 2.507454863 0.013223

VWCE -0.682663179 0.013226

AF305819 4.175282333 0.013232

GBF1 2.557215618 0.013238

AK5 -0.867335377 0.013241

CCNJL 0.965223971 0.013247

CABC1 1.087063457 0.013277

COPB2 2.152613093 0.013298

PRR11 1.119752486 0.013302

THC2558776 -0.532902034 0.013318

GPR132 -1.199650936 0.013319

UBA52 -1.52822491 0.01333

THC2734788 -0.89994137 0.013337

GAL3ST1 -3.265313293 0.013345

THC2525955 1.709489052 0.013348

RPS3A 1.319777789 0.013351

THC2653459 3.122361282 0.013355

BM351936 -0.779107436 0.013355

BC020909 10.9687747 0.013357

CCNL2 1.681024353 0.013359

ZBTB48 1.431090445 0.01337

CYP3A43 -8.16141006 0.013403

XR_015583 -0.869941757 0.013423

XR_018187 0.926689189 0.013427

hCG_20426 -0.372193759 0.013431

SPPL2A 1.543704782 0.013442

LOC100129424 1.261433177 0.013456

TMED8 0.975141593 0.013472

NR1D1 2.748262003 0.013476

MYH10 -2.259309331 0.013487

EXOSC1 1.597062993 0.013497

ZNF222 1.841829304 0.013499

ENST00000306515 -0.867533114 0.013502

PSEN2 2.488509594 0.013514

HAVCR1 -4.951937489 0.013517

AP1M2 -1.47252847 0.013519

ALPL 0.734439841 0.01352

GGTA1 -0.673894245 0.013536

SAA2 2.939585377 0.013545

ADIPOQ 5.170595594 0.013546

KCNJ15 0.838982049 0.013549

ENST00000325769 15.51833408 0.013554

TPM3 2.58951932 0.013565

AI732974 -0.772848059 0.013573

KDELC1 7.836887236 0.013588

RPL3 1.259753447 0.013591

CYB5B -1.36155004 0.013603

AK027610 1.625356494 0.013606

C3orf63 2.174833677 0.013614

BU568037 -0.868385033 0.013618

PPP4R1 1.368259724 0.013655

RNPC3 -0.619708698 0.013664

RGS12 -1.126787691 0.013678

CYHR1 1.556489377 0.013712

SLC4A1 -0.871994334 0.01374

RORC -0.88731522 0.013774

STARD10 1.42604593 0.013804

P2RY1 -2.061330883 0.013824

RTKN2 1.128124124 0.013839

B9D2 1.383838651 0.013844

PGD 0.934643011 0.013849

TRIM2 1.579286433 0.013862

DSCR3 2.098982064 0.013865

UBE2V1 -1.350659403 0.013897

ALOXE3 -0.957022732 0.01399

HS1BP3 1.46282075 0.013997

KBTBD7 1.191202499 0.014001

SPATA2L -0.62725279 0.014013

AFTPH 1.984319735 0.014016

CR595483 8.500382449 0.014032

FCER1G -0.629429308 0.014045

BMX 0.829096897 0.014046

LRRC37A3 6.306666051 0.014049

TNS1 -0.633445805 0.01407

KCNN4 1.003190443 0.014072

ZNF581 2.057082812 0.014091

TSPAN18 1.673787011 0.014097

CYP4F22 -2.453427357 0.014099

THC2690528 -0.520322923 0.014105

IFRD1 1.232888243 0.014131

KIAA1211 -3.733958378 0.014137

CALML4 -0.670455305 0.014139

CGN 3.555231909 0.014143

PTK2 -3.330272048 0.014143

A_32_P91902 1.526008266 0.014147

C11orf72 -0.530225324 0.014176

CACNG5 -0.66136207 0.014198

SART1 1.419403441 0.014199

MOV10L1 -0.981502631 0.014206

KCNG1 -0.817942186 0.014212

RNF151 1.11906543 0.014216

AATF 1.682467535 0.014243

GDAP2 2.188731138 0.014243

A_24_P525144 -1.138144168 0.014247

CDH9 -3.676812364 0.014251

LALBA -0.526577173 0.014252

HSPC159 -0.811088018 0.014253

SMCR7L 2.424841685 0.01426

TP53INP1 1.245705933 0.014276

BG284526 -1.530881176 0.014281

GPR155 1.363722551 0.014285

SPI1 1.307448638 0.014293

STK19 2.633277369 0.014294

LAMA1 24.86348426 0.014321

C20orf29 -1.763897365 0.014338

BSPRY -2.932250603 0.014346

AI359141 1.713999985 0.01435

KCNS1 4.379208929 0.014351

PRPS1 -0.929536415 0.01436

ODC1 -1.096303112 0.014361

TP53TG3 -2.219110779 0.014364

UNC13D 1.21731869 0.014365

RPL29P2 1.349505639 0.014368

C1orf25 2.206762556 0.014371

LOC100129858 2.330144525 0.014387

DGKA 1.445828577 0.014393

MSN 1.773498635 0.014403

RASD1 -0.95658879 0.014422

ENST00000354668 -0.564987302 0.014434

TSEN54 -1.131351078 0.014453

PAPSS2 -2.339378558 0.014466

SGSM3 1.744428316 0.014476

RAPGEF2 1.514811065 0.014478

RBBP5 3.518258011 0.014481

HIST2H2AB 3.47222973 0.014522

RAB40AL 2.393355232 0.014523

LOC100131650 2.129422661 0.014532

EZH1 1.398047752 0.014541

RSPH1 -1.761890363 0.014563

MS4A6A 1.204340716 0.014601

PRDX2 -0.745286392 0.014601

TAS2R8 12.80420389 0.014603

HNF4A 4.124674441 0.014611

ELF2 1.014320999 0.014614

AMFR -1.395919953 0.014628

ST13 -1.874355846 0.014628

BPIL1 -0.393216219 0.014646

VASH1 -0.714719345 0.014649

FAM38B -3.019093003 0.014649

ANKRD9 -0.94021412 0.014652

PAFAH1B1 1.56456299 0.014652

GSK3B 1.870404271 0.01467

THC2659529 14.37953105 0.014698

ARPC4 2.29730817 0.014703

TRIM47 2.102347305 0.014723

LOC642826 -1.14374765 0.014731

LFNG -0.76383832 0.014747

MCART1 -2.104217377 0.01475

GPR42 -0.573486131 0.014758

C5orf32 0.937210557 0.014809

ADAMTSL1 1.98937251 0.014812

RPL10 1.174961334 0.01483

UGT2A1 -0.614114091 0.014833

BC033528 -0.577638537 0.014871

LSM14A 2.306914232 0.014874

XR_016161 2.170188527 0.014895

tcag7.1015 1.198882438 0.014915

RP11-529I10.4 -0.906869223 0.014917

CCDC93 2.149892553 0.014921

THC2632823 1.194119879 0.014922

KRTAP4-11 -0.637431162 0.014934

GAS2L1 -0.971655692 0.014938

MMP1 -1.733781424 0.014941

C17orf72 -0.601900581 0.014969

KRT86 -2.127738049 0.014998

FLJ37078 -0.629391586 0.015013

AK026194 1.208945708 0.015015

JMJD6 1.655116726 0.015017

BAIAP3 0.930604902 0.015024

X05126 1.130088379 0.015027

A_24_P918364 14.28045092 0.015029

A_24_P703642 -1.20178266 0.015031

MMAB 2.540012579 0.015034

TUBB1 -1.236883653 0.015036

STX1B 4.945387543 0.015038

CDC14B -1.221452529 0.015041

A_24_P928250 -0.360422754 0.015057

KRTAP8-1 0.958165776 0.015071

PIM1 -0.914786291 0.015123

MYOZ3 -0.801810767 0.015126

INTS12 2.566397056 0.015135

C7orf41 -3.370843022 0.015141

MAPRE1 1.968129003 0.015142

SCAP 1.165568397 0.015148

C16orf86 -0.791169955 0.015152

ZBTB11 1.599276234 0.015153

THC2679040 -0.499807366 0.015171

TMEM123 1.111131623 0.015188

ARF4 2.10681927 0.015193

FAM120B 2.114841057 0.015199

FBLN5 1.185514533 0.015203

A_32_P189324 -2.115059883 0.015216

HIST1H2BL 1.697307644 0.015221

CAPRIN1 2.191276971 0.015228

EFCAB3 -0.613468524 0.015232

IGHA1 0.44207162 0.015265

CAMKK1 0.76384307 0.015278

ABHD11 -1.765490233 0.015289

CTSC 1.90946639 0.015293

BC061642 2.143923276 0.015303

CR596670 2.10721189 0.015305

XR_018524 1.620873427 0.015314

RAB27A 1.421859203 0.015328

HIST1H2BH 1.50796022 0.01534

STAB1 0.780684052 0.015343

WDR90 2.412562391 0.015347

AK000336 1.772597714 0.015357

MGAT3 1.113439502 0.015369

BAX 0.653919101 0.015369

UBXD8 2.406735607 0.015376

GPC1 1.370159621 0.015386

ZFAND5 1.735904227 0.01539

DDEF2 -1.11442953 0.015402

MAP3K2 1.240093185 0.015408

LOC339123 1.841132291 0.015421

CHRM1 -1.173368126 0.015458

THC2550160 -5.458831219 0.015474

AXL -1.382727082 0.015477

TFDP1 -2.33896067 0.015481

DIABLO 2.217369684 0.015486

SCN10A 13.52386962 0.01549

PNPT1 0.941449059 0.015516

ZNF419 1.967686443 0.015529

CR608275 1.22224303 0.015536

TAF4 1.408078699 0.015538

DOK3 1.498788465 0.015547

LOC653458 2.109414859 0.015564

NSUN5B 1.277144343 0.015571

COL23A1 2.591804368 0.015589

CSAD 1.359904048 0.01559

CCDC9 2.24323353 0.0156

TXNDC13 1.2498369 0.015601

THC2671169 2.756367652 0.015614

FAHD1 -1.458555738 0.015615

ACTN3 1.336436815 0.01564

AVIL 1.398460389 0.015646

SCML4 1.58561499 0.015659

ALS2CR12 -4.682641489 0.015659

PHF23 2.046971605 0.015698

ITGB5 -0.92754429 0.01572

THC2597357 1.015945027 0.015723

AA132288 1.269842451 0.015726

HIST1H2BC 1.561439242 0.015738

ENST00000379864 -0.606157692 0.015745

C19orf59 0.667822209 0.015758

CC2D1B 1.607440223 0.015759

SLC39A7 1.656238896 0.015775

SETD2 2.007158502 0.015789

NPHP3 -0.977871939 0.01579

HES4 -0.571373713 0.015791

THC2637028 0.887481485 0.015796

SON 3.215878287 0.015798

USPL1 1.788439845 0.0158

GNAZ -0.757763204 0.015814

POLD4 -1.48084984 0.015832

A_24_P845631 17.32098836 0.015833

LDLR -0.947634727 0.015837

KRAS 1.521311786 0.015844

GLYATL1 -2.725702617 0.015867

ENST00000330710 -1.319974156 0.015875

ULK1 1.174352978 0.015878

BPGM -0.63277624 0.015879

KIAA1797 -0.778091488 0.015883

THBS3 1.695919104 0.015891

SH3KBP1 2.131355827 0.015917

TXNDC12 1.412877001 0.015918

FXR1 3.560568916 0.015927

AF074982 16.62042696 0.015929

SLC31A1 -2.105571223 0.015941

THC2718727 2.111312562 0.015949

AK124256 -6.662021689 0.015982

RPL32P3 2.046548701 0.016012

SIAH2 -0.796594705 0.016012

GIGYF2 -2.128487189 0.016017

NPM1 -1.1934044 0.016028

C14orf152 -0.548684449 0.01604

C17orf45 -1.105673102 0.016064

RASGEF1A 1.744325188 0.016065

ENST00000283760 -1.377289398 0.016078

ITIH5L -7.641293886 0.016079

RASSF5 1.66036272 0.016081

TMEM183B -1.521513752 0.016092

ACSBG1 -1.470232096 0.016093

C8orf60 0.995943032 0.016099

ABCD1 1.987198717 0.016099

PAGE2B -0.994572928 0.016104

THC2700145 -1.251271277 0.016104

ZDHHC14 -1.537844179 0.016117

CCDC59 2.620208073 0.016134

FLJ42709 18.07643631 0.016162

CIRBP 0.959164225 0.016177

CEP68 1.799349386 0.016181

A_32_P55438 0.857418361 0.016184

XR_018155 1.344476045 0.016203

MFSD5 2.158365751 0.016213

C1orf9 1.686585889 0.016225

GLT8D1 1.832452874 0.016225

MDH2 2.374393925 0.016245

A_32_P35839 4.542432045 0.016249

CSNK1D 1.050700183 0.016262

MXD3 1.047326509 0.016269

CAMTA1 3.943653979 0.016279

LOC388494 -1.104181819 0.016298

CLU -0.773016374 0.016302

BC063022 -3.854639682 0.016304

EIF2AK2 1.183585597 0.016314

PTPN18 -0.940180583 0.016325

LINCR -2.049418847 0.016332

MFSD4 -4.082062936 0.016344

A_24_P160920 1.30581819 0.016349

TMEM63A 1.075312348 0.016355

FOSL1 3.496293522 0.016377

NSMCE1 1.88963318 0.016388

MRPL45 -1.37233375 0.016409

THC2649401 -0.604124577 0.016411

LOC145758 2.423121046 0.016416

TNFAIP6 0.666365526 0.016418

RAB14 2.457484256 0.016419

A_24_P340886 0.806590108 0.016432

ENST00000399198 1.257883093 0.016439

MICAL2 -0.967895622 0.016445

SEC14L4 -0.865447966 0.016445

CNOT8 1.82652098 0.01645

ZNF193 2.111796696 0.016466

UBA1 1.165196223 0.016472

MTERF 1.840537603 0.016489

PRPF38B 2.205614944 0.016489

SLC35A1 1.423417055 0.016502

SELK -1.140308022 0.016507

EFHD1 -1.91295423 0.016514

ENST00000383267 1.502511335 0.016519

AY172962 0.515949434 0.016533

MLLT11 -1.121601179 0.01654

DHX16 2.184049217 0.016552

DIS3 1.280605381 0.016596

IRAK4 1.396935658 0.016601

A_24_P20623 -3.930943205 0.016603

XR_018040 -1.117571906 0.01661

CCBP2 2.781407428 0.016636

KRT18P42 1.484653911 0.016664

THC2522223 -0.77199839 0.016674

A_24_P650011 1.40498296 0.016696

ENST00000211092 -0.779223273 0.0167

MAN1A1 -0.988521087 0.016729

WBSCR18 -1.840215755 0.016732

HMOX2 1.170994734 0.016734

MTERFD3 1.604057468 0.016784

C9orf165 -1.066578159 0.016801

PA2G4 -1.320088509 0.016809

A_24_P144275 1.11750064 0.01683

BAT5 1.833591614 0.016837

CRABP1 -0.543708541 0.016847

SOS2 1.337901361 0.016872

PDLIM2 1.148761928 0.016885

PCGF3 2.131870642 0.01689

BQ233868 -1.802052216 0.016913

CEP63 1.371266463 0.016927

AK058000 -3.840929275 0.016931

CNOT2 1.358200005 0.016964

MAX -1.619637068 0.016987

JARID1A 2.068444768 0.016992

THC2643233 1.581910291 0.017

RP3-402G11.5 1.330805122 0.017011

A_24_P565496 1.224721889 0.017011

C14orf145 2.966470663 0.017029

VIT -2.813880665 0.01704

RAB36 0.647919652 0.017052

FNBP1 1.971613463 0.01707

TEAD3 -0.541172511 0.017081

CENTA1 1.510115251 0.017087

THC2613527 -0.84182469 0.017088

C11orf51 1.849150203 0.017095

NAG 1.745484021 0.017103

ARSF -3.431757819 0.017115

CSNK2A2 2.666288529 0.017123

ADNP 1.829971787 0.017128

MAD2L1BP 1.900794596 0.017132

C12orf31 2.793529863 0.017149

SH3GLB1 1.178548023 0.017164

NCOA7 1.454080366 0.017204

EEF1G 1.200097651 0.01726

C17orf80 1.656982576 0.017269

SETDB1 1.570704533 0.017287

TCEB2 -1.484293427 0.017335

LOC389833 -0.64782809 0.017343

NDUFC1 -1.240115704 0.017352

ERP29 1.180077338 0.017365

DOK4 1.411751069 0.017388

LOC200261 18.13314634 0.017412

PDZD3 2.216895587 0.017438

CACNB3 -0.520035956 0.017441

LOC401357 0.871590932 0.017457

ELMO3 1.920848975 0.017476

ZWILCH -2.466703843 0.017478

DALRD3 1.524805749 0.017487

C6orf173 -1.750604736 0.017498

THC2538873 94.68167335 0.017511

TRIM58 -0.657979393 0.017514

3-Mar -1.763957981 0.017521

PRO1848 21.84077039 0.017533

CD96 1.68081088 0.017544

CCDC47 0.919859823 0.017568

SGTA -1.148985512 0.017573

GGT6 1.909393461 0.017585

SNTB1 -1.096662473 0.017595

CD518305 -1.665149546 0.017613

LONRF2 9.310354937 0.017639

AK129584 -1.996904863 0.017655

ENST00000290390 2.43165321 0.017663

RAB13 -3.450096546 0.017666

BC040655 1.80939714 0.017687

EXOC3 1.288932456 0.017696

C13orf18 1.19133699 0.017702

ENST00000390541 1.457696004 0.017721

AK001784 -1.282820169 0.017721

THC2730708 -0.694527876 0.017741

CASP3 1.619974488 0.017752

ABCA2 1.840701637 0.017759

SPRN 2.288372826 0.017765

A_24_P850336 1.803193111 0.017773

CRYBA4 -1.169777478 0.017777

THC2604598 -4.939152935 0.017778

DPM1 1.813990172 0.017778

A_24_P367139 1.237967426 0.017804

KIAA0265 -2.064928763 0.01781

A_32_P116088 0.903443382 0.017811

BNC2 -3.479001887 0.017822

A_24_P178784 1.191450143 0.017825

METAP1 1.659456673 0.017855

AGPAT6 1.337516154 0.017875

A_24_P170147 1.057463633 0.017882

A_32_P6452 1.781512712 0.017886

SAPS2 2.17050736 0.017896

SYPL1 1.359158985 0.017904

BF932142 1.792947364 0.01791

LEUTX 18.67537753 0.017933

ENST00000333156 -1.018598601 0.017941

TRIP12 -1.262185648 0.017971

CRYZ 1.196108738 0.017978

OAZ1 -2.239673353 0.01798

BF446608 2.472792492 0.017982

CPT1A -2.993263664 0.018007

PFDN6 0.626148442 0.018027

VAT1 1.823570472 0.018043

ZNF692 1.678727348 0.01807

CTNNB1 -0.58421045 0.018087

NKG7 -0.913476039 0.018094

LOC440311 1.212341986 0.018103

ATP9A -0.999433543 0.018149

LOC100127980 -0.602641892 0.018226

AK025056 1.673024155 0.01824

DCT -0.509513704 0.018241

LOC151300 3.976186944 0.018283

TPTE2 12.9349972 0.01829

RAB3GAP2 2.106338346 0.018292

A_24_P358406 2.311074787 0.0183

THC2786662 -0.944730864 0.018309

THC2689970 6.535112551 0.018317

A_32_P108592 -1.444510939 0.01833

FANCA -1.076680589 0.018335

DOHH -1.250793058 0.018356

MAP7D1 1.027838213 0.018361

BF213738 1.157764999 0.018369

BM702130 2.317077162 0.018409

KNDC1 -2.718048209 0.018422

ALKBH2 -2.425828703 0.018439

KCNMB1 -1.140352015 0.018439

MAF1 -0.889154951 0.018451

PDZD11 -0.633892757 0.018459

BC065737 1.486822519 0.018473

KCNK15 -0.568820887 0.018477

CACNG7 2.961741454 0.018495

GPR97 0.650459917 0.018498

MRPS10 2.323490481 0.018511

OMA1 1.584808078 0.018512

B3GAT1 -0.865360601 0.018514

HARBI1 1.370969659 0.018516

HAL 0.873199143 0.018523

LMAN2 1.484993562 0.01854

FLJ40113 1.166178676 0.018553

FLJ31356 2.01426215 0.018569

NFAT5 1.270211894 0.018589

ZFP36L1 1.389064482 0.018595

CP110 1.880031634 0.0186

EPHA7 25.19786722 0.018601

MALT1 1.81877657 0.018614

C5orf22 -2.170567619 0.01865

KPNA6 -1.91609481 0.018652

XR_018420 1.987980339 0.018654

CR608907 -1.217777395 0.018656

RPS7 1.304392536 0.018671

SHE -4.821213597 0.018678

BY796363 -0.875580774 0.018706

TLR4 0.911196182 0.018736

TMEM62 2.838342557 0.018737

THC2669092 -0.502765921 0.01878

SLC2A5 1.671840159 0.018784

A_32_P188841 -0.82626957 0.018792

THC2714978 -0.412576542 0.018809

BC009800 -0.877550822 0.018811

ASZ1 10.85835514 0.018812

TBX4 3.738082617 0.018825

LOC150763 0.765361701 0.018846

TLK2 2.348883689 0.018852

TTC27 1.548975451 0.018876

C1QTNF6 1.515588465 0.018878

LOC100130175 1.728610124 0.018886

EIF4G1 -0.713832305 0.018909

A_24_P933538 -2.362653615 0.018934

LONRF1 1.38916381 0.018946

C6orf70 1.809720806 0.018967

TDG 1.984835717 0.018969

RETSAT 2.046360659 0.018987

KLC3 0.955929626 0.018999

TTC17 1.94769935 0.019001

ENST00000342314 -1.022731636 0.019006

MUC4 -0.514775917 0.01901

BQ003493 9.485325803 0.019023

RUNDC2C 0.940391609 0.019024

ALOX12P2 3.487956512 0.019027

BE218249 -2.391516874 0.019049

DTX2 2.159782395 0.019058

HECA 1.688757622 0.019096

NCOA4 -1.299946537 0.019118

TMEM56 -3.044005311 0.019148

C1orf21 -1.424412424 0.019161

MATN2 -37.36567956 0.019162

THC2583762 -1.044427676 0.019168

C17orf92 0.721136711 0.019178

THC2672675 1.397524249 0.019189

AL137705 -0.459895342 0.019191

RNGTT 1.663247882 0.019199

A_32_P151470 -1.057959369 0.019199

FLJ22763 6.757186301 0.019201

ZNF580 1.490897079 0.019218

AK074162 1.200242073 0.019249

MEF2D -0.504951666 0.019249

DCTN3 2.593731181 0.01927

RASSF4 -0.859934984 0.019286

C17orf65 -0.469548117 0.01931

A_24_P934541 4.009217626 0.019416

PLCZ1 -80.3715332 0.019426

A_24_P934861 1.684872397 0.019437

MME 0.677542202 0.01945

KIR3DX1 5.253232175 0.019497

NUPL1 1.349884418 0.019516

C1orf127 3.960636905 0.019527

ZNF335 1.411867895 0.019547

RYBP 0.864646136 0.019556

CLK1 1.250294569 0.019561

A_32_P78488 2.846218106 0.019601

SUPT4H1 1.336797065 0.019602

NGFRAP1 -0.99200487 0.019617

LOC100144602 7.474917443 0.019643

E01827 -1.271763667 0.019656

VCX 1.450900666 0.019663

WSB2 -0.984463719 0.019666

CA441889 -2.620295564 0.019679

BNIP2 1.451086128 0.019679

H3F3A 1.146758681 0.019682

RCC1 -0.508694543 0.019683

ATP11B 1.255035938 0.019687

AXIN2 1.311403968 0.019696

ANKRD13A 1.61227742 0.019721

ERCC2 -0.497523399 0.01973

LOC342994 1.259604831 0.01973

XRCC4 1.542986626 0.019741

BC090920 -0.958025453 0.019745

SPR 2.488059801 0.019748

SLC22A17 -0.749747646 0.019781

AA975908 4.263418643 0.019788

EML2 1.221606041 0.019805

A_24_P213144 1.643086366 0.019823

WTAP 2.295901597 0.019834

GPRASP2 -3.666378768 0.019838

LOC401010 3.03227007 0.019845

RC8 15.12577418 0.01987

A_24_P375340 1.306561828 0.019898

DNAH8 12.18631436 0.019903

SPINK1 -3.537981353 0.019906

PLCXD2 -0.384900059 0.01991

DNHD1 -1.559454486 0.019915

LRRC14 2.097308678 0.019956

THC2539706 -1.599153425 0.019957

MGC34800 -0.470415564 0.01996

FLJ20489 -0.946072411 0.019961

ATP1B1 -1.153512102 0.02

THC2657593 0.846307838 0.020011

XR_019070 -1.175067845 0.020032

BM808621 -0.466191917 0.020039

DEF8 -1.707686265 0.020047

KIAA1586 -2.061317216 0.020058

C14orf153 -1.762340339 0.020066

EVI2B 0.882445082 0.020072

RPSA 1.152075792 0.020093

C20orf149 -0.887757201 0.020101

GFRA2 2.350413462 0.020102

TP53I11 2.780232716 0.020142

MANEA 1.581343643 0.020149

C21orf89 -0.521264517 0.020152

PMS2L3 -1.605376932 0.020157

GGT1 1.459455403 0.020181

C21orf58 -0.3899899 0.02021

MAF -1.176712741 0.020223

CPT2 1.365789738 0.020226

CD244 -0.524039126 0.020241

C10orf118 1.712075773 0.020243

GALNS 1.278994161 0.02027

CCDC78 1.957150397 0.02028

SNAP91 9.691576499 0.020287

ECSIT -1.266532662 0.020302

KIAA0040 2.286293406 0.020359

GAK 1.167954545 0.020365

DCTN1 -0.406562472 0.020376

PRRT2 1.555854317 0.020376

FAM20B -1.186357265 0.020382

AI401434 9.303400464 0.020391

XR_018808 1.085352832 0.020419

ARL4A -1.166529943 0.020427

ARMC8 -1.359257672 0.020481

RGPD1 0.988942446 0.020486

TTC15 1.454186108 0.020486

BC063570 -2.183025652 0.020509

THNSL2 1.720115881 0.020534

FKBPL 4.655299168 0.020545

THC2532711 -1.034084941 0.02055

ENST00000391665 2.062466932 0.02056

PORCN -0.584377895 0.020567

A_24_P931583 -0.572969337 0.020599

C1orf217 1.608646986 0.020609

FAM103A1 -0.519328361 0.020628

EIF2AK1 -0.806934877 0.020667

MTHFR -1.028445088 0.020673

THC2557351 3.342481143 0.020675

DDX3Y 1.397499895 0.020704

WDR66 3.141681395 0.020705

C1orf34 -4.947327891 0.020711

NGEF 1.92434343 0.020728

A_32_P167723 -1.76361789 0.020731

UPK3B -0.564938968 0.020732

PLEK 1.79355743 0.020736

AK090694 -2.391818911 0.020756

OTUD5 1.783596388 0.020766

ZNF395 2.475088598 0.020773

DDIT4L -6.921252914 0.020803

ADIPOR2 -3.083585428 0.020833

SH2D5 2.28160802 0.020838

SYNJ1 2.859797816 0.020845

A_32_P202587 2.333787718 0.020863

ZNF271 -1.199412694 0.020871

TSPAN13 -1.471048701 0.020895

IL13RA1 0.988382931 0.020899

RALGDS 0.815394459 0.0209

ENST00000339692 -0.762051237 0.020906

C1GALT1 1.57989339 0.020916

DRAM 1.297287738 0.020986

INTS6 1.609040832 0.021002

GLT25D2 -1.527641664 0.021016

B4GALNT1 -0.543381012 0.021041

LOC390876 1.417928768 0.021063

AF130069 -2.08334162 0.021068

BC037911 -0.307491489 0.021071

AIM1 1.306196794 0.021095

FLJ22222 -1.069324175 0.021107

SMOX -0.848631117 0.021129

LOC92973 7.867178221 0.021134

A_24_P673119 22.14098134 0.021134

THC2529969 -0.487218864 0.021137

PIN1L 2.08524798 0.021145

KIAA1333 14.35404667 0.021192

BQ045216 26.6232303 0.021203

YPEL1 -1.42738458 0.021271

SDCBP2 2.265289331 0.021303

HABP2 23.81390559 0.021306

A_32_P58029 1.311030042 0.02131

PDE5A -1.307756365 0.021312

N4BP2 -1.089591017 0.021325

DBP -0.356544931 0.021371

CMTM6 1.130104032 0.021386

PLEKHM1 -0.592689851 0.021394

ALPPL2 -0.564563536 0.021404

A_24_P650893 1.142263264 0.021414

C1orf77 2.174765967 0.021434

AL137400 1.589544332 0.021443

AK090481 0.886213774 0.021443

CLIC2 -2.405521819 0.021469

SSR1 1.688238378 0.021487

PPP1R1A 4.494651636 0.021489

TRIM7 -1.497779513 0.021511

AF086286 -2.157461198 0.021515

PPP2R5B -0.82404849 0.021539

THC2645586 1.668620135 0.021546

TNFSF8 -4.207463154 0.021548

EG328448 -1.064652282 0.021552

A_24_P937931 13.11644063 0.021568

XR_018393 1.03950689 0.02157

EFNB1 -1.15983227 0.02157

ORAI1 0.989114651 0.021572

ARHGAP4 1.871251537 0.021579

FCGR3A 0.805786425 0.021583

SFTPG -1.334797194 0.021601

ALS2CR2 -0.700234022 0.021616

UNC5C -5.900923813 0.021617

GNPDA1 2.935552547 0.021665

MAGEA6 -0.926371871 0.021687

ELP2 1.627145413 0.021707

KCNK7 -0.475727444 0.02171

RAB5C -1.531440677 0.02172

MIA2 2.611934665 0.021726

FAM132B -3.372684785 0.021728

ZNF364 -1.682301357 0.021729

THC2699069 -0.498905053 0.021731

PLD3 1.3696228 0.021744

C15orf17 1.773200291 0.021769

WBSCR22 2.318609552 0.021828

ATOH7 -0.619262027 0.021833

IL32 0.797364896 0.021842

ARHGAP15 1.306018848 0.021845

FOXC2 -0.467271814 0.021848

ATP1B3 -1.024424924 0.02185

THC2534106 -1.361608453 0.021859

VEPH1 -3.205539759 0.021865

NP186315 -0.543353988 0.02187

LOC389634 0.837728558 0.021886

RDBP -1.807023325 0.021887

A_24_P919727 -0.547119077 0.021898

GATA2 -1.195918158 0.021912

EIF2B4 2.447379903 0.021933

AF161342 1.229103152 0.021945

A_23_P211603 0.770898423 0.021947

SDK2 -0.353502775 0.021953

A_24_P127082 1.090380917 0.021957

AK025116 -6.340665585 0.021968

GP6 -1.88736419 0.021994

BM681332 17.05948997 0.021999

LIPH -3.361070582 0.022004

KIAA0174 1.72794787 0.022013

RPS4X 1.118536481 0.022042

XR_018532 5.193292114 0.02205

JAM2 -1.735252404 0.022052

A_32_P178339 2.386304919 0.022074

PRKCZ 1.419859376 0.022077

NOVA2 -0.413531423 0.022111

GTF2B 1.137119369 0.022112

IL8RBP 0.678240789 0.022113

ENST00000332902 3.674930171 0.022116

A_23_P78975 0.639909205 0.022123

PMS2 1.969036782 0.022144

BX427435 -0.475692956 0.022172

LOC284244 -9.923175465 0.022174

E2F6 2.098208425 0.022177

SBNO2 1.02056778 0.022202

RPL34 1.351446732 0.02223

LOC100128548 0.654825175 0.022247

RNF13 1.089836956 0.02225

HISPPD2A 3.480534306 0.022266

RAB18 1.537484003 0.022268

A_24_P101960 1.326740532 0.022269

SSU72 1.587056171 0.022276

LOC645427 -1.047668197 0.022279

C9orf70 2.927718262 0.022287

TALDO1 1.438948486 0.0223

DKFZp761E198 1.366402292 0.022305

AK311609 2.273752875 0.022309

A_24_P755069 -1.356360297 0.022339

OMG 4.170848487 0.02234

TRIM41 -1.062556248 0.022347

CTAGE5 1.873577712 0.02235

THC2591088 1.700846507 0.022382

POMZP3 -0.541232482 0.022421

RPL36 1.195929216 0.022432

LOC283152 1.142690097 0.022445

A_24_P409420 2.960070372 0.022461

N4BP3 1.347992285 0.022465

A_24_P836315 -1.395077347 0.02247

AV756170 -1.349178267 0.022517

AK090466 -0.557180649 0.022535

ARF5 1.279572702 0.022536

ZNF2 4.012036046 0.022566

THC2690780 1.142655369 0.022585

AIFM2 -0.568614229 0.022592

THC2687538 -0.750604608 0.022593

GBA 1.221299444 0.022594

SNIP -1.879893438 0.022598

MGAT4A 1.975302158 0.022609

HERC6 0.86738187 0.022614

ABHD7 -4.930011598 0.022615

A_32_P218707 -3.081508107 0.022664

LOC440335 5.465438239 0.022671

A_24_P725365 2.614737442 0.022683

EPN3 12.80341667 0.022693

A_24_P6850 2.032517389 0.022702

SUGT1 -1.376894022 0.022729

LL22NC03-5H6.5 -1.083510371 0.022729

C12orf36 -2.958524068 0.02273

RHPN2 25.75424255 0.022733

PUM1 1.459422642 0.022755

BU679017 -2.148656465 0.022765

ATP5D 1.066170155 0.022777

MMP28 -0.744515293 0.022789

BZW1 1.588593587 0.022795

AP4B1 1.886657375 0.022798

SNX12 2.470609337 0.022802

PAPPA 58.20703244 0.022805

FLJ31713 3.494336147 0.022808

RENBP 2.103305182 0.022815

FLJ21272 1.148016056 0.022816

FAM149B1 -1.107055355 0.022818

RUFY1 -0.97097449 0.022818

ARSD -0.556218814 0.022853

PARP4 1.30627716 0.022856

LOC54492 -4.253020088 0.022857

NTN2L -0.766179484 0.022874

AGPAT3 -2.762985319 0.022888

LOC390595 -1.397668837 0.022894

XR_018980 -0.77942778 0.022895

CNTN2 11.81112037 0.022912

EIF2C2 -1.086438345 0.022921

EIF3EIP 1.346950165 0.022925

SLC40A1 1.217811011 0.022925

AI915259 -0.399192779 0.022943

RBM18 1.869575888 0.022948

ZER1 -0.931997304 0.02297

CD177 0.825391221 0.022976

THC2670307 12.98793777 0.023001

MBOAT7 0.85579742 0.023017

TCOF1 -0.711285634 0.023089

A_23_P136961 -0.461256943 0.023093

NPEPL1 -1.471528237 0.023113

THC2719547 1.593752226 0.023169

ENST00000332686 0.995238888 0.02317

PHC3 2.319142764 0.023192

FOXD2 -1.68474825 0.023201

IDH1 1.835182413 0.023204

HLA-DRB6 1.321856808 0.023204

KIR2DL4 -0.490614684 0.023205

ZNF616 2.000258769 0.023206

LOC728904 16.40422813 0.023231

CKLF 1.250598706 0.023237

NDUFA6 -1.423206335 0.023239

ARHGAP24 0.965787551 0.023302

IQSEC1 -0.928376647 0.023304

ZNF146 -0.876742661 0.023357

A_24_P204574 0.880453628 0.023366

GPR180 -1.897914167 0.023373

BC009366 8.370185162 0.023375

BX091687 12.15499066 0.023387

GNLY -0.534173829 0.02342

FNDC7 8.889533045 0.02343

P2RX3 -0.603448862 0.023442

CR604826 21.28830938 0.023444

XR_018572 0.893104937 0.023454

CCDC49 1.681968984 0.023466

LOC343515 1.043685766 0.023492

AFF1 -0.735101025 0.023497

THC2493066 2.352854014 0.023506

CHRM4 -4.308753935 0.023532

KIAA1549 -8.273518902 0.023536

RND3 35.68692642 0.023545

C22orf13 -1.000885841 0.023552

LMBRD1 1.450132649 0.02361

THC2534144 -1.687015706 0.023615

THAP3 2.000955233 0.023626

RUNDC2B -0.591722803 0.023636

MICALL1 2.020218616 0.023644

PTHR1 -6.06055332 0.023652

THC2729895 6.699126157 0.02366

GOLGA2L1 2.574938088 0.023669

RNH1 1.615373411 0.023673

THC2747684 9.624145471 0.023676

ALG3 -0.715749855 0.02368

LOC100128116 2.146189214 0.023681

AW043836 12.1962468 0.023695

FLJ43080 10.23343873 0.023702

IGF1 -1.409893288 0.02371

MAP4K4 1.42545662 0.023728

ATF7IP 1.830588403 0.023751

SMARCD1 2.365298707 0.023753

TMUB1 1.469756175 0.023778

ANKS1A 1.560898821 0.023784

A_24_P164815 -1.339935767 0.023799

LOC283177 -0.678972205 0.023808

SLC35A2 2.21592109 0.023828

BM479077 2.908883994 0.023859

EDF1 1.32968085 0.023885

C13orf15 -0.930615867 0.023885

THC2539425 -2.371751721 0.023909

dJ222E13.2 -0.514350728 0.023917

SLC9A8 0.98288088 0.023919

BQ017638 -1.506224217 0.023922

TNNT3 1.55734719 0.023948

CD2BP2 1.861914265 0.023956

TAS2R4 -5.650810344 0.023956

BBS10 1.322066836 0.023974

CEP350 1.848259844 0.023983

A_23_P257881 8.974221376 0.023992

ANKH -2.169452917 0.024003

LTB4R 1.046238171 0.024006

AK023077 1.876543261 0.024017

C10orf46 1.668626534 0.024026

LYZL6 -3.882131265 0.024031

PHF3 1.890916845 0.024032

AK094726 9.265324158 0.024033

XR_019546 1.00787985 0.024045

ZNF821 1.704198097 0.024066

RGPD5 1.474856199 0.024078

TIAM1 -0.565808049 0.024079

FGD1 2.53871282 0.024081

TMEM38B -3.992672117 0.024099

PTP4A3 1.193975733 0.024115

AF076205 1.131616459 0.024141

PRTFDC1 -1.999912245 0.024158

THC2537367 -1.851978426 0.024181

GPR125 -1.610604594 0.024202

A_24_P289865 -0.894286811 0.024213

BICD2 1.687556829 0.024215

RARG -0.731000325 0.02422

THC2704459 -0.857543237 0.024232

A_24_P916853 1.603311375 0.024236

THC2656784 -1.064004579 0.024249

ZNF618 -2.680703462 0.024262

INSL3 1.585936531 0.024287

ASCC3L1 1.284248793 0.024289

IQWD1 -0.930334488 0.024319

AK024147 1.191359501 0.02433

DYNC1LI1 1.394884191 0.024344

ENST00000324808 1.583492115 0.024345

EFCAB7 2.23505082 0.024362

LOC646090 9.12977214 0.02437

RAMP1 -1.004819286 0.024372

ZNF322A 2.176600231 0.024373

A_24_P350126 1.446034873 0.024374

SNTA1 -0.929243744 0.024397

HEG1 -1.203934799 0.024398

SLC12A6 1.45088633 0.024407

THC2571921 2.258059665 0.024407

PHKA1 4.315829023 0.024426

A_23_P5626 1.214061389 0.024426

NPSR1 -1.186431117 0.02444

A_24_P324538 7.802679473 0.024455

GLT1D1 0.932969213 0.024472

CXorf36 3.587358447 0.024492

BC041636 1.63507215 0.024498

IL18BP 2.435946732 0.024513

HBD -0.526184786 0.024515

ADAMTSL3 19.56135181 0.024569

A_32_P200820 -0.541808957 0.024599

LAIR2 -0.593326863 0.024602

PRIMA1 -0.500361978 0.024615

LOC165186 -0.836285616 0.024643

CYP2D6 1.532102153 0.024657

A_24_P144383 1.548940142 0.024675

SLC25A25 -0.828603608 0.024691

THC2631229 1.203563122 0.024734

ERG -0.809871722 0.024736

BFSP2 3.465378193 0.024745

KIAA0090 -4.775099495 0.024753

D2HGDH -0.594107314 0.024774

TCL6 -0.414550191 0.024779

AK026477 12.23319662 0.024784

THOC4 -0.749162457 0.024799

HCCS -1.690130003 0.024799

HP1BP3 -0.984564174 0.0248

A_23_P2032 1.699368004 0.024809

ASPHD1 -0.64127093 0.024809

REXO1 2.474275487 0.024818

TLR5 0.9657594 0.024819

CENTB5 1.977356046 0.024822

SYNPO 2.202873002 0.024839

CD53 1.451135166 0.024849

NDEL1 1.122759457 0.024885

IKBKE 1.292675173 0.024894

MSI2 -1.131541393 0.024895

A_24_P7470 -0.693300756 0.024923

PDRG1 1.709641829 0.024923

LSS -1.920834589 0.024931

LRRC46 3.406900748 0.024953

C18orf21 1.803334262 0.024975

MUSK -0.762404529 0.024983

KBTBD2 1.473572117 0.024992

TBC1D14 1.036089714 0.025029

SEC23IP 1.51458853 0.025037

PPY2 2.449283241 0.025038

DENND2A -0.768218832 0.025049

A_32_P128097 1.526424547 0.025059

BC036431 -0.415947479 0.025088

FLJ22536 -2.155929971 0.025117

SLC16A11 -0.563126571 0.025117

TBL1X 1.23782415 0.025134

NUDT10 -4.617488819 0.025142

GEMIN8 2.484954763 0.025194

LOC729768 1.961177978 0.025195

LOC390282 -0.851081025 0.025204

ZCCHC17 -1.088865293 0.025217

C18orf26 81.65682243 0.02526

FLJ42875 -0.88957432 0.025261

A_24_P367199 0.976580042 0.025265

CADPS2 13.71615533 0.025268

LEPROTL1 1.377274609 0.025272

RNF167 2.230509672 0.025303

ASXL1 1.505954776 0.025314

CTDSPL -0.991314542 0.025329

LOC339240 1.537153124 0.02533

KIAA1881 1.052869307 0.025337

RAB1A 1.855938069 0.025342

THC2706921 -1.668367299 0.025347

tcag7.907 0.873524249 0.025382

NRAS -1.960599353 0.025385

THC2667190 2.520032371 0.025388

STX18 -1.390507488 0.025398

STX10 1.317382167 0.025408

TCL1B 1.27141151 0.025416

C7orf34 1.14700935 0.02544

AK022252 1.85871732 0.025447

TTLL3 -0.588546569 0.025454

KCNK3 4.659619178 0.02546

AF116618 -1.338688717 0.025474

BM666433 -1.769533696 0.025556

SEPHS2 1.681580059 0.02556

PMAIP1 1.362914474 0.025569

BU537444 -0.784645125 0.025583

ASPH 0.86529926 0.025591

FNDC3B 1.413839499 0.025594

LOC283089 -9.629688504 0.025602

THC2668853 -0.605358084 0.025607

CD27 -0.811141923 0.025621

ERGIC1 0.749243473 0.025623

THC2778545 2.288617618 0.025631

LOC100129113 -0.472016342 0.025643

C1orf63 1.471674402 0.025675

SIDT2 -1.087514715 0.025677

THC2562425 11.75853653 0.025679

THC2541781 2.571318919 0.025696

LOC494558 -0.456371742 0.025699

KLHL22 1.575512308 0.025706

KRTAP4-12 -0.594968797 0.025708

A_24_P919265 11.69070329 0.025711

BC019667 -2.293149812 0.025715

THC2701422 1.392924161 0.025746

C2orf14 21.52342789 0.025752

SLC20A1 -1.004237534 0.025796

TSC22D4 1.137733533 0.025799

AK092378 -1.754268672 0.025802

AMBP 3.081235267 0.025811

THC2752010 13.19688272 0.025818

BCLAF1 1.265273154 0.025836

UBL4A -0.412235883 0.025838

A_32_P142195 7.804333528 0.025842

BC010544 1.174910579 0.025843

FFAR1 1.429961791 0.025858

ZNF688 -2.06214225 0.025865

A_24_P370484 -2.331668769 0.025904

A_24_P7040 0.787285838 0.025904

THC2657554 2.891607436 0.025947

ZNF18 2.195310505 0.025962

MAGED4B 1.406398647 0.02599

ATXN7 1.186872413 0.026009

GABRQ 2.499644994 0.026026

NP111770 0.863701104 0.026027

F12 -0.667365345 0.026058

OPALIN 4.119735064 0.026067

LOC644192 5.586700956 0.026069

GCC2 1.463019068 0.026102

CD93 1.012720811 0.026108

THC2544911 1.153979749 0.026121

A_32_P8857 1.443004612 0.026133

SERINC3 1.71612948 0.026137

XR_017079 -1.035546223 0.026143

BAT1 1.531226731 0.026187

AP1G2 1.27437751 0.026197

ACTL7A -0.8246923 0.026202

B3GALTL -1.70050866 0.026218

LETM2 2.670054169 0.026219

SR140 1.561676099 0.026221

PELI1 0.997834344 0.026222

PSCD4 1.16064524 0.026225

PSMD12 1.957762433 0.026249

FLOT1 0.856859833 0.026262

KIAA0888 -1.426370509 0.026286

TACR1 -0.885383745 0.026326

PRRT1 -0.7627066 0.026332

THC2689623 1.115427017 0.02634

KLHDC7A -0.551642428 0.026344

AW467174 3.023910052 0.026356

THADA 1.763180005 0.02637

AF319957 -1.0884476 0.026382

X75962 1.921916005 0.026386

GPR119 -0.493203715 0.026388

KRTCAP3 1.70796777 0.0264

MYH13 3.106426117 0.02642

BX101405 21.23641184 0.026463

PGM2L1 -1.067123876 0.02647

KRT18P33 1.476682412 0.026481

THC2557134 -0.683825124 0.02649

AT_P_M 14.39149073 0.026493

ENO3 1.601901155 0.026493

A_24_P502660 -0.939436821 0.026514

ZHX2 1.615488546 0.026516

A_24_P943411 6.487565355 0.026526

THC2719525 -2.996753014 0.026539

ZFYVE9 11.91100906 0.026547

CDA 0.69790935 0.02657

MMP19 -0.573229831 0.026572

NHP2L1 1.648353477 0.026591

IQCH 11.82287833 0.026592

ACTR1A 1.081865162 0.026605

SLC5A6 1.817192714 0.026627

S100A11 0.874073298 0.026676

C1orf118 -2.080558447 0.02669

EHBP1L1 0.879237617 0.026706

C12orf10 -1.082007913 0.026721

A_24_P922647 13.14288026 0.026724

ZNF20 2.662717953 0.026782

MEIS3P1 -1.148118489 0.02681

LOC392787 5.14776848 0.026833

THC2651395 1.631199313 0.026865

A_24_P919502 -0.4410575 0.026881

AL517609 -1.443735326 0.026884

FBXW2 1.937393237 0.026894

BX114329 5.353786398 0.02693

MESP1 0.977882324 0.026934

SLC37A3 0.819985965 0.026935

CDRT4 7.580590566 0.026952

MTMR9 1.957838685 0.026976

PHKG1 2.768778472 0.027006

WDR25 1.688438619 0.027025

LCMT1 1.626027367 0.027041

ZIC5 -0.698285551 0.027057

THC2609092 -1.6207506 0.027089

HNRNPH2 1.542116626 0.027108

KIAA1600 1.18912426 0.027125

TRIM65 3.011749417 0.027125

IFT52 -3.068160846 0.027127

SLK 1.480593435 0.027153

A1BG -1.444590945 0.027154

AF318328 1.423887174 0.027165

GPD1 1.459393385 0.027187

ENST00000343959 2.987205111 0.027196

ZFYVE20 1.455961729 0.027197

BG682198 5.257212473 0.027226

SEMA5A 2.439401126 0.027229

NRBF2 1.129804918 0.027235

A_24_P499481 -0.748224748 0.027238

A_24_P49421 1.152849283 0.027243

FOXL1 -0.550185084 0.027252

RASSF7 1.952809941 0.027274

BX088949 11.66005712 0.027282

RPL9 0.907565781 0.02732

BF692226 15.68786565 0.027321

CUL4B 1.837883083 0.027332

TNNC2 -0.933820523 0.027353

UHMK1 -2.174513043 0.027386

MPP6 -2.617413774 0.027416

C14orf113 1.440813766 0.027425

PAQR3 4.202313717 0.027432

CTSL1 -0.944223798 0.027437

HDAC7 1.112985373 0.027449

ELFN1 -0.404948596 0.02746

THC2721409 0.954520047 0.027467

RNASE7 2.509938388 0.027478

USH1C 2.011230719 0.027503

BC017563 2.389451305 0.027506

MX2 0.808574046 0.027523

FAM120A 1.321353954 0.027532

OR5V1 -0.83662778 0.02755

ATG10 -2.702896847 0.027555

SLBP 1.234599606 0.027557

LRRK2 1.049259782 0.027571

SMAP2 0.727428736 0.027575

A_32_P174385 -0.480548646 0.027596

RHBDL2 14.25004018 0.027627

NFX1 2.0338532 0.027649

A_24_P929867 2.312574421 0.027665

A_24_P25040 -0.632297632 0.02768

XKR5 12.07493118 0.02773

RCBTB1 1.449580881 0.027731

ENST00000324928 1.410165128 0.027741

PKIB 2.294323217 0.027743

ELF1 1.049543157 0.027744

MGC21881 -3.026968092 0.027747

SP8 -3.303236585 0.027761

KHSRP -2.250673308 0.027767

A_24_P93111 1.529642383 0.027773

A_24_P666795 0.753055982 0.027775

MAGEC2 10.64435971 0.027788

LOC727835 0.84954406 0.027799

COMMD7 -1.302943103 0.027848

OSBPL3 -12.2936739 0.02785

APC 3.194036366 0.027858

NOV -1.389572925 0.027863

HMX2 -0.444896937 0.027877

ALPK1 1.005919272 0.027879

NUDT11 -5.316817356 0.027885

THC2619545 -1.707343267 0.0279

THC2530075 -2.221234514 0.027909

RUNDC3A -0.702276046 0.027924

A_23_P206741 -0.533412164 0.02793

LOC137886 0.973256627 0.027956

MDM2 4.211882423 0.027956

RGPD2 0.466383333 0.02798

ATG2B 1.728783848 0.027999

ARHGEF12 -3.356931736 0.027999

ENST00000293201 0.902377074 0.02801

CLDN5 -0.72889533 0.028013

HEXA -22.69952986 0.028024

TMEM138 2.016005905 0.028024

GLRXL 1.169079237 0.028035

PIF1 1.736356898 0.028036

C8orf76 1.905996927 0.028042

VIM 1.107730132 0.02805

A_24_P238996 0.707983745 0.028051

FAM82C -1.763058878 0.02807

IREB2 -1.845007132 0.028074

BNIP1 2.205040658 0.028104

RNF11 -1.003767772 0.028108

SGK269 -2.339088665 0.028111

METTL2A -3.205805652 0.028116

A_32_P64894 -2.984687169 0.028119

CAPN6 -1.317158955 0.028124

A_24_P941404 -0.564103356 0.028179

DGAT1 1.015783605 0.028192

AFF3 2.021395445 0.02827

EDA 2.147300342 0.028285

AF098968 1.761718977 0.028302

ARPC2 1.286932669 0.028323

THC2660032 17.06675745 0.028332

THC2680363 5.052810854 0.028366

MYST3 1.305565258 0.028369

RPS9 1.491749503 0.028374

FAM105A 1.831323862 0.028416

BCAM -0.343482337 0.028416

FXYD1 4.44713206 0.028424

GPR68 1.36021345 0.028428

MYLK2 2.391221597 0.02846

DTNA 20.24586178 0.028464

VAMP4 -1.704081448 0.028471

THC2672305 -3.280669092 0.028489

ACSM2A 2.830426563 0.028512

DES 2.012674844 0.028532

ZDHHC8 -0.539836814 0.028535

AF420438 16.07976143 0.02854

DERA -1.158247836 0.028544

E2F2 -0.606784963 0.028557

PPP1R2 -0.701560682 0.02859

CENTG2 -2.714127051 0.028592

MGC70863 -0.881293923 0.028606

TCF21 -4.985902477 0.028617

RPL23 2.067980174 0.028621

PTGES3 1.63400918 0.028626

AK123750 -2.001568967 0.028679

ACO2 -1.748992014 0.028685

SLC6A15 6.3170493 0.028701

R96556 -0.595368852 0.028703

MIAT 0.661407533 0.028714

LMNB1 0.494641294 0.028716

IFNGR1 1.106957677 0.02872

SYNGR1 -1.05554153 0.028725

RHCE -1.29798229 0.028741

BX108033 -0.641606667 0.028743

ACAA2 -0.754238621 0.028753

ZNF462 2.817994407 0.028767

BX648207 1.372682752 0.028821

TKTL1 -0.775727654 0.02883

SPTAN1 1.183603424 0.028844

ORMDL2 1.841388665 0.028878

NOL6 -1.152489692 0.02888

FOXJ1 -0.577231044 0.028888

LOC751071 2.699113679 0.028889

RIBC2 -2.103200521 0.028907

RNF41 2.513242357 0.028946

NCOA1 1.325229053 0.028946

A_24_P392947 4.559361594 0.028953

BQ072652 1.787003528 0.028958

DAP -2.644533338 0.028995

THC2645960 1.221478044 0.028999

NSUN5C 1.026260973 0.029001

SLC39A5 1.421735019 0.029013

SGMS1 1.536047531 0.029046

XR_019253 1.641533266 0.029054

REM2 2.21166813 0.029057

THC2689579 -0.419831481 0.029094

ZNF576 2.489112074 0.029102

MAST2 2.047165625 0.029103

DSTN -0.979044393 0.029116

AK054562 1.554231292 0.029162

CDC123 1.375047035 0.029163

SDSL -1.392034514 0.029184

THC2606490 2.109870698 0.029186

THC2650012 2.049689542 0.029199

KIAA0323 -1.429977051 0.029201

IKIP 0.987210334 0.029202

A_24_P789842 0.953797461 0.029204

AI263220 3.814574311 0.029207

LCOR 1.674608324 0.029221

PWWP2A 1.405694619 0.029228

PDGFA -0.769795383 0.029267

SAP30BP 2.48778899 0.029268

GCH1 -1.49043857 0.029292

HNRNPA3 0.984646176 0.029302

DNAJC4 -0.601171127 0.029304

AGRP 0.594963386 0.029306

C6orf211 1.424695816 0.029336

C14orf128 -3.534939095 0.029343

SV2B 2.159698801 0.029348

AK056260 1.028169454 0.029352

A_24_P937806 14.26342223 0.029384

MESTIT1 -1.317076087 0.029387

ENST00000323057 1.090555599 0.029397

A_32_P148407 1.674658974 0.029409

ATP13A2 2.109785669 0.029447

MGC4859 15.67662034 0.029448

CA428801 -1.553319363 0.029451

ENO2 1.306328493 0.029453

A_24_P917530 12.32582845 0.029456

C9orf127 2.435951373 0.029463

ABCF3 1.260933728 0.029483

GAGE7 0.949566686 0.029484

RAVER1 -0.362528131 0.029518

BC035247 1.996561268 0.029521

CHMP2B 1.403447199 0.029549

LRP1 1.599695617 0.029558

TMTC3 -6.339257269 0.029565

GAD1 -0.543879525 0.029568

AF035020 1.223817024 0.029574

ZNF625 6.093107234 0.029574

TCERG1L -2.045594793 0.029582

MAP4K3 7.781613247 0.029593

RTF1 1.653037964 0.029598

FAM124A -0.433411929 0.029648

LYZL1 -0.578915933 0.02965

CYYR1 2.497241366 0.02966

PSME3 1.835744074 0.029683

RASGRP4 1.55843118 0.02969

LYPLAL1 1.46051375 0.029725

THC2621222 1.143055042 0.029749

C1orf152 -0.849872929 0.029775

MTO1 1.498216532 0.029796

BCKDK 1.067899369 0.029836

C11orf39 -3.755506852 0.029859

KRT81 -0.531970472 0.02988

BLOC1S2 1.603587269 0.029887

NFKBIB -0.433611779 0.029889

RNF14 -1.552502036 0.029889

SSH3 1.319612064 0.029899

PIGB 1.209620625 0.029908

A_24_P600603 1.728986215 0.029911

MAN2B2 1.506930247 0.029921

THC2657931 1.553617618 0.029922

A_24_P915566 -38.6842328 0.029926

PCDH10 13.44204293 0.029937

LAMB1 -4.546569667 0.02994

GPR108 1.073757505 0.029965

MAP1LC3C 6.199026198 0.030034

LOC391655 1.010140383 0.030057

SRGN 0.824715709 0.030059

CECR2 -0.47785059 0.03009

KITLG 8.111906033 0.030096

BX090181 -0.534579993 0.030121

CLEC1B -0.842044215 0.030135

FZD1 -0.911690264 0.030145

SNX1 1.317597733 0.030147

AW276186 -1.773146004 0.030151

CPLX2 -0.374640096 0.030162

TLN2 -2.296925496 0.030191

RAPGEFL1 2.721144194 0.030211

A_24_P161733 1.265840907 0.030213

COQ7 2.244765214 0.030241

VPS53 -2.673709145 0.030248

LOC389073 12.42265852 0.030256

CNOT1 2.455959008 0.030291

A_24_P916338 2.150281066 0.030298

CD59 1.239015794 0.030299

FAM83A -7.300338478 0.030316

EPHA4 1.229298264 0.030328

ATP6V1E1 1.137732296 0.030329

SLC44A1 1.353232507 0.030344

AY047582 2.012267256 0.030368

ADD1 1.974167078 0.030373

COL27A1 -0.56954969 0.030375

THC2729213 1.043327347 0.030393

ARSG 1.664519336 0.030402

NRM 1.900839635 0.030416

LRRC8B 1.630085687 0.030466

TMEM26 24.34744735 0.03047

PRR13 1.265693104 0.030486

FBXO24 1.63162097 0.030492

CPT1B 0.921591231 0.030525

THC2676737 -1.913077175 0.030534

ZNF691 1.923669947 0.030542

C18orf2 6.624793869 0.030546

ENTPD3 14.73846669 0.030569

PSMD13 1.25763516 0.030592

RP11-216L13.5 2.26964905 0.030605

CDC42EP1 -0.66962816 0.030616

BF088423 -0.496936585 0.030618

CD80 -1.217081948 0.030627

AF085874 -0.422137783 0.030633

FLJ22659 1.061325051 0.030647

NegativeControl 14.49853562 0.030648

ZFAT -1.094433918 0.030649

LMAN2L 2.188211439 0.03068

LOC100132640 0.554550447 0.030687

XR_019052 0.992903483 0.030704

A_24_P281443 1.692435899 0.030732

A_24_P324074 0.627289601 0.030748

TNFRSF4 1.176242696 0.030758

CASP4 1.13291625 0.030759

BCL6 0.774512093 0.030764

CV323908 7.989116238 0.030787

KIF7 3.251508302 0.03079

ELL 1.175107687 0.030799

CPD 0.856827741 0.030799

THC2505819 1.738240874 0.03083

PDE4C 0.766629258 0.030833

A_24_P915030 10.82867616 0.030849

RELA 1.071428016 0.030862

MAP9 1.427804825 0.030881

TRIM9 -4.23693435 0.030914

LOC388503 3.807432823 0.030921

LOC100130950 -0.443436074 0.030939

ENST00000307533 1.025073488 0.030957

COQ5 1.278212251 0.030969

PKD1 2.038030831 0.030988

AF117899 -0.509483532 0.030988

SRPK1 0.836752221 0.03099

RAB4A -1.24892389 0.031014

A_24_P761386 1.399894335 0.031033

DLC1 -1.344263117 0.03104

C1QTNF2 1.527804142 0.031048

RCSD1 1.189202252 0.031067

THC2500587 -0.89927342 0.031083

BF478043 9.954981976 0.03109

AK021933 -0.501453637 0.031092

ICAM2 -1.246764159 0.031116

AI971459 1.56521432 0.031138

ALS2CL 1.621088235 0.031154

ZKSCAN5 2.906409972 0.031159

ZNF596 4.366959803 0.031161

DDX46 1.427844817 0.031166

NUP85 1.727931223 0.031172

NEDD4 2.046959407 0.031192

RPLP0P2 1.079203287 0.031194

SOX4 -0.949095755 0.031198

DIO2 -1.754222428 0.031218

A_32_P62137 0.894785267 0.031222

THC2504193 2.355271487 0.03123

BQ548663 1.302425458 0.031258

BC002741 -5.414386959 0.031284

OLR1 5.287327773 0.031294

CSF1R -1.001760788 0.031324

A_23_P143811 -0.932213221 0.031325

MYOG -0.420786769 0.031362

RAP2C 1.57242733 0.031377

TAS1R1 -0.511148357 0.031379

SUSD1 -1.149147752 0.031381

PLK1 3.613783624 0.031389

RAB11FIP4 1.136547721 0.031396

LILRA1 -0.969538857 0.031455

RPL29 1.163415347 0.031457

SELV 2.533341298 0.031466

BC041686 1.841227708 0.031497

CMTM5 -0.713367652 0.031548

A_24_P204454 1.404531298 0.031564

C22orf24 1.54104588 0.031613

ENO1 0.951336412 0.031624

LOC100129553 1.012366902 0.031639

ERICH1 -0.804597831 0.031645

MGMT 1.553920547 0.031649

HNRNPUL1 2.178931522 0.031671

KBTBD4 2.407959493 0.031692

WDR54 1.314353139 0.031699

ARHGDIB 1.434170889 0.031745

THC2657493 14.41458835 0.031768

IFT122 1.654998429 0.031786

BF115114 -3.249510598 0.031798

VNN2 0.73061925 0.031799

C19orf61 1.050953967 0.031814

ASGR2 0.742353948 0.031867

PFKFB4 0.664694735 0.031875

SLC25A29 0.840085282 0.031913

TNNI2 0.953787081 0.031934

MRPS21 1.574885024 0.031939

PYCR2 -1.239014418 0.031952

CYBB -0.682755831 0.031956

LOC493869 13.36142064 0.031957

OR10A4 -0.441585945 0.031968

EGLN2 0.796982195 0.032016

POMT2 2.357744971 0.032027

BCGF1 0.701121849 0.032029

ADA -1.070246078 0.032035

MTMR2 -1.35416484 0.032042

CSN1S1 -1.041167377 0.032049

GSTO1 -1.511291419 0.03205

THC2560098 1.001911651 0.032059

PITPNB 1.754236476 0.032081

THC2649348 -0.498525167 0.032082

AT_L_5 19.89495636 0.032088

C7orf44 1.603090211 0.032104

AI207522 1.397236359 0.032106

VNN3 0.741486977 0.032112

NLRP6 1.651163665 0.032119

NDUFB10 1.356448923 0.032122

OGG1 -1.111802098 0.032167

A_23_P414771 -1.183082846 0.032178

SQRDL 1.136906133 0.032202

RCE1 2.090098017 0.032212

OXTR -0.424095764 0.03222

MEGF11 2.034208393 0.032221

TPI1 1.611930571 0.032244

THC2532996 -1.504796499 0.032253

STX6 1.964336716 0.032264

MOGAT1 3.253234208 0.032279

MGST1 0.970670821 0.032292

A_23_P130639 -0.51977736 0.032294

IK 1.633500333 0.032297

RSC1A1 -1.30477842 0.032302

CACNA2D2 -1.351444093 0.032306

MYT2 12.41087924 0.032306

ZNF788 7.564093631 0.032314

HLTF -0.92293592 0.032344

GAN 3.2127563 0.032348

TMEM109 -0.798869844 0.032355

ENST00000339120 1.002371614 0.032359

MOCS3 -2.69400519 0.032363

GALNT1 1.543250392 0.03237

EIF2S1 -1.989602209 0.032371

CALD1 -0.816536839 0.032377

PLXNA2 1.754414401 0.032391

HMG2L1 2.345137224 0.032395

CLDN12 38.11977402 0.032399

CCL13 4.126604257 0.03241

OR1S2 -0.686552194 0.032415

CD79A -0.440725647 0.032418

TTC3 1.616433041 0.032431

BF956667 20.22921319 0.032432

APOL1 -0.767751498 0.032443

DDX53 56.08492553 0.032514

PMM1 1.828077926 0.032519

MGC2780 2.661460471 0.032541

THC2620595 -1.528668144 0.032551

A_23_P149270 -2.612493801 0.032553

AB002438 12.64618811 0.032554

DIRC2 1.170526287 0.032567

LOC100133287 4.349335649 0.032575

CABP5 -0.433952617 0.032579

LIFR 6.728851739 0.032582

PSMD8 2.56831884 0.032629

SEMA3E 13.30718725 0.032642

HIST1H2AC 1.195922477 0.032644

BX095557 14.59430246 0.032655

METTL1 2.12153913 0.03267

PARP6 1.242933937 0.032675

THC2633447 2.933559891 0.032678

THC2594845 1.103946076 0.032679

ENST00000299125 1.38030172 0.032681

KLHL15 6.591515541 0.032694

OXER1 -0.483216668 0.032715

SYCE1 4.982202115 0.032725

PDLIM1 -1.28308223 0.032728

PISD 0.75730102 0.03274

NAMPT 0.693931525 0.03274

ZNF451 1.679600128 0.032748

ERC2 -80.04034085 0.032759

XR_018975 1.018689308 0.032768

LOC4951 2.626353887 0.032819

DSC1 -2.50619788 0.032832

C12orf61 4.905567373 0.032849

A_32_P120014 -0.400105355 0.032853

SVIL 1.064015022 0.032854

OR1L3 -0.632350132 0.032882

GAPDH 0.929139841 0.032907

FTCD 2.696203398 0.032914

A_32_P128952 53.37376927 0.032921

A_24_P921862 14.5442709 0.032928

RAPGEF1 1.002460311 0.032936

PDHA2 4.67725867 0.032951

STX12 1.841694005 0.032951

A_24_P929413 -1.880281043 0.032953

DHX9 1.169262597 0.032964

KEL -1.347376997 0.032966

SNX10 1.287393897 0.032981

CIITA -0.767059277 0.032989

SCARB2 -5.135694137 0.033

ID2 -1.166526676 0.033008

NIT1 1.47983888 0.03303

TTC22 -0.789516754 0.033041

AW804491 -0.987659083 0.033045

ANKRD33 -0.526757234 0.033055

FGF3 -0.379888248 0.033075

EFNB3 -0.776285014 0.033076

BNIP3L -0.866274035 0.033083

MED13L 1.069403638 0.033087

CRAMP1L -0.529922972 0.033138

BG695979 0.972519987 0.033172

PANX2 1.033295422 0.033183

THC2696194 -65.54875641 0.033199

PCYT2 1.47469194 0.033213

ZNF518B 1.648845722 0.033217

PFKP 1.164525059 0.033224

BE531123 2.219487816 0.033235

THC2660732 3.885398097 0.033237

METTL9 1.410195634 0.033256

ATF5 -2.170529349 0.033262

TMEM16J 0.973501163 0.033265

ARHGEF11 1.656609401 0.033267

C1orf163 2.549361434 0.033292

BM697215 25.63442364 0.033294

RPL21 -0.835649668 0.0333

EFNA1 -0.710912316 0.033326

SETD8 -1.013219317 0.033357

ENST00000390630 1.270853065 0.033364

GART -2.533259767 0.033372

ADRBK2 -1.463672056 0.033381

BG208131 2.294216156 0.033405

PCNT -1.455351878 0.033413

C16orf72 1.370704096 0.033419

PXMP4 -1.893931196 0.033422

CABIN1 1.465367151 0.033425

XR_018860 -3.318406913 0.033441

HLA-G 1.212265541 0.033444

EPPK1 -1.170664463 0.033455

WWOX -1.300057941 0.033456

LOC100133075 0.860660292 0.033477

C1RL 1.065673775 0.033507

GGT3P 1.297654686 0.033509

RIOK2 1.269496739 0.033527

MAGEA8 -0.786126931 0.033532

ENST00000320010 -1.046867739 0.033546

CYP24A1 -0.753608385 0.033549

EGFR 33.9612716 0.033551

AF007190 4.966873957 0.033577

C3orf35 2.502219392 0.033595

AB070620 -69.95334111 0.033599

STEAP4 0.914069665 0.033618

A_32_P44987 -2.155967894 0.03363

ABCA7 0.736741165 0.033638

VPS39 0.999934332 0.033665

THC2500744 -6.79554326 0.033695

ZNF224 1.791894503 0.033719

B3GNT8 0.956206184 0.033737

AK074696 0.90028787 0.033752

UNQ6484 15.00462136 0.033766

LOC253012 -1.138351914 0.033776

SCAF1 2.365457505 0.033786

LRFN1 0.454302126 0.033797

A_32_P113845 13.73114745 0.033812

EPS8L1 -1.959548004 0.033828

CYorf15B 1.672026251 0.033846

LOC283861 4.158228518 0.03386

KRT23 0.552826738 0.033868

IHPK1 1.327226489 0.033893

MEN1 16.5652796 0.033903

PCMT1 1.882251772 0.033911

LPAR3 4.938282983 0.033927

DPY19L2 16.7581753 0.033938

NR1I2 17.01062277 0.033938

SMG1 1.565560416 0.033951

TBC1D23 1.425453288 0.033957

TMIE 2.270355748 0.033968

S1PR4 1.007425612 0.03397

XR_018781 1.05344262 0.033977

THC2632909 -1.691709345 0.033978

FLJ12078 1.492730626 0.034004

FAM76B 1.076747535 0.034006

BX096603 0.793857813 0.034014

AA635126 -2.399207891 0.03402

MCTP2 0.964040797 0.034077

RIT1 1.281284962 0.034082

CD300LB -0.741806375 0.034082

FARP1 -1.391786489 0.034098

XR_019375 0.942932417 0.034109

TYR -4.998292831 0.034143

PHLDB2 7.60345768 0.034146

KIF1B 1.102892824 0.034149

WASH1 1.15071228 0.034149

MBTD1 1.676150563 0.034152

MAP3K12 2.32285375 0.034161

TRAPPC2L 2.904288451 0.034194

AMMECR1L 2.28249201 0.034194

TMEM108 -2.911101851 0.034256

CCDC3 -1.146701754 0.034264

EPHB3 2.877471413 0.034266

ROPN1L 0.820565574 0.03427

EPHB4 2.201071086 0.034283

GPSM3 2.363360102 0.034299

MTX1 1.165867886 0.034308

HIST1H2BE 1.520518862 0.03432

HIVEP2 1.114802169 0.034349

A_24_P24332 -1.325983997 0.03436

JARID1C 1.836863539 0.034362

SNX3 -1.127797215 0.034382

RERE -0.639975252 0.034383

GTF2F1 1.6199479 0.034385

RAD9A 3.304660515 0.034401

MLKL 1.350105919 0.034419

SOX3 5.561724144 0.034427

A_24_P903715 -0.497701893 0.034485

C19orf20 1.815996088 0.034493

CHRM3 3.818245498 0.034501

A_24_P912186 13.36207839 0.034537

BQ638334 -1.570700479 0.034559

ATG4A 2.027129632 0.034675

INSRR 4.199262143 0.034678

IL3RA -1.147146283 0.034683

ZCCHC2 1.473864184 0.03471

FLJ35848 14.73761268 0.034718

NSUN5 1.172663442 0.034728

ZNF570 -4.279767878 0.03474

CXCL12 -18.05810339 0.034785

PDZD7 1.074040788 0.034787

PTPN7 1.078125254 0.034814

WASF3 -1.471974021 0.034834

KRTAP3-1 -0.937426816 0.034845

ZNF138 -1.690313516 0.034855

CD36 0.893554197 0.034872

MCOLN3 -2.834261135 0.034881

AW337952 4.048474658 0.034883

ANKRD12 1.920255756 0.034885

XR_019523 2.985283404 0.034908

AX721299 8.484354613 0.034917

NR1H3 -1.672946286 0.034923

MANBA 1.415474061 0.034927

CDK7 1.768076981 0.034951

AF229803 3.591704844 0.034973

NLRP12 0.896396253 0.034995

SMC6 1.458116125 0.035003

THC2616558 6.678640109 0.035011

B3GNT6 -1.808641509 0.035013

FN1 3.668615929 0.035034

SLMO2 -1.22555772 0.035049

ZNF507 -2.852641987 0.035067

TIAL1 1.600694349 0.03508

FAM3D 1.547814047 0.035095

OR7E24 -0.68510846 0.035098

MICALL2 -0.795181814 0.035105

ENST00000400371 12.27086527 0.035131

ABHD14B 1.336219048 0.035136

A_23_P256973 3.37634244 0.035149

MOGAT2 14.08309404 0.035207

DKFZp434F142 -0.653631256 0.035212

ETS2 1.107309402 0.035219

NDUFB4 -1.201354003 0.035234

LOC728533 1.380176228 0.035241

NLRP10 18.12847326 0.035257

AW134708 3.351621318 0.035266

LOC100131170 0.579182071 0.035288

ELAVL4 12.19084396 0.035296

CMKLR1 -1.102821466 0.035323

SLCO3A1 -0.959576984 0.035324

TTC9C 2.093673458 0.035332

THC2642612 -0.444808999 0.035386

ULK3 -1.437487544 0.035401

TM6SF1 -0.871434889 0.035404

BF509345 3.170938793 0.035485

EEF1D 1.125689264 0.035492

BC038355 1.881159853 0.035519

THC2507792 -1.371988622 0.035525

TRIB1 0.86302902 0.035532

ORAI3 1.351781335 0.035533

GABPB2 2.278869916 0.035546

AF090926 1.221713088 0.035573

A_32_P74778 -1.307876764 0.035574

C10orf137 1.952076907 0.035601

IL12RB1 -0.520341208 0.035614

AF116626 1.251511931 0.035635

ZFP1 2.162691959 0.035648

FAM43B -0.626770209 0.035649

AW302767 1.513677502 0.035662

BCL2A1 0.719947102 0.035668

MGC39821 -0.469456717 0.03572

CRTAP 2.679833164 0.035725

STOML2 -1.498111456 0.035735

RPS10 1.449768606 0.035741

C1orf53 -1.624306615 0.035744

A_24_P600036 -0.767840515 0.035754

AW452819 -0.600565348 0.03578

KCNK17 -3.207403871 0.035786

BX095281 1.793948588 0.035819

AK098597 1.65588168 0.035847

ZNF524 1.758968283 0.035852

EIF3F 1.269736369 0.03587

THC2664263 2.095537443 0.035892

ARG2 -2.209159206 0.035924

C4orf41 1.687280588 0.035934

SLC30A10 2.73634762 0.035934

LOC729611 1.406749793 0.035975

NKX1-2 -0.635977768 0.035979

ENST00000329015 -1.483118221 0.035988

GLI2 -2.303169901 0.035992

XR_018514 4.485003513 0.036049

GIT1 2.075791669 0.036067

PDGFC 38.9365579 0.036068

SPATA1 -1.815798924 0.036074

LRAT -0.496695959 0.036101

AL832451 1.019247932 0.03611

OPRL1 -0.422244572 0.036116

CHI3L2 -1.289620777 0.036117

RNF135 2.61410469 0.03612

MPPE1 1.215423444 0.036139

LOC644063 0.629047516 0.036146

ENST00000255896 -0.928679982 0.036159

SGSM1 1.635753084 0.036165

PCP4 -1.23131703 0.03618

TNP2 -0.762013088 0.036208

HLA-F 0.953471024 0.036221

A_24_P922758 14.31030267 0.036241

KLF4 -1.904889329 0.036247

YWHAE -2.058258765 0.036267

CNTLN -0.840828857 0.036295

LOC100129742 0.862030425 0.036315

CLTCL1 1.482124645 0.03633

A_24_P298238 0.934827037 0.036335

DCAKD -1.141727831 0.036372

VTI1A 1.63745031 0.036401

C9orf125 -1.827699063 0.036404

ZNF619 -2.307361612 0.036413

CR606630 1.084420639 0.036422

PRSS27 2.919527673 0.036443

SYNC1 -2.052344078 0.036446

ORM2 0.55048458 0.036446

PRDM5 1.415651485 0.036454

SMPDL3B 2.062162925 0.036458

A_24_P214556 1.302511868 0.036461

KIF27 2.394090716 0.036486

SLC2A13 -1.759860593 0.036503

ME2 -0.804524493 0.036539

XR_015535 -0.763820291 0.036572

PSMB1 1.241286668 0.036573

LOC144097 2.854050075 0.0366

BC027922 1.617097989 0.036608

TPR 1.393880028 0.036647

TRMT1 1.387950722 0.036648

AF119861 1.77231324 0.036663

UQCR -1.953999983 0.036664

UHRF1BP1L 1.213967385 0.03669

XR_018462 1.515252278 0.036709

THC2494410 1.277017021 0.036728

AK091942 -2.24408528 0.036732

ACOT7 -1.315889196 0.036735

BC089156 1.276346506 0.036756

INTS8 1.459942798 0.036772

A_32_P99804 -1.946424213 0.03679

GAB1 10.32894303 0.036806

NT5DC1 1.461179222 0.036816

ENST00000324654 -1.678713816 0.036824

ADD3 0.913124022 0.036848

A_23_P96262 -2.448189638 0.036857

BG108194 1.188457644 0.036871

PNPO -1.167875126 0.036875

A_24_P887805 -1.04844657 0.036884

HAND2 -5.706239602 0.036892

GPR114 -0.943721869 0.0369

ARL8A 2.312826071 0.036905

HIPK1 -2.464001002 0.036913

MSL2L1 0.860115828 0.036925

PLEKHG4B -1.488686556 0.036946

RBM17 1.203671539 0.037011

A_24_P910718 13.3739113 0.037034

AK129743 1.48808709 0.037038

KDELR2 -0.953919501 0.037054

PAFAH1B2 1.92455572 0.037081

LOC644937 0.987529459 0.037103

MED14 3.136439115 0.037109

CHD7 1.010861434 0.037116

WBSCR23 6.127744679 0.037126

A_24_P926709 -2.199742335 0.037138

MRGPRF -0.43219654 0.037151

MMRN2 -0.747013368 0.037155

KCNH3 1.490483107 0.037197

CXXC4 -4.042933128 0.037219

A_24_P407742 -3.507617297 0.03722

H45075 -1.300639082 0.037234

DRD4 -0.919884699 0.037246

CYLD 1.039684517 0.037254

CDK2AP1 -1.262672323 0.037257

ACBD4 1.464215355 0.037304

ARSA 0.815435581 0.03732

TNFRSF10C 0.83269449 0.037331

ANKZF1 1.148851722 0.037335

SLC2A14 0.789610792 0.037364

EML3 1.125894075 0.037367

AA020958 10.79888957 0.037369

ERP27 1.501265131 0.037398

NEXN -0.782542985 0.037401

BI832578 -2.271186114 0.03741

HTR7P -2.378095699 0.037415

THC2543840 1.221924172 0.037431

CCDC96 -1.357853653 0.037432

PACRG -0.520206707 0.037466

BC068044 1.246105726 0.037498

SERPINF1 -0.942258195 0.037508

BF931515 0.953454992 0.037514

AT_T_M 8.3793022 0.037523

EIF3K 1.127369253 0.037525

THC2701869 -1.646260093 0.03755

IRX6 -0.703815504 0.037553

MYO1C 2.246537755 0.037559

AK001120 1.283847172 0.037559

GBA2 1.264319914 0.037583

AF038199 1.59567168 0.037596

PXDN -0.574584732 0.037633

A_23_P119168 1.122098225 0.037683

MED6 2.025776445 0.037708

AF085920 -3.638752459 0.037748

FRAS1 -0.919392313 0.037794

SLC4A3 -2.58871834 0.037824

C20orf59 -0.524763696 0.037849

TTLL6 15.79735711 0.037883

TPRXL 2.777880964 0.037887

A_24_P76008 -0.523632847 0.037924

CYP11B1 -0.405447471 0.037947

TFF1 -0.490472503 0.037951

SPOCD1 -0.641105899 0.037962

CR617352 1.143783644 0.038004

FLJ10404 0.934908484 0.038007

AKAP11 1.173463591 0.038008

BC073929 2.063041396 0.038034

IKBKAP 1.534037629 0.038055

CENPO -3.631725488 0.038111

CLDN3 -0.603311539 0.038142

PLEKHN1 1.238384981 0.038149

A_23_P208582 -1.229451142 0.038152

FADS6 2.121471461 0.038171

ACTR5 -0.512439544 0.038174

B4GALT2 12.53947718 0.038175

KLHL32 -4.045243446 0.038176

ORM1 0.48237136 0.038181

RARA -0.408668261 0.0382

CDC42EP4 -0.669422597 0.038206

ADRA2C 1.520387122 0.038211

MUSTN1 1.593208552 0.038218

AMT 1.381646399 0.038224

CD58 1.306980202 0.038243

BC011648 2.241994468 0.038275

A_24_P452326 2.056236662 0.038287

BC037918 1.834237021 0.03829

SFXN4 -0.791215091 0.038292

LIPG 8.697045087 0.038307

SPHAR -1.107159958 0.038325

THC2654273 -65.73013443 0.038338

SYVN1 2.227067925 0.038345

INF2 1.218835614 0.03837

C16orf79 1.245134366 0.03839

RMND5A -0.951257085 0.038402

LOC340508 -0.985939456 0.038417

AK094525 -0.676981265 0.038417

LOC284033 -3.848323292 0.038446

RNF44 1.323852478 0.038447

ING4 1.630652648 0.038456

A_24_P578641 1.770736034 0.038459

ZFHX3 -1.105014795 0.038464

AMICA1 0.861105944 0.038469

THC2681437 6.092238629 0.038496

ACOT11 -0.860872311 0.038505

AK091335 1.072014773 0.038512

SPG3A -2.322529228 0.038515

SPAST 1.838095749 0.038566

RAB1B -0.53919363 0.038622

NP1247838 1.764291777 0.038636

AA704712 -1.02949401 0.038641

PGS1 1.64270446 0.038641

ADAMTS1 -4.224117143 0.038645

AI492422 -7.762402025 0.038678

DB518505 1.408308222 0.0387

ZC3H14 1.947245887 0.038741

BTN3A1 1.3018033 0.038754

CIZ1 -0.596801595 0.038758

FAM30A 0.782150969 0.038765

XK -0.502732884 0.038812

KIAA1833 1.338480046 0.038843

LGALS12 -0.848198042 0.038894

TLR2 0.743921506 0.038906

AK001116 0.616453612 0.038908

MEP1A 2.920951799 0.038912

BQ029628 -1.500881296 0.038947

C1orf144 1.433136084 0.039003

THC2672257 15.15447178 0.03903

FLJ20309 3.425910745 0.039045

ABCB1 -2.011271392 0.03905

LOC285398 3.610722212 0.039067

SS18L2 1.600681511 0.039077

C14orf115 1.609546803 0.039093

AW293859 -65.1221687 0.03912

C17orf59 -1.155682415 0.039131

GMFB 0.944006093 0.039137

C9orf100 -0.717102225 0.039142

AK026140 -1.999670239 0.039147

ATP6V1E2 1.569539325 0.039178

KIN 1.539220887 0.039267

OXA1L 1.363261208 0.039279

B3GNT7 -2.066709905 0.039281

C22orf37 1.143382994 0.039282

SMAD1 -0.927822232 0.039302

COX19 1.202741761 0.039303

SCML1 -2.433331832 0.039311

HPS3 1.658973316 0.039327

ROCK1 1.198458839 0.039328

RHBDD2 1.404661462 0.039343

CETN1 2.303916825 0.039361

SLC5A5 2.715255409 0.039382

ARL4D 1.938838581 0.039385

HIST1H2AD 1.037870507 0.039457

MRPL20 1.55811317 0.039463

PDGFRB -1.118252755 0.03949

ENST00000308384 -0.800379085 0.039493

S66917 -0.532111443 0.039496

AOC3 0.956147007 0.039607

BLZF1 1.741153502 0.039624

TRIM24 1.11394716 0.039627

BC014215 -0.536792601 0.039632

ATP6V1G2 -0.496342443 0.039673

A_24_P452293 0.801491001 0.039679

A_24_P417751 3.229471872 0.039692

FOXN1 -1.010375154 0.0397

PAN2 1.96186318 0.039703

AGBL3 -0.63562406 0.039703

CADM3 -0.572833268 0.039733

TARBP2 1.829626379 0.039736

GPX2 -1.095751659 0.039738

A_24_P896373 -1.039712305 0.03975

ARL6IP1 -0.694367677 0.039761

MORF4L2 -1.996597909 0.039769

BCL2 0.954490385 0.039797

ENST00000391481 -0.633015604 0.039811

LRRC8A -0.958757502 0.039823

PDDC1 1.470429351 0.039839

C7orf40 1.390374689 0.039849

FLJ30679 -3.310552723 0.039902

BQ434958 -1.762084895 0.039916

CR627122 -0.524506339 0.039919

DAPK2 0.705764086 0.039961

A_24_P925455 1.362614587 0.039985

VKORC1 2.18421224 0.039989

STK17B 0.986311676 0.040001

A_24_P931711 -0.441739519 0.040035

ASB4 -0.682540513 0.04005

DUX4 -0.799230838 0.040081

FLJ33590 1.838876045 0.040083

LOC649839 1.454635836 0.040088

NUDT5 1.044589185 0.040093

CCT3 1.123327092 0.040105

ECSM2 4.49371451 0.040138

DULLARD 1.549385033 0.040153

A_32_P185089 -0.337655443 0.040171

THC2630830 -1.472020548 0.040174

CDH16 3.584393737 0.040211

ZNF467 1.307927779 0.04024

POP1 -2.602462028 0.040242

ENST00000317571 1.388255994 0.040255

GTPBP1 1.345292509 0.040271

A_24_P827738 -0.731682683 0.040288

RBM28 1.962777857 0.040315

MCM3AP 1.292088034 0.040359

RTP4 -0.906538005 0.040361

KCND3 -0.539281429 0.040374

RAB43 1.112507632 0.040388

ENST00000398290 -0.369820106 0.040402

ZNHIT1 1.800551694 0.040405

A_24_P927996 2.220009331 0.040416

SORBS1 -0.451720329 0.040448

ADAM17 -1.184553791 0.040449

TM2D3 1.340402178 0.040456

THC2526402 0.880908048 0.040486

TTC23 -63.93316958 0.040493

DEAF1 1.765460392 0.040498

A_32_P19466 -0.831018336 0.040508

C12orf51 -0.519493321 0.040524

NDNL2 -1.059004528 0.040525

SERPINA1 0.889045292 0.040527

ATF3 -1.389580311 0.040531

AA242987 -1.444815374 0.040552

ENST00000378954 1.222949185 0.040553

A_24_P306788 2.496946372 0.040554

TCF19 3.279687873 0.040585

SCNM1 1.217862877 0.040604

A_24_P824642 46.82268376 0.040604

XR_019167 -0.376324043 0.040608

A_24_P383199 1.935343155 0.040621

H2AFX -1.042143499 0.040625

RBM39 1.235948814 0.04063

ENTPD4 1.804303628 0.040637

AFF2 12.3458786 0.040647

BC017945 10.14354316 0.040657

LCE1A -0.386276972 0.040672

FCHSD2 1.717650044 0.040702

AB019568 -0.654014431 0.040744

SLC12A9 1.167771575 0.040755

EFNA4 1.946873098 0.040759

EMR3 0.855702571 0.040765

PSEN1 1.097518751 0.040769

AK055067 2.65227384 0.040791

AK096104 1.788616787 0.040801

TBC1D10C 1.103170036 0.040816

ADRB3 -0.602468656 0.040836

T12590 -0.734158956 0.040848

COL1A1 3.515455357 0.040856

NADK 0.830538045 0.04086

AT_P_3 17.24365556 0.040901

IQSEC2 -0.534262017 0.04091

C6orf106 1.179745913 0.040912

PLAC8 -0.983339915 0.040922

LOC728732 0.853330035 0.040923

TTBK1 -0.575280544 0.040957

TMEM161A 1.419037135 0.040958

UBXD3 -1.314456006 0.040961

CD160 -1.063178476 0.040964

ZNF700 2.016799195 0.04097

ZRANB1 -1.290039376 0.040975

IER3IP1 -1.029185941 0.040981

C11orf75 -0.881625579 0.040992

CHRNA6 3.843822348 0.041008

INTS4 2.106112221 0.041011

JUND -0.925007875 0.041036

SLC35F5 1.331499739 0.041041

ENST00000390550 1.986187758 0.041071

THC2694873 -2.691645395 0.041098

A_24_P384210 0.567874948 0.04111

THC2608799 -1.139742893 0.041113

VRK3 0.985149594 0.041151

ARFGAP1 1.110481909 0.041153

CCDC95 1.443578264 0.041172

A_24_P392790 0.70899822 0.041183

M6PR -1.143981705 0.041204

DMRT3 24.79089323 0.041238

VPS26B 1.060882569 0.041256

FLJ36848 -0.641625372 0.041259

PKMYT1 -1.001575155 0.041281

LOC100128140 0.636438116 0.041289

BC019599 1.313206212 0.041307

RABGAP1 1.129884434 0.041329

DUSP16 -0.657968345 0.041373

LOC283624 51.4051288 0.04138

BC062324 0.809777929 0.041386

GLCCI1 1.534928165 0.041407

CLIP4 -2.607501371 0.041418

BE243669 -0.618217538 0.041426

APOB 16.48143053 0.041452

THC2506246 -1.781658637 0.041503

AA669846 1.958431365 0.041509

LNX1 -1.143836116 0.041525

PEX14 1.824616437 0.041544

CLPTM1 1.928602373 0.041563

LOC126075 1.84350538 0.041607

PLVAP -1.066116944 0.041626

ZNF765 5.508307738 0.041646

ENST00000393496 -1.966054002 0.041682

C1orf80 -1.355166385 0.041697

C3orf10 -2.225944093 0.041704

LOC595101 0.556785685 0.041712

NME1 1.166661055 0.041722

PPP2R2D 1.47289151 0.041723

GLRA3 5.861539195 0.041729

VAMP2 1.542825468 0.041735

VCL -1.113685765 0.041746

OAT 1.178380925 0.041775

RAX2 1.032174426 0.041823

LCE2B -2.136775069 0.041825

AK025047 -0.698067611 0.041879

ZYG11B 1.058725637 0.041881

C8orf66 4.713153362 0.041883

EGR1 -1.126551197 0.041906

DBI 2.061477354 0.041918

AL036373 -1.210489485 0.041952

GPR123 3.457638735 0.04196

RBM8A 1.79047801 0.041971

ASPSCR1 1.635440408 0.041972

RAB3D 1.201874887 0.041995

ADPGK 1.474532731 0.042004

XR_018415 2.221121131 0.042074

NXPH4 -1.142157684 0.042076

FLJ39005 4.985939223 0.042086

XR_019510 10.16349835 0.042102

AK097700 0.957355967 0.042195

CDKL1 -1.23439763 0.042202

ARHGEF1 0.9868261 0.042227

WDR88 13.58211474 0.042227

TRAF3IP2 2.71055505 0.042249

CBX6 1.082355242 0.042259

OR7A5 11.54496765 0.042297

RC11 19.62826451 0.042299

TBC1D16 -2.586406224 0.042306

XR_015548 0.830122375 0.042309

STT3B 1.145131518 0.042348

DHRS13 0.814696147 0.04235

ZNF346 1.523073337 0.042366

CNGA3 16.37387105 0.042397

P53AIP1 1.813775818 0.0424

A_23_P75192 -0.564679558 0.042419

NDUFS5 1.406666039 0.042429

AMAC1L2 1.733528629 0.042488

SNAI3 1.264591264 0.042493

EEF1A1 0.807715156 0.042514

SEMG2 10.08239562 0.04253

DECR1 1.432231451 0.042585

RBED1 2.022058215 0.042586

PDE4B 1.231089785 0.042602

APLP2 1.444503644 0.042612

OR10G8 -0.801603696 0.04263

ARRDC1 1.596373008 0.04263

RAE1 1.638044083 0.042678

ANKIB1 -2.69798148 0.042694

FAM133B -0.711195398 0.04272

XR_016847 4.065066945 0.042773

RAP1B -1.112365413 0.042791

BATF 0.75910322 0.042795

C14orf68 -0.557493077 0.042796

OR51E1 10.01347651 0.042797

PCDHGA7 -0.418425006 0.042805

RIPK3 1.003889711 0.042847

ENST00000378250 -4.413816357 0.042897

ODZ3 -1.121265401 0.042926

GHRH -1.803120521 0.042927

C14orf43 1.260745479 0.042951

THC2691500 1.652216374 0.042963

AL547890 1.072456795 0.042966

SCAMPER 14.31466965 0.042988

TIGD1 -0.664440204 0.042996

G6PC2 -1.578510764 0.043012

A_23_P125109 0.826608037 0.043023

FBP1 -0.835645812 0.04303

XR_019258 1.060195419 0.043043

ENST00000339845 -0.678915724 0.04305

VEGFB -1.038929756 0.043051

C4orf14 -1.271981454 0.043057

RPS6KB1 1.577161573 0.043073

ZNF493 1.873230668 0.043084

SLC34A3 -0.345087735 0.043102

TREM2 3.933185061 0.043106

NINJ2 -0.728022604 0.043108

AL162073 1.944403814 0.043126

FAS 1.037247926 0.043153

KLF16 -0.544719477 0.043155

C17orf71 -52.59736796 0.043161

LOC255480 14.37788942 0.043162

ENST00000310995 -0.774139068 0.043163

THC2673062 -0.41885676 0.043163

BQ778202 2.278559771 0.043165

SLC9A9 -1.017688789 0.043168

NLRC4 0.951820087 0.043209

PRPF6 1.035197967 0.043209

MAP6D1 -0.398707163 0.043215

BC019583 -5.393820639 0.043221

UPP1 1.201252899 0.04324

TUBA1B -1.193094582 0.043252

THC2749298 -0.698791841 0.043254

RP4-747L4.3 -0.94088204 0.043259

FCHO1 1.179504808 0.043315

LYPLA2P1 1.282142045 0.043337

AF116642 0.735247567 0.043347

TTC9B -0.462104905 0.043381

MEFV 1.215473399 0.043383

C1QTNF7 -8.672124968 0.04339

CRKRS -2.679482168 0.043431

A_24_P481314 3.314224155 0.043452

TTC4 -1.372418268 0.043457

OPLAH 0.78169155 0.043477

AW771919 -1.572481793 0.043482

SGPP2 5.72713833 0.04349

ZNF638 1.523517255 0.043516

SLC23A3 1.937839394 0.043518

ZNF136 1.581937267 0.0436

AA579646 4.084389229 0.04362

LOC440934 5.404612634 0.043622

GTF2I -8.851754414 0.043632

IQCK -2.350172809 0.043635

A_32_P185530 9.243626558 0.043645

AK024602 1.123424981 0.043655

TSC22D1 -0.952471224 0.043663

IPO8 -5.112727156 0.043681

SHB -1.366378334 0.043686

APOO -1.29737961 0.043723

GK 0.940053344 0.043724

RFX4 1.53176099 0.043751

A_32_P108420 1.62186171 0.043764

AF209507 6.500877601 0.043776

MUC6 -0.891021909 0.043791

PPP1CC 1.778680687 0.043823

ATP6V0D1 0.955115224 0.043835

HM13 0.795038639 0.043853

ABCC13 -0.541455634 0.043866

TIPRL -1.610739315 0.043884

TSPYL2 -0.596673244 0.043887

RNASEK 1.89456055 0.043888

KIAA0284 1.366057367 0.043894

A_24_P7510 1.811381066 0.043899

CXCL3 -2.371361564 0.043914

PPM1M 1.095346789 0.043915

ADCY7 -0.591453092 0.043977

ENST00000381800 2.349719964 0.043981

LYPD5 -29.56863504 0.044004

HOXA10 3.071757588 0.044038

ATP7A 1.229084479 0.044039

CD97 0.967946493 0.044068

AF116661 -0.324744855 0.044118

FKRP 2.173567728 0.044119

FLJ43276 -1.106597994 0.044142

RNF10 -0.696925829 0.04416

MAPKBP1 2.753842371 0.044237

3-Sep 3.859417972 0.044265

LOC124446 -1.14996811 0.044292

U49441 15.03918268 0.044297

DCDC2 17.29755912 0.044336

PSD4 0.856710764 0.044355

C2orf56 1.994465034 0.044376

UBR4 1.004968686 0.044386

C5orf20 -1.187239224 0.044403

LOC100130152 -0.836567036 0.044405

PAF1 1.329810336 0.044469

ZNF512 1.479251014 0.044484

MLL3 1.075297295 0.0445

BC066989 -1.63380717 0.04452

AP2B1 0.67697598 0.044521

HSPBP1 -1.241541597 0.044545

ENST00000395095 -0.97213099 0.044578

CREB3L2 -1.118166691 0.044596

PHF11 0.965512751 0.044603

SPG7 3.160701322 0.04463

PRAGMIN -0.955654451 0.044641

ACVR1B -0.389090931 0.044678

ZNF23 -1.19307002 0.044683

LHB -0.350831546 0.044693

ATG4B 1.103543755 0.044711

AK096500 4.508628062 0.04473

CLCN3 -1.407716364 0.044752

A_24_P127442 0.769219414 0.044774

CD226 -1.419323683 0.044779

CD3D 0.841207778 0.044791

AK057443 3.054660937 0.044848

KLHDC6 -15.20990195 0.044856

AK024171 1.229159043 0.044905

GNA13 1.107134034 0.044917

BF475893 0.955956346 0.044921

PASK -1.974644613 0.044927

EB386378 -0.983857121 0.044977

CSDE1 -1.463401188 0.044988

C9orf128 -3.212690294 0.044997

NCF4 0.813903814 0.045007

LOC100132738 0.384282609 0.045007

MBNL3 -0.867762962 0.045015

THC2680668 -1.009377658 0.045022

A_24_P931804 -0.752153078 0.045036

AI942297 -0.541172643 0.045045

THC2664391 -1.385539127 0.045052

A_24_P187365 -0.922452687 0.045063

A_23_P13232 -1.378159613 0.045069

A_24_P912404 2.186689501 0.045071

MYOZ1 -4.802568823 0.045079

ATXN1L -0.503577659 0.045125

ACY3 -0.483798791 0.045133

LOC100130794 1.254217804 0.045146

OTOS -0.68372368 0.045146

RBMS3 16.54812179 0.045151

A_24_P332780 1.441458399 0.045155

CLPS -0.636262428 0.045158

KIAA0232 1.130518261 0.045243

LOC100132014 3.485927278 0.045279

THC2564562 1.681585753 0.045295

THC2489591 -1.407859719 0.045302

C6orf32 0.815715539 0.045322

CB052856 13.20592994 0.045384

A_24_P940738 5.099906037 0.045385

KIAA1731 1.732025033 0.045391

A_24_P556049 1.674543543 0.045408

LOC100129198 -39.28670697 0.045411

LOC56757 1.513875145 0.045424

PAQR8 -0.770471261 0.045428

ICOS 0.868177966 0.045455

RPS21 1.201220851 0.045497

C15orf44 1.591030792 0.045519

A_23_P348587 1.071607796 0.045567

tcag7.1017 1.745786351 0.045575

ENST00000382909 1.710884506 0.045594

ZNF187 1.799894759 0.045661

RPL5 0.96356339 0.045664

ACOX1 3.505374493 0.04567

CK818049 24.3136287 0.045676

NECAP1 1.90207841 0.045751

SRP68 2.103957801 0.045774

A_23_P253168 5.98265086 0.045922

A_23_P201521 0.926039383 0.045948

PITPNA 1.077322213 0.045968

ZNF223 1.709815618 0.045996

C2orf40 5.428800646 0.046027

THC2702563 -2.369660317 0.046041

SLC2A3 0.928134721 0.046046

KIAA0564 1.907536394 0.046051

A_24_P7494 1.086848269 0.046075

LOC391769 1.172571895 0.046094

AF119914 23.47942165 0.046104

CCHCR1 -1.429123715 0.046106

FETUB -0.8010547 0.04612

TAF3 -1.179407378 0.046125

INTS1 3.666265265 0.046129

DSCR8 -0.938384591 0.046148

FLJ23185 0.842388613 0.046174

FBXO34 2.026805657 0.046185

PPP1R9B 1.877343502 0.046194

SOD2 0.813986 0.046197

TOMM22 -1.562720687 0.046214

AK2P2 2.447378355 0.046215

GABBR1 -1.179548508 0.046218

C3orf62 1.222620857 0.046233

MEG3 -0.415356737 0.046236

THC2741486 -0.929114663 0.046237

OPN4 -1.007497853 0.046244

ANAPC4 1.351369974 0.046281

CDS1 20.86605564 0.046282

GRIN3A -4.35114644 0.046352

THC2656826 -0.97868623 0.046377

AF034209 -66.10009292 0.04639

NKIRAS2 -0.539363312 0.046393

EIF4EBP2 -1.251090021 0.046397

A_23_P133949 -0.941734767 0.046399

CR597597 2.249296212 0.046404

PARK7 -1.738416355 0.046404

ULK2 -1.157675954 0.046415

ADHFE1 1.32488275 0.046426

ADAM33 -0.449349312 0.046451

GPRC5D -3.019335204 0.046464

AF271776 -0.842683398 0.046473

A_32_P25809 0.94467045 0.046488

FAM120AOS 1.797002239 0.046499

ROBO3 -0.559511146 0.046519

CCRL2 -1.987810767 0.046563

BC035377 -1.281261296 0.046574

AK023104 16.95927098 0.046581

TTTY12 -0.743855937 0.046603

ORC4L 1.565059938 0.046613

EXTL3 0.875602018 0.046614

ENST00000391334 -0.740323627 0.046662

AGK 8.877240161 0.046667

YPEL5 1.550366919 0.046679

NGLY1 1.798276825 0.0467

TSPYL5 -0.976152466 0.046705

AK1 -0.533174247 0.046712

RIN3 0.595571205 0.046721

PHOX2A -0.595144154 0.046722

ENST00000397462 -0.848405386 0.046802

A_24_P42071 -0.584145204 0.046808

REV3L 1.506913687 0.04681

RAB6IP1 0.988599099 0.046814

RABL4 1.476528975 0.046818

LOC93349 1.644232037 0.046825

AK093691 1.278933311 0.04683

BC070363 -0.690430207 0.04689

TMEM145 -0.436807942 0.046892

DKFZp434L192 2.278645172 0.046897

5-Sep -0.719819173 0.0469

GRIN1 -0.327170383 0.046908

WDR19 2.410157866 0.04691

SDCCAG1 1.417451304 0.046917

SLAMF7 -0.734356775 0.04692

PIPOX -0.709052579 0.046973

THC2508355 1.031031143 0.046976

MYO1F 0.840907284 0.04699

PRKCD 0.926397836 0.047015

TAF12 1.576526244 0.047059

C15orf28 0.800649353 0.047087

C17orf62 0.866491034 0.047098

ZCCHC14 -2.993593415 0.047108

AK128396 1.759688182 0.047121

NAT8B -1.041587991 0.047171

AF063695 1.716650664 0.047175

C8orf59 -1.543627775 0.047186

HIST1H2BN 1.264968366 0.047188

RECQL5 1.242136095 0.04721

NOTUM 1.423305682 0.047219

STOML1 -1.205482681 0.047219

CD671494 5.745440717 0.047221

RNUXA 1.617492707 0.047222

MAN1C1 10.24169381 0.047223

THC2744687 -0.749105178 0.047231

RPL14 0.794751593 0.047235

CR594735 -0.607143273 0.047247

TMCO1 1.2387896 0.047256

KIAA0391 1.03776271 0.047257

WWP1 1.605579814 0.047261

SNIP1 2.020431497 0.047283

C10orf27 -0.540706174 0.047306

DQ786246 0.837733728 0.047317

ENST00000399221 0.721590249 0.047348

GHSR -0.654878692 0.047388

APOA1 2.835460152 0.047397

DB304731 0.871237999 0.047404

C11orf67 1.814552874 0.047408

CCDC32 1.676536316 0.047425

ZNF324 1.200941629 0.04747

C17orf87 -1.57942565 0.047488

KDSR 1.280568362 0.047489

LAMA3 4.927169643 0.047525

FSCN3 1.604621382 0.047532

SEC61G 1.286451512 0.047565

LOC400099 -1.294764848 0.047576

OR10P1 -1.328395326 0.047612

PLCL2 -0.934805539 0.047613

SFRS7 1.218129496 0.047641

P4HA2 2.791657455 0.047664

SOCS1 -0.831165423 0.047694

HEMK1 -0.64144861 0.047718

CDCP1 9.759313764 0.047745

CDKL2 10.6174282 0.047768

A_24_P298179 -1.71850398 0.047791

BX117044 1.660029325 0.047823

A_24_P67638 1.295539865 0.047845

TPPP3 -0.940622504 0.047847

A_32_P131090 -0.528076193 0.047849

DNAJC13 2.315674571 0.047858

BCL2L15 5.771841486 0.047863

APBB1 -0.732830744 0.047873

TNKS 5.300010035 0.047895

OSTM1 1.401919746 0.047899

LELP1 6.849248787 0.048014

FLJ35220 1.945868283 0.048021

KRT18 1.155350208 0.048074

ZDBF2 3.092081836 0.048096

SLC9A1 2.581654205 0.048143

THC2689427 -0.898707162 0.048159

AK021842 -0.623142853 0.048168

TTTY14 0.853644236 0.048182

A_23_P251196 1.099318562 0.048202

USP33 -0.697157852 0.048202

ARID1B -1.782471558 0.048224

A_32_P214860 -1.39968854 0.048229

TRAPPC1 1.206278883 0.048256

THY1 -0.309236892 0.04827

A_23_P158868 0.618544316 0.048292

MGC14425 -1.807084229 0.048298

MAP3K3 0.795287674 0.048317

WDR48 1.612565118 0.048336

TRAF6 2.554453688 0.048375

FAF1 1.584928635 0.048378

A_32_P173122 -0.921069533 0.048382

CF139200 -0.834865242 0.048392

UTP14C 1.821780322 0.048415

MTMR6 1.347780121 0.048418

ZNF778 -2.912382374 0.048433

HARS 1.488410214 0.048456

ANP32B -1.000419716 0.048464

IGSF5 -10.84644322 0.048474

HLA-DPB1 -0.561226054 0.048498

AA045093 2.550127818 0.048509

SLC22A18AS -0.327682473 0.048525

PDXK 1.05353073 0.048532

A_32_P221641 0.883303114 0.048534

TMEM30B -0.686453703 0.048545

ADRA2A -1.949164547 0.048602

NAT1 1.394415054 0.048618

LYRM1 0.992581659 0.048636

THC2670311 -0.980435233 0.048642

TNK2 2.516702516 0.048644

AL390143 -1.229312984 0.048711

GALM 1.13013087 0.048712

TRIM13 1.413438556 0.048776

LOC220433 1.046030004 0.048799

FAM132A 1.613352977 0.048863

COMMD3 1.202900625 0.048907

BC015133 1.311826392 0.048915

BG743700 19.7617666 0.048948

CD556746 0.735957761 0.04895

TMEM45A -2.034951976 0.048951

RET -11.24324915 0.04896

A_24_P229766 1.111748945 0.048976

A_32_P78395 0.642195653 0.048977

TIPARP 0.908039093 0.048994

BX105253 -0.52515942 0.049012

CLDN11 -0.763145706 0.049038

MFAP2 -0.995647546 0.049061

AF086464 16.27990302 0.049076

AF289566 5.837553054 0.049133

RAB32 1.188106793 0.049135

ZFP90 -1.662712679 0.049136

C14orf102 2.09166366 0.049159

AF086044 -0.859116745 0.04916

ENST00000377006 -1.501630064 0.049161

SLC22A6 3.846292333 0.049232

BX089701 1.000643641 0.049248

TBKBP1 -0.554578128 0.04926

TNRC18 1.032782458 0.049267

TLR9 1.734652646 0.049274

RP4-691N24.1 -0.858034062 0.049322

HIST2H2AC 1.101077651 0.049335

IARS2 1.452109399 0.049339

LRSAM1 1.53252508 0.049343

WWTR1 17.70054888 0.049343

C4orf17 -4.134941747 0.049357

CALCA 3.176223571 0.049358

AK093640 1.304754527 0.049367

SYT17 -1.797534959 0.049371

BC063684 1.02635142 0.049396

ADAMTS16 -8.025726194 0.049403

USP6 3.016922496 0.049419

PIGQ -0.857602514 0.049452

ENST00000397494 1.104857434 0.049461

H6PD -0.480266673 0.049463

A_24_P177634 1.814994967 0.049474

ENST00000338759 1.089902755 0.049475

FBN3 -0.547410087 0.049483

HS6ST1 -0.756060554 0.049495

PCDH19 -4.527243562 0.049497

A_32_P130217 1.716219607 0.049507

ZMAT2 -0.703796234 0.049513

ENST00000390256 3.28308057 0.049524

LOC388401 0.933384462 0.049552

AGT -58.49773418 0.049559

SNCAIP 4.428880135 0.049608

ADAT1 1.347001355 0.049642

LY96 0.828358266 0.049661

XR_018525 32.86993538 0.049666

A_32_P228570 -2.761393945 0.049675

CLRN3 -15.68608115 0.049679

BC007572 -0.420550778 0.049722

THC2489251 13.26956073 0.049723

CPA5 1.64368726 0.049724

MICAL1 0.697836824 0.049729

VHLL 7.843508241 0.049732

ENST00000391607 -0.832861064 0.04974

GRPEL1 1.545815298 0.049777

PKNOX1 1.143195199 0.049788

APPL2 1.498545308 0.049795

RPS2 0.851363157 0.049815

C10orf56 2.378204859 0.049825

ENST00000390312 0.626055845 0.049831

RGNEF 10.85301768 0.049837

TEX261 1.652517913 0.049843

ABCF2 1.29034259 0.049879

EMP2 2.295413756 0.04988

A_24_P303080 1.339851657 0.049915

CYP4F3 0.670392106 0.049927

PLA2G2F 1.672896171 0.049928

THC2692879 -5.041981738 0.049934

THC2565151 -0.543316857 0.049944

C14orf122 1.698912573 0.049946

CR616314 -2.033489436 0.049948

HIST1H3A 0.872857677 0.049969

**Supplemental Table 2 - Gene Set Enrichment Analysis Results**

NAME* SIZE ES† NES† NOM p-val FDR q-val Gene Sets Category*

GNF2_ANK1 37 -0.6191 -1.6555 0.0219 0.5001 C4

GNF2_SPTB 37 -0.6191 -1.6683 0.0189 0.5167 C4

V$PAX4_03 32 -0.7346 -2.0768 0.0045 0.5421 C3

MODULE_92 22 -0.7713 -2.1155 < 0.001 0.5450 C4

GNF2_TAL1 36 -0.6166 -1.6758 0.0220 0.5456 C4

GNF2_SPTA1 41 -0.6576 -1.7266 0.0049 0.5499 C4

MODULE_47 49 -0.5584 -1.6046 0.0329 0.5530 C4

GNF2_CDC27 30 -0.6182 -1.7524 0.0323 0.5549 C4

GCM_CALM1 26 -0.6791 -1.7723 0.0191 0.5763 C4

YTAATTAA_V$LHX3_01 27 -0.7278 -1.9269 0.0100 0.5830 C3

MODULE_204 15 -0.7507 -2.0242 0.0347 0.5971 C4

GCM_RAN 45 -0.5477 -1.5442 0.0335 0.6233 C4

GCM_NCAM1 32 -0.6531 -1.7820 0.0096 0.6351 C4

V$OLF1_01 39 -0.6246 -1.8207 0.0100 0.6647 C3

MORF_THPO 16 0.7042 1.6742 0.0299 0.6689 C4

MORF_KDR 15 0.7242 1.6991 0.0280 0.6841 C4

MORF_RAD51L3 54 0.5914 1.6508 0.0173 0.7208 C4

MORF_CDH4 15 0.7159 1.6769 0.0313 0.7235 C4

MORF_CAMK4 42 0.6111 1.7055 0.0112 0.7337 C4

V$MZF1_01 43 -0.5788 -1.7393 0.0155 0.7338 C3

V$AR_Q2 22 -0.7065 -1.9457 0.0487 0.7689 C3

V$AFP1_Q6 37 0.6953 1.8917 < 0.001 0.7811 C3

MORF_CD8A 17 0.7357 1.7138 0.0208 0.7856 C4

GNF2_RAD23A 35 -0.6764 -1.8035 0.0143 0.8170 C4

V$ZIC1_01 35 -0.6077 -1.6833 0.0426 0.8586 C3

TGAYRTCA_V$ATF3_Q6 85 -0.4805 -1.5394 0.0265 0.8592 C3

MORF_SUPT3H 46 0.6248 1.7199 0.0075 0.8756 C4

V$HNF4_01_B 39 -0.5535 -1.5824 0.0446 0.8947 C3

GNF2_MAP2K3 36 -0.6337 -1.8327 0.0111 0.8951 C4

GCATTTG,MIR-105 36 -0.5909 -1.6447 0.0370 0.9102 C3

MORF_PTPRB 37 0.5816 1.5579 0.0435 0.9118 C4

MORF_MAGEA9 64 0.5257 1.5097 0.0428 0.9227 C4

MORF_ERCC4 50 0.5594 1.5628 0.0434 0.9406 C4

MORF_MAP2K7 27 0.6787 1.7324 0.0218 0.9555 C4

MORF_FSHR 40 0.6492 1.7592 0.0098 0.9778 C4

V$CART1_01 26 0.7395 1.9026 0.0013 1.0000 C3

GNF2_BNIP3L 35 -0.6874 -1.8443 0.0049 1.0000 C4

CELL_CELL_ADHESION 17 0.7520 1.8023 0.0080 1.0000 C5

ACTGTGA,MIR-27A,MIR-27B 80 0.5488 1.6135 0.0087 1.0000 C3

MORF_CTSB 23 0.7197 1.7910 0.0090 1.0000 C4

HSA04540_GAP_JUNCTION 23 0.7340 1.8144 0.0091 1.0000 C2

MORF_MAGEA8 36 0.6641 1.7608 0.0102 1.0000 C4

LAL_KO_6MO_UP 16 0.7458 1.7770 0.0106 1.0000 C2

NEUROLOGICAL_SYSTEM_PROCESS 50 0.5998 1.6674 0.0114 1.0000 C5

V$NKX25_01 18 0.7374 1.8057 0.0129 1.0000 C3

MORF_IFNA1 29 0.6786 1.7733 0.0138 1.0000 C4

V$ER_Q6_02 33 0.6699 1.7574 0.0177 1.0000 C3

HSA05218_MELANOMA 17 0.7270 1.7468 0.0180 1.0000 C2

SYNAPTIC_TRANSMISSION 23 0.7030 1.7865 0.0181 1.0000 C5

V$GATA1_05 37 0.6408 1.7138 0.0198 1.0000 C3

EXTRACELLULAR_REGION 58 0.5803 1.6259 0.0199 1.0000 C5

V$PAX2_02 42 0.5997 1.6312 0.0254 1.0000 C3

V$GATA1_03 33 0.6347 1.6766 0.0269 1.0000 C3

HOFFMANN_BIVSBII_IMVM 16 -0.7544 -2.0446 0.0271 1.0000 C2

GH_GHRHR_KO_24HRS_UP 22 0.6735 1.6598 0.0326 1.0000 C2

AGEING_KIDNEY_SPECIFIC_UP 35 0.6017 1.6120 0.0335 1.0000 C2

V$GATA1_04 33 0.6200 1.6425 0.0335 1.0000 C3

V$MEIS1AHOXA9_01 16 0.6848 1.6604 0.0338 1.0000 C3

CCAGGTT,MIR-490 15 0.7131 1.6822 0.0355 1.0000 C3

MULTI_ORGANISM_PROCESS 32 -0.6280 -1.7382 0.0355 1.0000 C5

HSA04510_FOCAL_ADHESION 48 0.5614 1.5617 0.0362 1.0000 C2

TRANSMISSION_OF_NERVE_IMPULSE 26 0.6519 1.6566 0.0370 1.0000 C5

V$PR_01 19 0.6723 1.6703 0.0373 1.0000 C3

GATED_CHANNEL_ACTIVITY 17 0.6955 1.6671 0.0374 1.0000 C5

AGED_MOUSE_CEREBELLUM_UP 16 -0.7394 -2.1000 0.0380 1.0000 C2

ELONGINA_KO_DN 35 -0.5770 -1.6659 0.0390 1.0000 C2

V$NFKB_C 45 0.5632 1.5617 0.0412 1.0000 C3

V$CP2_02 34 0.5959 1.5986 0.0437 1.0000 C3

V$BRN2_01 30 0.6034 1.5740 0.0440 1.0000 C3

V$PAX8_B 15 0.7020 1.6370 0.0442 1.0000 C3

PASSERINI_SIGNAL 77 0.5006 1.4904 0.0454 1.0000 C2

V$FAC1_01 29 0.6229 1.6085 0.0456 1.0000 C3

V$CDX2_Q5 31 0.5994 1.5928 0.0476 1.0000 C3

V$RORA2_01 23 0.6483 1.6062 0.0481 1.0000 C3

HSA04912_GNRH_SIGNALING_PATHWAY 16 0.6941 1.6394 0.0481 1.0000 C2

*Full descriptions of gene sets and categories are available at http://www.broadinstitute.org/gsea/msigdb/collections.js

†Enrichment score (ES); normalized enrichment score (NES)

**Supplemental Table 3 - Gene Ontology Results**

Molecular Function

GO-ID p-value corr p-valuex n X N Description

5515 4.84E-19 8.66E-16 1558 8081 2570 15336 protein binding

5488 1.44E-10 1.29E-07 2173 12286 2570 15336 binding

16881 2.33E-06 1.39E-03 66 227 2570 15336 acid-amino acid ligase activity

3824 3.65E-06 1.63E-03 947 5062 2570 15336 catalytic activity

19787 7.84E-06 2.80E-03 57 194 2570 15336 small conjugating protein ligase activity

3924 3.60E-05 9.92E-03 58 208 2570 15336 GTPase activity

5083 4.34E-05 9.92E-03 75 289 2570 15336 small GTPase regulator activity

4842 4.44E-05 9.92E-03 50 173 2570 15336 ubiquitin-protein ligase activity

16879 7.24E-05 1.44E-02 67 255 2570 15336 ligase activity, forming carbon-nitrogen bonds

16773 1.21E-04 2.14E-02 150 677 2570 15336 phosphotransferase activity, alcohol group as acceptor

3846 1.32E-04 2.14E-02 5 5 2570 15336 2-acylglycerol O-acyltransferase activity

46983 1.48E-04 2.21E-02 130 576 2570 15336 protein dimerization activity

16301 1.94E-04 2.68E-02 160 736 2570 15336 kinase activity

4672 3.14E-04 4.01E-02 126 565 2570 15336 protein kinase activity

60589 3.95E-04 4.50E-02 98 424 2570 15336 nucleoside-triphosphatase regulator activity

30695 4.37E-04 4.50E-02 96 415 2570 15336 GTPase regulator activity

42802 4.44E-04 4.50E-02 148 685 2570 15336 identical protein binding

33613 4.53E-04 4.50E-02 6 8 2570 15336 transcription activator binding

44212 5.23E-04 4.93E-02 34 116 2570 15336 DNA regulatory region binding

Cellular Component

GO-ID p-value corr p-valuex n X N Description

5737 3.24E-22 2.52E-19 1486 7614 2687 16264 cytoplasm

44444 2.43E-20 9.42E-18 1054 5137 2687 16264 cytoplasmic part

5622 7.23E-19 1.87E-16 2047 11245 2687 16264 intracellular

44424 2.36E-18 4.59E-16 1990 10894 2687 16264 intracellular part

5829 3.03E-16 4.72E-14 327 1312 2687 16264 cytosol

43229 5.61E-14 7.27E-12 1707 9282 2687 16264 intracellular organelle

43226 8.46E-14 9.39E-12 1708 9296 2687 16264 organelle

43227 1.06E-11 1.03E-09 1534 8324 2687 16264 membrane-bounded organelle

43231 1.36E-11 1.17E-09 1532 8317 2687 16264 intracellular membrane-bounded organelle

44446 6.93E-10 5.38E-08 925 4795 2687 16264 intracellular organelle part

44422 9.73E-10 6.87E-08 935 4860 2687 16264 organelle part

31410 2.78E-08 1.68E-06 168 685 2687 16264 cytoplasmic vesicle

31982 2.81E-08 1.68E-06 174 715 2687 16264 vesicle

31988 5.22E-08 2.90E-06 163 666 2687 16264 membrane-bounded vesicle

16023 5.74E-08 2.97E-06 159 647 2687 16264 cytoplasmic membrane-bounded vesicle

44464 2.06E-07 1.00E-05 2533 14948 2687 16264 cell part

5623 2.19E-07 1.00E-05 2533 14949 2687 16264 cell

31090 3.87E-07 1.67E-05 386 1870 2687 16264 organelle membrane

5794 1.50E-06 6.15E-05 199 886 2687 16264 Golgi apparatus

45121 3.36E-06 1.31E-04 47 148 2687 16264 membrane raft

5856 6.72E-06 2.49E-04 291 1399 2687 16264 cytoskeleton

12505 8.39E-06 2.96E-04 296 1430 2687 16264 endomembrane system

44431 3.70E-05 1.25E-03 123 531 2687 16264 Golgi apparatus part

16323 4.77E-05 1.55E-03 57 208 2687 16264 basolateral plasma membrane

5768 5.24E-05 1.63E-03 93 383 2687 16264 endosome

32991 7.71E-05 2.31E-03 594 3158 2687 16264 macromolecular complex

5773 8.32E-05 2.40E-03 70 274 2687 16264 vacuole

44459 1.07E-04 2.96E-03 389 1997 2687 16264 plasma membrane part

5634 1.12E-04 3.01E-03 937 5173 2687 16264 nucleus

323 1.85E-04 4.64E-03 61 237 2687 16264 lytic vacuole

5764 1.85E-04 4.64E-03 61 237 2687 16264 lysosome

267 2.11E-04 5.14E-03 221 1077 2687 16264 cell fraction

786 2.83E-04 6.41E-03 24 71 2687 16264 nucleosome

5626 2.86E-04 6.41E-03 174 827 2687 16264 insoluble fraction

44433 2.89E-04 6.41E-03 67 270 2687 16264 cytoplasmic vesicle part

43228 3.40E-04 7.13E-03 461 2435 2687 16264 non-membrane-bounded organelle

43232 3.40E-04 7.13E-03 461 2435 2687 16264 intracellular non-membrane-bounded organelle

5901 3.83E-04 7.82E-03 18 48 2687 16264 caveola

32993 4.30E-04 8.42E-03 31 103 2687 16264 protein-DNA complex

5624 4.33E-04 8.42E-03 167 796 2687 16264 membrane fraction

48471 4.93E-04 9.34E-03 77 325 2687 16264 perinuclear region of cytoplasm

30055 5.79E-04 1.07E-02 27 87 2687 16264 cell-substrate junction

30141 8.00E-04 1.45E-02 48 186 2687 16264 stored secretory granule

31091 8.89E-04 1.55E-02 18 51 2687 16264 platelet alpha granule

43234 9.00E-04 1.55E-02 484 2595 2687 16264 protein complex

5924 1.21E-03 2.04E-02 25 82 2687 16264 cell-substrate adherens junction

15630 1.84E-03 2.98E-02 122 577 2687 16264 microtubule cytoskeleton

139 1.84E-03 2.98E-02 97 444 2687 16264 Golgi membrane

5813 1.88E-03 2.98E-02 41 159 2687 16264 centrosome

31983 2.03E-03 3.12E-02 14 38 2687 16264 vesicle lumen

44430 2.05E-03 3.12E-02 192 961 2687 16264 cytoskeletal part

9986 2.26E-03 3.37E-02 77 342 2687 16264 cell surface

44445 2.50E-03 3.67E-02 37 142 2687 16264 cytosolic part

31093 2.67E-03 3.85E-02 13 35 2687 16264 platelet alpha granule lumen

12506 3.13E-03 4.37E-02 57 243 2687 16264 vesicle membrane

48770 3.21E-03 4.37E-02 26 92 2687 16264 pigment granule

42470 3.21E-03 4.37E-02 26 92 2687 16264 melanosome

45335 3.26E-03 4.37E-02 8 17 2687 16264 phagocytic vesicle

60205 3.56E-03 4.65E-02 13 36 2687 16264 cytoplasmic membrane-bounded vesicle lumen

5925 3.59E-03 4.65E-02 23 79 2687 16264 focal adhesion

Biological Process

GO-ID p-value corr p-valuex n X N Description

9987 9.22E-13 5.11E-09 1740 9314 2426 14200 cellular process

23034 7.69E-09 1.42E-05 271 1161 2426 14200 intracellular signaling pathway

44237 7.72E-09 1.42E-05 971 4967 2426 14200 cellular metabolic process

23033 2.26E-08 3.13E-05 449 2103 2426 14200 signaling pathway

6464 3.45E-08 3.82E-05 338 1524 2426 14200 protein modification process

35556 7.29E-08 6.73E-05 201 835 2426 14200 intracellular signal transduction

43412 1.24E-07 9.22E-05 350 1605 2426 14200 macromolecule modification

44267 1.33E-07 9.22E-05 452 2148 2426 14200 cellular protein metabolic process

48518 1.96E-07 1.21E-04 461 2203 2426 14200 positive regulation of biological process

43687 2.21E-07 1.23E-04 283 1264 2426 14200 post-translational protein modification

65009 3.11E-07 1.57E-04 243 1063 2426 14200 regulation of molecular function

43068 4.10E-07 1.76E-04 117 444 2426 14200 positive regulation of programmed cell death

8152 4.43E-07 1.76E-04 1122 5926 2426 14200 metabolic process

44260 4.81E-07 1.76E-04 694 3498 2426 14200 cellular macromolecule metabolic process

48522 5.04E-07 1.76E-04 420 1999 2426 14200 positive regulation of cellular process

43065 5.07E-07 1.76E-04 116 441 2426 14200 positive regulation of apoptosis

10942 6.73E-07 2.19E-04 117 448 2426 14200 positive regulation of cell death

7264 7.86E-07 2.42E-04 83 292 2426 14200 small GTPase mediated signal transduction

51098 8.35E-07 2.43E-04 62 200 2426 14200 regulation of binding

1775 1.07E-06 2.95E-04 79 276 2426 14200 cell activation

23052 1.34E-06 3.54E-04 623 3126 2426 14200 signaling

43067 1.54E-06 3.87E-04 199 859 2426 14200 regulation of programmed cell death

10941 1.81E-06 4.18E-04 200 866 2426 14200 regulation of cell death

65007 1.81E-06 4.18E-04 1283 6899 2426 14200 biological regulation

16192 2.03E-06 4.50E-04 144 588 2426 14200 vesicle-mediated transport

44238 2.43E-06 5.18E-04 999 5262 2426 14200 primary metabolic process

42981 2.79E-06 5.72E-04 196 851 2426 14200 regulation of apoptosis

15031 3.01E-06 5.94E-04 177 756 2426 14200 protein transport

45184 3.26E-06 6.02E-04 179 767 2426 14200 establishment of protein localization

2682 3.26E-06 6.02E-04 110 428 2426 14200 regulation of immune system process

2376 3.90E-06 6.96E-04 214 947 2426 14200 immune system process

10033 4.43E-06 7.66E-04 199 872 2426 14200 response to organic substance

7165 5.79E-06 9.71E-04 389 1876 2426 14200 signal transduction

50789 6.00E-06 9.76E-04 1211 6512 2426 14200 regulation of biological process

51641 7.31E-06 1.13E-03 218 976 2426 14200 cellular localization

8219 7.32E-06 1.13E-03 164 701 2426 14200 cell death

6917 1.07E-05 1.56E-03 84 315 2426 14200 induction of apoptosis

16265 1.12E-05 1.56E-03 164 706 2426 14200 death

46907 1.14E-05 1.56E-03 156 666 2426 14200 intracellular transport

23060 1.16E-05 1.56E-03 438 2155 2426 14200 signal transmission

23046 1.16E-05 1.56E-03 438 2155 2426 14200 signaling process

19538 1.20E-05 1.56E-03 520 2605 2426 14200 protein metabolic process

12502 1.21E-05 1.56E-03 84 316 2426 14200 induction of programmed cell death

16043 1.44E-05 1.77E-03 512 2565 2426 14200 cellular component organization

50790 1.44E-05 1.77E-03 203 908 2426 14200 regulation of catalytic activity

6996 1.70E-05 2.05E-03 294 1387 2426 14200 organelle organization

43170 1.74E-05 2.05E-03 769 4005 2426 14200 macromolecule metabolic process

50794 1.83E-05 2.11E-03 1149 6184 2426 14200 regulation of cellular process

35466 2.20E-05 2.48E-03 220 1002 2426 14200 regulation of signaling pathway

33036 2.25E-05 2.49E-03 241 1112 2426 14200 macromolecule localization

30097 2.29E-05 2.49E-03 65 233 2426 14200 hemopoiesis

18193 2.52E-05 2.64E-03 48 158 2426 14200 peptidyl-amino acid modification

8104 2.53E-05 2.64E-03 204 921 2426 14200 protein localization

45321 2.65E-05 2.72E-03 65 234 2426 14200 leukocyte activation

45637 2.86E-05 2.88E-03 28 76 2426 14200 regulation of myeloid cell differentiation

50793 3.11E-05 3.08E-03 178 790 2426 14200 regulation of developmental process

48534 3.53E-05 3.43E-03 70 259 2426 14200 hemopoietic or lymphoid organ development

51649 3.60E-05 3.44E-03 192 864 2426 14200 establishment of localization in cell

51239 3.92E-05 3.68E-03 230 1063 2426 14200 regulation of multicellular organismal process

42221 3.99E-05 3.68E-03 306 1466 2426 14200 response to chemical stimulus

46578 4.43E-05 3.99E-03 64 233 2426 14200 regulation of Ras protein signal transduction

60548 4.47E-05 3.99E-03 97 389 2426 14200 negative regulation of cell death

44419 4.68E-05 4.11E-03 84 327 2426 14200 interspecies interaction between organisms

51100 4.93E-05 4.27E-03 28 78 2426 14200 negative regulation of binding

9056 5.02E-05 4.27E-03 218 1004 2426 14200 catabolic process

2761 5.22E-05 4.27E-03 20 48 2426 14200 regulation of myeloid leukocyte differentiation

6468 5.25E-05 4.27E-03 150 654 2426 14200 protein amino acid phosphorylation

51336 5.25E-05 4.27E-03 94 376 2426 14200 regulation of hydrolase activity

43069 5.34E-05 4.29E-03 95 381 2426 14200 negative regulation of programmed cell death

44262 5.84E-05 4.56E-03 94 377 2426 14200 cellular carbohydrate metabolic process

19318 5.85E-05 4.56E-03 53 185 2426 14200 hexose metabolic process

32879 5.98E-05 4.60E-03 164 727 2426 14200 regulation of localization

46777 6.41E-05 4.86E-03 28 79 2426 14200 protein amino acid autophosphorylation

45595 7.27E-05 5.44E-03 129 552 2426 14200 regulation of cell differentiation

2520 8.35E-05 6.17E-03 72 275 2426 14200 immune system development

43066 8.76E-05 6.38E-03 93 376 2426 14200 negative regulation of apoptosis

22607 9.02E-05 6.49E-03 200 919 2426 14200 cellular component assembly

51049 9.48E-05 6.61E-03 121 515 2426 14200 regulation of transport

2263 9.61E-05 6.61E-03 17 39 2426 14200 cell activation involved in immune response

2366 9.61E-05 6.61E-03 17 39 2426 14200 leukocyte activation involved in immune response

51101 9.77E-05 6.61E-03 46 157 2426 14200 regulation of DNA binding

22603 9.79E-05 6.61E-03 77 300 2426 14200 regulation of anatomical structure morphogenesis

5996 1.02E-04 6.82E-03 59 216 2426 14200 monosaccharide metabolic process

2521 1.08E-04 7.11E-03 39 127 2426 14200 leukocyte differentiation

90046 1.15E-04 7.40E-03 41 136 2426 14200 regulation of transcription regulator activity

51090 1.15E-04 7.40E-03 41 136 2426 14200 regulation of transcription factor activity

12501 1.28E-04 8.17E-03 133 579 2426 14200 programmed cell death

10646 1.42E-04 8.95E-03 243 1153 2426 14200 regulation of cell communication

6810 1.62E-04 9.94E-03 503 2573 2426 14200 transport

51056 1.62E-04 9.94E-03 70 271 2426 14200 regulation of small GTPase mediated signal transduction

18105 1.63E-04 9.94E-03 14 30 2426 14200 peptidyl-serine phosphorylation

8624 1.71E-04 1.03E-02 34 108 2426 14200 induction of apoptosis by extracellular signals

45596 1.77E-04 1.05E-02 62 234 2426 14200 negative regulation of cell differentiation

44093 1.89E-04 1.12E-02 144 640 2426 14200 positive regulation of molecular function

30301 2.02E-04 1.17E-02 17 41 2426 14200 cholesterol transport

15918 2.02E-04 1.17E-02 17 41 2426 14200 sterol transport

51234 2.06E-04 1.17E-02 508 2607 2426 14200 establishment of localization

44248 2.16E-04 1.22E-02 170 776 2426 14200 cellular catabolic process

48523 2.27E-04 1.26E-02 368 1837 2426 14200 negative regulation of cellular process

6950 2.28E-04 1.26E-02 357 1777 2426 14200 response to stress

23051 2.31E-04 1.27E-02 170 777 2426 14200 regulation of signaling process

42116 2.40E-04 1.30E-02 10 18 2426 14200 macrophage activation

16310 2.44E-04 1.31E-02 171 783 2426 14200 phosphorylation

6007 2.66E-04 1.41E-02 20 53 2426 14200 glucose catabolic process

6325 2.69E-04 1.42E-02 99 417 2426 14200 chromatin organization

44275 2.77E-04 1.43E-02 27 81 2426 14200 cellular carbohydrate catabolic process

9611 2.77E-04 1.43E-02 124 543 2426 14200 response to wounding

6915 2.90E-04 1.49E-02 129 569 2426 14200 apoptosis

6006 2.97E-04 1.51E-02 42 146 2426 14200 glucose metabolic process

23014 3.06E-04 1.53E-02 88 364 2426 14200 signal transmission via phosphorylation event

7243 3.06E-04 1.53E-02 88 364 2426 14200 intracellular protein kinase cascade

9966 3.30E-04 1.63E-02 168 772 2426 14200 regulation of signal transduction

48583 3.60E-04 1.76E-02 120 526 2426 14200 regulation of response to stimulus

7249 3.71E-04 1.79E-02 22 62 2426 14200 I-kappaB kinase/NF-kappaB cascade

46365 3.72E-04 1.79E-02 23 66 2426 14200 monosaccharide catabolic process

30099 3.79E-04 1.79E-02 30 95 2426 14200 myeloid cell differentiation

30098 3.79E-04 1.79E-02 30 95 2426 14200 lymphocyte differentiation

42346 4.09E-04 1.92E-02 8 13 2426 14200 positive regulation of NF-kappaB import into nucleus

51128 4.15E-04 1.92E-02 122 538 2426 14200 regulation of cellular component organization

51093 4.19E-04 1.92E-02 72 289 2426 14200 negative regulation of developmental process

6066 4.19E-04 1.92E-02 101 432 2426 14200 alcohol metabolic process

48519 4.25E-04 1.93E-02 397 2011 2426 14200 negative regulation of biological process

51246 4.33E-04 1.95E-02 141 636 2426 14200 regulation of protein metabolic process

5975 4.61E-04 2.06E-02 118 519 2426 14200 carbohydrate metabolic process

51099 4.70E-04 2.08E-02 33 109 2426 14200 positive regulation of binding

34637 4.77E-04 2.09E-02 23 67 2426 14200 cellular carbohydrate biosynthetic process

46649 4.99E-04 2.17E-02 50 186 2426 14200 lymphocyte activation

18209 5.03E-04 2.18E-02 16 40 2426 14200 peptidyl-serine modification

10604 5.16E-04 2.22E-02 198 937 2426 14200 positive regulation of macromolecule metabolic process

10608 5.28E-04 2.25E-02 62 243 2426 14200 posttranscriptional regulation of gene expression

34613 5.42E-04 2.29E-02 101 435 2426 14200 cellular protein localization

30183 5.48E-04 2.30E-02 17 44 2426 14200 B cell differentiation

51186 5.91E-04 2.46E-02 54 206 2426 14200 cofactor metabolic process

51179 6.41E-04 2.65E-02 569 2980 2426 14200 localization

70887 6.49E-04 2.66E-02 94 402 2426 14200 cellular response to chemical stimulus

70727 6.95E-04 2.79E-02 101 438 2426 14200 cellular macromolecule localization

43393 6.96E-04 2.79E-02 16 41 2426 14200 regulation of protein binding

7266 6.96E-04 2.79E-02 16 41 2426 14200 Rho protein signal transduction

31293 7.32E-04 2.91E-02 7 11 2426 14200 membrane protein intracellular domain proteolysis

18198 7.47E-04 2.95E-02 5 6 2426 14200 peptidyl-cysteine modification

50817 7.68E-04 2.99E-02 31 103 2426 14200 coagulation

7596 7.68E-04 2.99E-02 31 103 2426 14200 blood coagulation

43392 7.81E-04 3.02E-02 22 65 2426 14200 negative regulation of DNA binding

43433 7.96E-04 3.03E-02 20 57 2426 14200 negative regulation of transcription factor activity

90048 7.96E-04 3.03E-02 20 57 2426 14200 negative regulation of transcription regulator activity

7265 7.99E-04 3.03E-02 33 112 2426 14200 Ras protein signal transduction

44085 8.09E-04 3.05E-02 216 1041 2426 14200 cellular component biogenesis

31589 8.19E-04 3.06E-02 30 99 2426 14200 cell-substrate adhesion

51704 8.43E-04 3.10E-02 168 787 2426 14200 multi-organism process

51642 8.50E-04 3.10E-02 4 4 2426 14200 centrosome localization

51770 8.50E-04 3.10E-02 4 4 2426 14200 positive regulation of nitric-oxide synthase biosynthetic process

43001 8.50E-04 3.10E-02 4 4 2426 14200 Golgi to plasma membrane protein transport

45471 8.84E-04 3.20E-02 25 78 2426 14200 response to ethanol

6886 9.22E-04 3.31E-02 86 366 2426 14200 intracellular protein transport

65008 9.47E-04 3.38E-02 308 1542 2426 14200 regulation of biological quality

51716 9.69E-04 3.42E-02 206 991 2426 14200 cellular response to stimulus

6955 9.91E-04 3.42E-02 135 617 2426 14200 immune response

42345 9.97E-04 3.42E-02 11 24 2426 14200 regulation of NF-kappaB import into nucleus

32570 9.97E-04 3.42E-02 11 24 2426 14200 response to progesterone stimulus

32374 9.97E-04 3.42E-02 11 24 2426 14200 regulation of cholesterol transport

32371 9.97E-04 3.42E-02 11 24 2426 14200 regulation of sterol transport

16044 1.00E-03 3.42E-02 89 382 2426 14200 cellular membrane organization

6968 1.01E-03 3.42E-02 21 62 2426 14200 cellular defense response

8284 1.01E-03 3.42E-02 105 463 2426 14200 positive regulation of cell proliferation

7599 1.02E-03 3.42E-02 32 109 2426 14200 hemostasis

61024 1.09E-03 3.65E-02 89 383 2426 14200 membrane organization

6796 1.14E-03 3.75E-02 197 946 2426 14200 phosphate metabolic process

6793 1.14E-03 3.75E-02 197 946 2426 14200 phosphorus metabolic process

1817 1.15E-03 3.77E-02 52 202 2426 14200 regulation of cytokine production

44087 1.19E-03 3.87E-02 43 160 2426 14200 regulation of cellular component biogenesis

44282 1.20E-03 3.87E-02 65 265 2426 14200 small molecule catabolic process

44270 1.23E-03 3.95E-02 22 67 2426 14200 cellular nitrogen compound catabolic process

9894 1.24E-03 3.95E-02 68 280 2426 14200 regulation of catabolic process

19320 1.27E-03 4.03E-02 21 63 2426 14200 hexose catabolic process

30218 1.27E-03 4.03E-02 16 43 2426 14200 erythrocyte differentiation

43277 1.30E-03 4.06E-02 6 9 2426 14200 apoptotic cell clearance

51251 1.30E-03 4.06E-02 31 106 2426 14200 positive regulation of lymphocyte activation

43388 1.34E-03 4.17E-02 28 93 2426 14200 positive regulation of DNA binding

51051 1.37E-03 4.22E-02 43 161 2426 14200 negative regulation of transport

43087 1.39E-03 4.24E-02 38 138 2426 14200 regulation of GTPase activity

33124 1.39E-03 4.24E-02 38 138 2426 14200 regulation of GTP catabolic process

7010 1.42E-03 4.32E-02 101 447 2426 14200 cytoskeleton organization

6334 1.43E-03 4.33E-02 27 89 2426 14200 nucleosome assembly

48041 1.48E-03 4.45E-02 8 15 2426 14200 focal adhesion assembly

16052 1.53E-03 4.58E-02 31 107 2426 14200 carbohydrate catabolic process

43933 1.57E-03 4.67E-02 155 729 2426 14200 macromolecular complex subunit organization

2684 1.62E-03 4.79E-02 65 268 2426 14200 positive regulation of immune system process

1818 1.69E-03 4.97E-02 16 44 2426 14200 negative regulation of cytokine production

51249 1.70E-03 4.98E-02 42 158 2426 14200 regulation of lymphocyte activation

**Supplemental Table 4 - Cell Type Expression from BioGPS**

Gene B lymphoblasts CD105+ CD33 CD34 CD71 CD4 CD8 Dentritic Cells CD14 CD19 CD56 Thymus marrow

HSPA12A 4.361 1.125 1.262 1.194 0.960 0.989 1.058 1.135 1.088 1.088 1.213 1.009 1.055

MBP 1.192 1.081 1.000 1.151 2.066 1.031 1.025 0.985 0.889 0.864 1.673 0.828 1.172

MICB 2.602 0.712 1.445 0.563 0.286 0.824 1.265 1.265 1.001 1.804 3.121 0.679 0.567

FLJ14712 0.837 0.906 0.782 0.876 1.022 0.898 0.939 0.909 0.892 0.901 0.848 1.127 1.118

PI16 0.874 1.078 0.783 0.756 1.038 0.930 0.941 0.922 0.901 0.954 0.849 1.514 1.130

ADAM23 1.103 1.096 1.034 1.172 0.965 1.076 1.069 0.959 1.090 1.062 1.007 0.827 0.835

CFH 1.255 1.190 1.308 1.438 1.041 1.160 1.130 1.053 1.166 1.154 1.172 0.876 0.893

GOLGB1 5.181 1.097 5.119 4.387 0.784 3.967 5.501 5.912 2.164 1.793 4.093 0.994 0.734

HARS2 1.777 0.435 0.700 0.891 0.297 2.416 1.929 0.949 0.913 1.634 1.660 0.506 0.244

PHF17 1.177 1.302 0.791 3.759 1.666 2.065 2.340 0.999 0.906 1.252 4.588 1.073 1.090

CHD2 1.067 1.025 1.179 1.117 0.975 1.049 1.086 0.908 1.123 1.025 1.197 0.865 0.914

RBM5 1.396 1.832 2.513 3.194 0.782 3.715 4.561 2.632 1.677 2.254 2.279 1.154 0.717

LARP5 5.406 1.255 2.771 2.778 2.183 2.747 2.856 3.922 2.658 1.790 3.253 2.117 1.507

AP3M1 21.070 2.614 2.988 10.875 1.030 3.629 5.125 9.176 3.093 3.638 8.981 1.128 1.116

EIF4G2 1.298 1.126 1.465 1.248 1.283 1.248 1.545 1.182 1.222 1.156 1.182 0.823 0.874

USP34 1.519 1.606 2.977 8.377 1.226 14.445 18.812 4.617 1.676 2.032 4.896 1.409 1.368

KIAA0515 0.881 0.897 0.809 0.804 0.993 0.915 0.958 0.906 0.906 0.924 0.866 1.151 1.103

RP11-345P4. 1.168 1.109 1.394 1.844 1.000 3.184 3.637 1.968 1.175 1.722 1.636 1.722 0.876

NCOA6 2.189 2.122 5.609 3.046 0.545 2.575 3.365 3.478 1.837 2.452 4.995 0.971 0.274

ZCCHC3 0.856 0.901 0.789 0.812 1.023 0.901 0.934 0.912 0.901 0.901 0.845 1.123 1.123

ZNF398 0.960 0.891 0.723 0.881 0.951 1.000 2.158 0.911 0.821 0.831 0.812 1.247 1.050

C18orf17 0.794 0.705 1.785 0.661 0.802 2.544 4.435 1.071 0.883 0.777 2.623 0.883 0.884

GPR171 0.241 0.226 0.245 0.247 0.200 8.627 14.811 0.334 0.228 0.223 1.610 0.169 0.178

CLDND1 3.301 0.480 0.851 0.834 1.087 4.757 6.629 2.470 0.675 0.391 1.297 0.321 0.226

P2RY5 0.133 0.137 0.818 0.104 0.084 0.774 0.391 0.458 1.806 0.089 0.594 0.071 0.072

FANK1 0.846 0.848 0.711 0.954 1.025 0.822 0.891 0.886 0.840 0.866 0.821 1.160 1.102

LRRN3 0.956 1.133 1.207 1.154 0.917 11.054 30.962 0.982 1.119 1.047 1.095 0.799 0.949

FUCA1 0.916 3.715 5.488 0.618 0.138 0.357 0.237 1.693 7.497 0.293 0.820 1.415 0.933

AHRR 4.238 0.941 0.785 1.031 1.119 0.891 0.936 0.911 0.926 0.968 0.818 1.114 1.110

LOC157627 1.532 1.706 1.602 1.747 2.675 1.484 1.254 0.906 0.977 0.961 1.483 0.762 1.893

P2RY6 0.699 1.057 1.198 1.064 0.723 0.847 0.757 1.963 1.069 1.034 0.867 0.759 0.803

PID1 0.212 0.255 0.698 0.224 0.212 0.219 0.194 0.265 2.739 0.213 0.241 0.196 0.199

SASH1 1.218 1.029 1.798 1.107 0.890 1.155 1.093 1.139 1.466 1.015 1.107 1.661 1.626

AIM1L 1.249 1.129 0.891 0.949 1.134 1.138 1.102 1.071 0.981 0.994 0.957 1.123 1.113

Cell type

PSTPIP1 0.127 0.068 1.587 0.113 0.057 1.282 1.032 0.384 1.966 0.329 2.287 0.067 0.092

GMIP 0.242 0.047 0.488 0.106 0.038 0.469 0.391 0.617 1.435 0.823 1.229 0.095 0.084

LSP1 0.511 0.017 0.490 0.088 0.038 0.270 0.401 0.579 0.960 0.413 0.506 0.068 0.137

MBD6 0.908 0.666 0.717 0.799 0.919 0.633 0.666 0.690 0.587 0.728 0.500 0.864 0.916

PLCG2 1.979 0.166 0.753 0.319 0.125 0.130 0.197 0.901 0.849 1.726 1.791 0.235 0.329

TKT 0.681 0.403 1.893 0.834 0.073 0.133 0.133 0.536 1.635 0.373 0.371 0.225 0.375

KIT 1.446 4.638 2.072 95.575 0.999 1.512 1.506 1.343 1.512 1.531 4.819 0.982 1.500

JARID1B 1.113 1.666 1.363 7.994 1.154 1.309 1.115 1.122 1.129 1.253 1.146 1.936 0.887

SYF2 0.562 0.472 0.860 0.578 0.294 0.820 0.760 0.789 0.815 1.186 0.965 0.223 0.170

CCNL1 1.935 2.938 18.651 5.726 1.701 11.572 10.815 8.882 4.417 5.154 5.220 0.313 0.103

FBXO11 1.584 3.049 1.380 2.827 1.011 2.162 2.447 1.183 1.451 1.029 1.102 0.950 0.988

OSBPL9 5.876 1.534 1.768 2.876 1.178 1.526 1.735 2.705 2.380 2.171 1.901 0.953 1.225

AGTPBP1 1.092 1.043 2.679 1.154 0.926 1.061 1.037 1.006 1.197 1.025 3.123 0.741 0.741

SNX13 1.082 1.284 4.500 1.536 0.967 1.088 1.318 1.170 1.462 1.029 1.106 0.808 0.884

MNDA 0.017 0.023 0.542 0.024 0.005 0.001 0.001 0.094 0.385 0.007 0.024 0.001 0.023

RHOA 0.599 0.317 0.830 0.473 0.221 0.475 0.501 0.752 0.867 0.477 0.718 0.636 0.373

ST3GAL6 1.000 1.173 1.579 1.519 1.033 1.495 1.033 1.040 1.189 1.160 1.083 0.811 0.887

KCNE3 0.718 0.748 2.466 0.674 0.933 0.740 0.771 0.822 8.205 0.740 0.704 0.911 0.926

LAMP2 0.228 0.417 1.265 0.972 0.225 0.165 0.214 0.686 0.851 0.193 0.520 0.256 0.367

PARP8 0.719 0.759 1.802 0.683 0.801 4.936 4.884 0.700 1.029 0.870 1.679 0.896 0.909

AQP9 0.007 0.003 0.075 0.003 0.003 0.003 0.003 0.004 0.154 0.003 0.003 0.003 0.008

TLR1 0.031 0.016 0.226 0.033 0.013 0.017 0.015 0.095 0.356 0.029 0.131 0.011 0.015

PLXNC1 0.027 0.025 0.434 0.116 0.022 0.044 0.036 0.328 0.408 0.040 0.056 0.018 0.024

TLR6 0.868 0.613 1.510 0.672 0.592 0.596 0.642 0.753 1.243 0.805 0.732 0.451 0.596

FBXL5 0.148 0.167 0.967 0.172 0.143 0.191 0.188 0.259 0.631 0.171 0.399 0.119 0.128

IQGAP1 0.508 0.074 3.994 0.703 0.044 0.922 0.988 3.929 2.116 2.137 6.350 0.107 0.066

BACH1 1.198 1.099 1.562 1.190 0.950 1.091 1.083 1.190 1.132 1.083 1.174 0.819 0.819

EGLN1 0.168 0.130 0.317 0.130 0.102 0.153 0.131 0.137 0.154 0.128 0.174 0.092 0.088

ZBTB34 0.864 0.898 0.784 0.806 1.021 1.078 0.942 0.908 0.886 0.898 0.852 1.204 1.158

C12orf35 0.102 0.251 0.351 0.115 0.118 0.772 1.112 0.167 0.296 0.397 0.616 0.026 0.023

EVI2A 0.017 0.012 0.607 0.030 0.006 0.311 0.313 0.241 0.696 0.173 0.367 0.032 0.007

NIN 1.163 1.118 1.068 1.203 1.160 1.155 1.012 0.867 1.272 1.244 1.238 0.705 0.940

ZNF281 2.136 1.374 2.183 1.770 0.757 1.689 1.643 4.395 4.863 4.625 6.242 0.600 0.608

LCP2 0.192 0.086 0.891 0.585 0.072 2.087 1.951 0.427 0.700 0.086 1.449 0.402 0.487

N4BP2L2 2.307 1.920 8.228 5.366 1.162 18.374 31.765 3.268 2.860 2.371 3.539 2.240 0.941

PPFIA1 1.369 1.411 5.155 2.206 1.474 2.217 1.914 1.951 2.357 1.381 3.158 0.857 1.206

UBR2 1.054 1.006 0.789 0.993 1.218 3.095 2.163 1.258 1.149 1.081 1.215 1.071 1.071

TGFBR2 0.296 0.415 0.530 0.386 0.301 1.576 2.005 0.978 0.740 0.918 0.746 0.335 0.294

MSL3L1 2.462 1.187 3.021 1.472 0.997 5.205 4.108 2.776 2.129 1.525 1.761 0.888 0.972

C15orf29 0.360 0.374 0.396 0.509 0.320 0.327 0.338 0.363 0.347 0.333 0.372 0.248 0.266

FAM8A1 0.025 0.178 0.319 0.162 0.046 0.196 0.439 0.112 0.078 0.087 0.846 0.059 0.038

BRCA1 3.448 5.367 2.117 4.103 3.457 1.108 1.058 1.698 1.345 1.233 2.190 1.255 2.436

PHF20L1 0.863 0.874 1.412 0.958 0.970 1.001 1.300 1.238 0.875 0.983 1.001 1.046 1.029

SP3 1.236 0.921 1.286 1.156 0.760 1.213 1.425 1.444 1.071 0.573 2.040 0.613 0.574

ACTR3 1.186 0.534 1.132 0.706 0.601 0.678 0.787 0.946 0.827 0.978 1.893 0.224 0.207

GOLGA7 0.719 0.728 1.243 0.973 0.327 1.037 0.986 0.921 1.129 0.628 1.746 0.131 0.085

PURB 2.035 2.403 2.102 2.177 2.358 2.038 1.790 2.408 2.127 1.909 1.574 2.054 2.347

ZC3H11A 0.962 0.942 1.297 1.544 0.644 1.438 1.823 1.435 0.749 1.286 1.721 0.677 0.214

NUP153 2.538 2.459 0.855 2.070 1.777 1.074 1.293 0.469 0.280 1.004 1.443 0.399 0.135

MORC3 0.146 1.389 0.959 0.340 0.514 0.588 0.732 0.379 0.458 0.997 0.981 0.115 0.105

PUM2 2.159 0.608 1.267 1.739 0.877 2.193 2.609 1.246 0.950 1.356 2.091 0.410 0.186

RNF111 0.237 0.123 0.564 0.265 0.040 0.413 0.426 0.376 0.430 0.345 0.344 0.124 0.112

UBQLN2 0.392 0.341 1.581 0.821 0.088 2.254 2.196 0.994 1.215 1.312 2.067 0.759 0.224

AGER 1.283 1.166 1.258 1.614 1.578 1.283 1.503 1.308 1.179 1.381 1.197 0.847 0.853

JHDM1D 0.124 0.205 1.533 0.182 1.577 0.779 0.966 0.647 0.513 0.754 0.224 0.087 0.130

BCORL1 0.633 1.069 1.304 1.070 0.830 0.897 0.893 0.847 0.866 0.755 1.154 0.834 1.302

NOTCH1 0.498 0.517 0.646 0.634 0.571 0.638 0.629 0.552 0.703 0.525 0.772 0.810 0.618

MAEA 1.150 0.977 0.690 1.023 1.260 2.539 2.242 1.705 1.190 0.627 1.924 0.562 0.236

POLR2C 1.539 1.404 1.597 1.610 1.103 1.339 1.093 1.331 1.221 1.614 1.655 1.413 0.860

CSGALNACT 0.153 0.251 0.279 0.252 0.166 1.815 0.415 0.688 0.495 2.787 0.254 0.176 0.602

TMEM45B 0.855 0.896 0.785 0.806 1.014 1.021 0.979 0.930 0.875 0.944 0.833 1.118 1.111

RNF34 1.188 0.632 0.355 0.612 0.233 0.900 1.056 0.749 0.460 0.405 1.135 0.557 0.210

TBRG1 0.948 0.927 0.803 0.972 1.004 2.398 2.831 0.893 0.914 0.896 0.879 1.028 0.931

TESK2 1.278 1.119 1.214 1.148 1.633 1.231 1.018 1.414 0.569 5.580 1.710 0.900 0.994

AATK 0.195 0.239 0.911 0.351 0.407 0.207 0.200 0.205 0.658 0.251 0.494 0.210 0.583

CNOT3 2.296 1.176 1.232 1.264 1.000 1.103 1.120 1.752 1.992 1.121 1.240 0.847 0.848

BAT2 3.677 2.463 1.876 2.882 1.488 1.966 1.792 2.044 2.418 1.768 1.965 1.356 1.007

LOC90379 4.060 1.109 1.187 1.178 1.725 2.394 2.667 1.391 1.736 1.546 1.405 0.842 0.851

NPFF 1.240 1.109 1.316 1.394 0.968 1.761 1.327 1.164 1.120 1.163 1.217 0.837 0.818

RDH5 1.190 0.924 1.015 1.134 1.006 1.125 0.971 0.726 0.843 0.942 0.808 0.997 0.834

HCP5 0.854 0.032 0.121 0.037 0.032 0.485 0.454 0.261 0.135 0.446 0.570 0.105 0.132

SSH1 1.199 0.638 1.112 0.724 0.642 0.811 0.802 0.848 0.835 0.807 2.046 0.868 0.711

ZFYVE1 1.408 1.521 1.527 2.646 3.859 1.556 1.172 1.006 0.964 1.083 2.479 1.130 1.059

HSPA1L 0.615 0.601 0.983 0.593 0.652 1.043 0.735 0.561 0.861 0.632 0.974 0.784 0.869

PARP16 1.355 1.405 1.210 1.243 0.981 1.492 1.568 1.131 1.080 1.637 1.344 0.959 0.853

SAP130 1.242 0.360 0.863 1.402 0.901 0.758 0.711 1.402 0.435 0.774 1.722 0.638 0.261

PPP2R2A 2.187 0.476 0.660 0.630 0.496 1.015 1.040 0.745 0.875 1.047 0.773 0.423 0.227

VAMP3 0.205 0.702 2.216 0.677 0.285 0.375 0.326 0.945 2.383 0.399 0.826 0.343 0.645

ADIPOR1 0.559 0.401 0.655 0.402 2.106 0.239 0.194 0.521 0.427 0.484 0.290 0.253 1.377

C20orf108 0.526 38.134 4.646 8.070 90.705 2.612 1.804 3.190 4.310 2.165 2.565 1.349 3.774

ALAS2 1.198 224.916 1.210 1.176 1430.888 1.099 1.077 1.121 1.077 1.078 1.166 0.835 262.529

NFIX 2.508 3.794 2.466 2.770 3.155 2.385 1.680 2.164 1.450 1.130 1.992 0.834 0.866

FECH 6.192 82.173 1.618 11.621 545.107 1.450 1.743 9.003 1.243 1.125 2.924 3.042 28.822

TRAK2 1.738 22.568 2.763 4.338 86.947 3.606 3.695 1.559 1.582 6.888 5.998 0.817 3.354

TSPAN5 1.024 4.111 1.087 1.039 47.417 1.568 1.182 1.125 1.007 1.881 1.681 0.977 4.276

TMEM111 1.105 1.095 1.272 1.133 2.438 1.061 0.927 1.072 1.116 1.145 1.122 0.833 0.911

PRDX6 1.614 3.677 1.668 2.871 8.234 1.390 1.770 2.375 1.547 1.685 1.346 1.538 1.173

ALDH5A1 3.278 5.560 2.033 9.939 5.076 3.134 4.102 11.077 1.309 4.554 2.469 10.854 1.575

FZD5 2.096 17.438 1.370 3.275 15.725 1.082 1.020 1.234 1.174 1.124 1.264 0.917 1.011

C5orf4 0.469 3.566 0.949 0.978 28.341 0.667 0.750 0.740 1.129 1.097 0.795 0.855 2.676

ITLN1 0.859 0.926 0.778 0.800 5.187 0.897 0.912 0.919 0.934 0.904 0.911 3.009 1.119

SLC14A1 0.893 1.194 1.241 1.146 13.422 1.000 0.871 1.077 1.202 1.125 1.082 0.824 1.430

OPTN 1.442 1.642 0.382 0.422 4.080 3.637 2.827 0.597 0.340 1.196 4.680 0.359 0.696

TESC 0.852 9.866 3.024 1.234 16.824 0.374 0.440 0.565 2.777 0.754 1.477 0.472 1.948

CREG1 0.354 1.958 3.670 2.054 1.305 0.148 0.104 0.339 2.433 0.187 0.192 0.145 0.353

RGS10 1.616 1.185 1.838 1.286 1.617 1.383 1.424 1.162 1.183 1.105 1.305 2.123 0.829

POP7 6.400 9.246 2.051 5.451 2.972 0.837 1.037 2.214 3.228 2.563 3.930 0.767 0.572

RBX1 2.292 2.434 1.052 1.325 4.958 0.812 0.801 1.663 1.666 1.071 1.021 1.033 0.595

IGF2 1.198 6.125 1.486 1.189 1.582 1.112 1.108 1.208 1.148 1.110 1.217 2.571 3.045

LGR6 1.304 1.344 0.856 0.808 1.536 1.128 2.121 1.144 1.024 0.896 5.864 1.336 1.120

CYB5R3 2.182 1.528 1.659 1.954 2.984 0.553 0.622 1.634 1.481 1.559 1.699 1.200 0.835

DCUN1D1 1.446 1.864 0.797 1.002 8.932 0.440 0.409 0.940 0.585 0.407 2.180 0.169 0.176

GPR20 1.168 1.103 1.215 1.168 1.140 1.093 1.074 1.140 1.103 1.093 1.177 0.831 0.831

RRAS 0.193 0.199 0.771 0.174 0.203 0.210 0.179 0.333 0.712 0.185 0.618 0.265 0.199

FLCN 1.056 1.093 0.849 0.897 1.919 1.318 1.338 0.853 0.964 0.940 0.934 0.989 1.148

NT5M 1.215 1.115 1.069 3.338 4.676 1.031 0.992 0.931 1.000 1.031 1.077 0.793 0.823

PDZK1IP1 0.761 1.769 0.941 0.922 80.802 0.851 0.737 0.836 0.898 0.906 0.749 0.655 7.537

TAC3 1.320 1.293 0.844 0.823 2.225 1.197 0.959 1.055 0.973 0.959 1.314 0.980 1.286

RAB6A 1.278 1.147 1.171 1.249 4.572 1.200 1.227 1.674 1.303 1.041 2.290 1.066 0.894

SPHK2 1.988 0.838 1.211 1.162 0.925 1.187 1.075 1.217 1.062 1.460 1.174 0.789 0.776

ABCC3 1.236 1.111 1.229 1.688 0.979 1.070 1.070 1.251 1.381 1.111 1.209 0.862 0.826

ENDOD1 1.956 3.387 1.839 3.247 21.377 1.498 1.610 2.286 1.358 3.878 1.978 0.788 0.911

SPARC 0.839 1.656 0.781 1.241 1.979 0.424 0.469 0.542 0.589 0.699 1.067 7.434 0.994

MGLL 9.168 1.425 0.506 2.694 0.663 0.268 0.239 3.103 1.186 0.239 0.378 2.311 2.108

SLC6A4 1.061 1.008 1.201 1.210 0.982 1.113 0.965 1.000 1.079 1.069 1.175 0.842 0.956

VIL1 5.390 1.175 1.216 1.135 0.813 1.140 0.929 0.982 1.099 0.900 1.053 0.883 0.883

FSTL1 1.737 4.410 1.036 1.151 0.553 0.494 0.585 0.983 0.764 0.536 0.676 4.179 0.986

PPBP 0.001 0.067 0.001 0.217 0.003 0.002 0.001 0.092 0.261 0.177 0.009 0.001 0.158

PF4 0.002 0.046 0.002 0.250 0.002 0.002 0.002 0.033 0.134 0.055 0.002 0.002 0.043

TUBA8 1.162 1.485 1.219 1.161 0.981 1.096 1.067 1.096 1.105 1.105 1.162 0.828 0.838

C3orf60 3.732 2.851 1.354 2.713 1.620 0.880 0.969 2.122 1.369 1.151 1.651 1.398 0.463

QPRT 59.764 3.854 1.506 5.135 0.978 1.123 1.079 2.169 1.336 1.145 1.887 1.303 0.809

HEBP1 0.071 25.796 4.222 5.286 24.312 0.568 0.433 2.470 5.672 1.262 0.815 0.727 2.134

ATP5S 4.775 3.999 2.639 3.557 5.479 1.979 2.065 3.052 1.624 3.835 4.876 0.985 1.113

RAB27B 0.851 1.212 1.321 1.231 1.185 1.122 0.991 1.149 1.355 1.353 1.380 0.778 1.007

CDKN1C 1.244 1.155 1.277 1.244 0.999 1.166 1.156 1.189 7.146 1.145 11.718 0.877 0.844

NEURL 1.220 1.298 1.392 2.047 1.013 2.405 1.126 1.183 2.183 1.146 1.786 0.829 0.893

NR4A1 1.560 1.141 2.292 1.160 1.582 1.347 1.075 4.426 1.790 1.167 1.955 2.343 1.328

GZMB 0.020 0.019 0.021 0.021 0.017 0.029 0.126 8.530 0.060 0.019 7.189 0.014 0.035

PRSS23 0.682 0.652 0.701 0.755 0.619 0.772 0.728 0.626 0.658 0.668 7.567 0.960 0.599

SPON2 0.139 0.144 0.156 0.272 0.090 0.423 1.928 2.215 0.135 0.236 72.560 0.224 0.295

SLC1A7 1.293 1.173 1.443 1.219 1.052 1.201 1.086 1.402 1.109 1.391 1.297 0.825 1.122

SPN 2.044 2.234 2.108 2.304 1.823 2.589 2.994 1.835 1.709 1.627 7.007 1.418 1.063

AR 1.197 1.103 1.237 1.186 0.963 1.104 1.073 1.242 1.103 1.093 1.174 0.841 0.826

LIPC 1.257 1.110 1.227 1.188 0.960 1.139 1.109 1.158 1.128 1.109 1.227 0.851 0.831

MLC1 1.207 5.510 1.890 35.635 1.085 1.158 1.085 1.145 1.329 1.085 56.946 0.829 21.907

SETBP1 1.175 0.959 1.200 1.067 1.059 1.075 1.109 11.036 1.142 10.111 2.667 2.292 1.242

STX1A 1.115 1.106 1.256 1.026 0.867 1.168 1.089 1.089 1.106 1.089 1.239 0.858 0.840

**Supplemental Table 5 - Genes Evaluated by qRT-PCR**

Gene Coef p value

LRRN3 -2.362145 1.89E-41

CLDND1 -6.881561 2.01E-38

SASH1 -1.975006 2.46E-33

P2RY6 -1.838935 5.04E-30

MUC1 -2.267995 2.04E-23

AR 1.030172 1.51E-21

AF289562 -1.586619 1.19E-19

FOXP3 -1.67793 5.78E-19

CD161 1.316636 1.27E-13

MS4A7 1.284539 2.01E-13

C5orf4 0.883721 2.37E-13

CX3CR1 1.50864 4.16E-13

ALS2CR2 1.00853 5.89E-13

CD62P 0.856222 7.98E-13

FANK1 -0.927216 2.65E-12

PDGFD 1.080855 1.92E-11

GFOD1 1.450604 3.80E-10

CMTM5 0.781631 4.75E-10

GPR56 0.789194 1.26E-09

SOAT2 -0.58953 4.59E-09

SPECC1 1.162844 6.02E-09

FAM20B 1.185878 1.51E-08

CCND2 -1.951501 2.68E-08

COL13A1 0.363943 2.96E-08

SLAMF7 1.084464 4.15E-08

TPOR 0.580105 5.14E-08

TNXB 0.685784 5.97E-08

IL7R -1.232798 1.07E-07

GZMB 0.712889 2.11E-07

PECR -2.13196 2.30E-07

GAPDH -1.626898 3.47E-07

C6ORF25 0.600886 4.07E-07

RHCE 0.452825 4.70E-07

ITGA2B 0.493752 5.44E-07

AGPAT3 1.615248 6.26E-07

S100A12 -0.587828 1.06E-06

CENTB5 -2.126874 1.33E-06

NISCH -2.812456 1.38E-06

GNAS 1.076408 2.03E-06

AF086547 -0.60394 2.13E-06

SERPINB2 -0.661928 2.63E-06

CD34 0.37606 4.07E-06

SCML4 -1.199545 9.06E-06

SIRT2 2.593271 1.04E-05

NCF4 -0.874931 1.20E-05

SAMD3 0.796454 1.71E-05

ABCG1 -0.643668 1.79E-05

CCL5 0.813326 2.30E-05

PLCD1 -1.615633 2.91E-05

NF1 -1.57099 3.55E-05

DGKA -1.22251 4.44E-05

ILK 1.338602 4.96E-05

HMOX1 0.879501 5.10E-05

SNRPA -2.292061 5.18E-05

S100A8 -0.707042 5.50E-05

LEF1 -0.750473 5.76E-05

RABGAP1L 0.74965 9.19E-05

NELL2 -0.637243 9.80E-05

ABCA1 -0.489489 0.000105

CXXC1 -2.445703 0.000125

TNFRSF17 -0.349575 0.000135

TXNDC5 -0.516939 0.000138

TRAF2 -1.601953 0.000138

EXOC3L2 0.409337 0.000161

CD83 0.82831 0.000183

SPOCK2 -1.089319 0.000223

TOP2A 0.72923 0.00025

PPP2R2B 0.536948 0.000255

VCAN -0.77892 0.000261

DGKH -1.07057 0.000268

HIST1H2AC0.781118 0.0003

B3GAT1 0.322753 0.000303

CD177 -0.15666 0.000321

HDAC10 -1.734692 0.000333

NBPF11 -1.214131 0.000342

SREBF1 -0.728915 0.000391

LITAF -0.736358 0.000493

AGER -1.010192 0.000604

AQP9 -0.570397 0.000662

CD44 -1.508708 0.000737

SECISBP2 1.436398 0.00079

TP53BP1 -1.63046 0.000854

ND3 0.558518 0.000943

HNRNPF -1.784915 0.000953

LEMD2 -1.684687 0.001038

HDLBP 1.083323 0.001222

MYLIP -0.741063 0.001331

CASP5 -0.324159 0.001467

CDCA7 -0.630007 0.001492

SIRT4 -0.795162 0.001528

CD3D -0.702406 0.001624

RPL28 -1.299012 0.00205

GPX4 0.773612 0.002229

CREB1 1.304194 0.002563

MBOAT7 -0.636592 0.002602

TNFRSF10 -0.550878 0.002679

IL8RB -0.539887 0.002753

COX4I1 -1.449124 0.002774

TNFAIP6 -0.307325 0.002779

LPCAT1 1.097351 0.002984

PAPD4 1.272153 0.003069

IFNAR1 -0.694688 0.003189

NONO -2.319074 0.003302

SORT1 0.734604 0.00333

LOC44046 -0.49962 0.003359

CALML4 0.904992 0.00339

CD86 0.769155 0.004253

MRPL11 -1.245461 0.004348

SERGEF -1.356897 0.005673

KLRC4 0.320717 0.006971

NME1 -0.734372 0.007152

CAPN10 -1.270439 0.008124

TARDBP -1.789888 0.008787

SEPX1 -0.513506 0.009196

ARG1 -0.228219 0.009366

EMR1 0.379768 0.011597

ASGR1 -0.484339 0.013873

TFCP2 -1.545057 0.014271

CXCR5 -0.313983 0.014474

NMI -0.516693 0.015379

CD163 -0.44676 0.016598

SSRP1 -1.101836 0.017567

MTCH1 1.101073 0.018083

STX10 -0.650587 0.019017

CD11B -0.511374 0.025499

ANP32C -0.517131 0.027931

CR596746 -0.766784 0.030089

ESR1 0.291789 0.032398

PACSIN2 -0.495708 0.034721

FTH1 0.478031 0.035142

LRP1 -0.529362 0.040156

VEZF1 -1.187381 0.041842

DIAPH1 1.106751 0.042711

BPNT1 -0.990621 0.043597

ANAPC5 1.044725 0.048432

AKNA -0.910345 0.05323

IRF7 -0.317267 0.053712

RBM17 -1.085795 0.053771

ZNF16 -0.707178 0.053811

AKAP8L 0.932281 0.054365

ZNF3 0.975457 0.064329

CRIP1 -0.396513 0.066486

CYP4F2 -0.262408 0.067766

CCL2 -0.130644 0.070023

LDLR -0.362047 0.070068

SYNE1 0.308904 0.070719

APEX1 -0.723005 0.072279

DUSP6 0.473976 0.07523

RNPS1 -0.714714 0.078355

AMN -0.298288 0.079459

LTK 0.202095 0.084458

C5AR1 -0.340836 0.084672

PLA2G6 -0.643262 0.093157

HMGN3 0.573731 0.093711

TLR4 -0.308198 0.094244

IL13RA1 -0.307958 0.107862

OPRL1 -0.406763 0.112219

CD63 -0.498638 0.113714

TFIP11 -0.563891 0.11598

CCR7 -0.24345 0.132528

RORC -0.183713 0.139466

DIAPH2 -0.561725 0.141325

EPHB1 -0.254255 0.152456

WDR18 -0.442647 0.152913

IL3RA 0.155023 0.157646

E01979 -0.443912 0.159566

APOL4 0.418776 0.161136

UCK2 0.47875 0.162985

IL18RAP -0.142147 0.163015

HNRNPH2 -0.407944 0.163731

SILV 0.168749 0.164026

SPIB -0.16143 0.174602

IQCE 0.337128 0.185772

ACOX2 0.125069 0.18655

TLR2 -0.228895 0.18933

F11R 0.354585 0.194929

OSBPL6 0.122876 0.19733

CCNB1 0.303313 0.199436

NUDT16 0.361077 0.199981

NCOR2 0.728839 0.202105

MRPS12 -0.457134 0.203003

IL6 -0.114509 0.20308

PDGFRB 0.136732 0.207474

CLEC4E 0.169438 0.208942

TMC8 -0.404727 0.208989

IL17RC 0.179069 0.21023

MCM3 -0.497236 0.21656

ANKRD55 0.212798 0.217603

CD8 -0.134727 0.223371

ITPK1 0.155946 0.229015

SRP9 0.320274 0.235537

PFKFB2 0.251099 0.23831

OLIG1 0.165389 0.259221

CD45RA -0.399453 0.269837

ELF1 -0.465932 0.271067

CAT 0.199146 0.295935

KCNE1 0.139259 0.304951

NDUFA1 0.503365 0.307454

KCNE3 -0.208109 0.311792

LIN9 0.335687 0.311854

CD1D -0.252932 0.312426

NDUFS7 0.59384 0.319079

ACLY 1.166048 0.328459

SPHAR 0.289599 0.337919

ABLIM1 -0.230358 0.35229

NEFH 0.153443 0.355257

CD84 -0.307849 0.366692

ADORA3 -0.089597 0.367518

COX6C 0.249671 0.394432

ENPP3 -0.09894 0.405542

RPP14 -0.538673 0.40625

TMTC3 -0.31461 0.409383

IFI27 0.040369 0.416179

SFRS10 0.426344 0.422428

CR2 0.091775 0.434652

SORCS3 -0.062503 0.442948

SF3B5 0.544336 0.462956

APOE -0.111755 0.465729

POLD3 0.312084 0.466673

TMEFF2 0.100921 0.471686

GPER -0.062983 0.477928

HDC 0.079314 0.47812

PDK1 -0.201645 0.501095

AF161365 -0.032388 0.515556

IL4 -0.063198 0.520644

MPEG1 0.17294 0.534749

ATP5G3 0.272305 0.608262

TMED9 -0.310013 0.608444

SETD2 -0.252531 0.61591

PTX3 -0.094358 0.62827

CD33 0.090551 0.635637

AK2 -0.257191 0.668919

NRCAM -0.026501 0.693293

RAD54B -0.066664 0.693342

GOPC 0.245621 0.695794

CD79B 0.051089 0.726769

RP9 -0.109025 0.740952

DTD1 -0.10948 0.745203

LY75 0.081425 0.752011

CD19 -0.032948 0.769774

CD248 -0.030978 0.780322

GALNT3 -0.069112 0.787232

MFSD9 -0.038115 0.796109

IFIT3 -0.016627 0.79626

MSN 0.096493 0.808842

RAB32 0.069315 0.816735

CCR3 0.019031 0.827105

CPA3 0.023137 0.842032

TREML4 0.006631 0.856137

GUSB 0.084486 0.868694

DPYSL4 0.00656 0.899941

GATA2 0.013395 0.923839

PECAM1 -0.01239 0.953254

MIER1 -0.007706 0.976276

LRRC25 -0.004088 0.987313

ARHGEF10 0.000203 0.999319
