# Supplementary material for: A whole blood gene expression-based signature for smoking status
Source: BMC Med Genomics. 2012 Dec 3;5:58. doi: 10.1186/1755-8794-5-58 (PMC3538056; doi:10.1186/1755-8794-5-58)
Supplement: Additional file 2 — Figure S1. ROC analysis of gene expression score (GES) and cotinine. [file 1755-8794-5-58-S2.doc]

Supplemental Figure 1

(A)

(B)

(C)

Supplemental Figure 1 Legend: ROC analysis of gene expression algorithm

and cotinine. (A) Cross-validated AUC of gene expression algorithm in the

development set (n=1071) (B) AUC of gene expression algorithm in validation set

(n=180); (C) AUC of cotinine in validation set (n = 180).
